# Selective activation of PFKL suppresses the phagocytic oxidative burst

**DOI:** 10.1016/j.cell.2021.07.004

**Published:** 2021-07-27

**Authors:** Neri Amara, Madison P. Cooper, Maria A. Voronkova, Bradley A. Webb, Eric M. Lynch, Justin M. Kollman, Taylur Ma, Kebing Yu, Zijuan Lai, Dewakar Sangaraju, Nobuhiko Kayagaki, Kim Newton, Matthew Bogyo, Steven T. Staben, Vishva M. Dixit

**Affiliations:** 1 Physiological Chemistry Department, Genentech, South San Francisco, CA 94080, USA; 2 Biochemistry Department, West Virginia University, Morgantown, WV 26506, USA; 3 Biochemistry Department, University of Washington, Seattle, WA 98195, USA; 4 Microchemistry, Proteomics, and Lipidomics Department, Genentech, South San Francisco, CA 94080, USA; 5 Drug Metabolism and Pharmacokinetics Department, Genentech, South San Francisco, CA 94080, USA; 6 Pathology Department, Stanford University, Stanford, CA 94305, USA; 7 Discovery Chemistry Department, Genentech, South San Francisco, CA 94080, USA; 8 Lead contact

## Abstract

In neutrophils, nicotinamide adenine dinucleotide phosphate (NADPH) generated via the pentose phosphate pathway fuels NADPH oxidase NOX2 to produce reactive oxygen species for killing invading pathogens. However, excessive NOX2 activity can exacerbate inflammation, as in acute respiratory distress syndrome (ARDS). Here, we use two unbiased chemical proteomic strategies to show that small-molecule LDC7559, or a more potent designed analog NA-11, inhibits the NOX2-dependent oxidative burst in neutrophils by activating the glycolytic enzyme phosphofructokinase-1 liver type (PFKL) and dampening flux through the pentose phosphate pathway. Accordingly, neutrophils treated with NA-11 had reduced NOX2-dependent outputs, including neutrophil cell death (NETosis) and tissue damage. A high-resolution structure of PFKL confirmed binding of NA-11 to the AMP/ADP allosteric activation site and explained why NA-11 failed to agonize phosphofructokinase-1 platelet type (PFKP) or muscle type (PFKM). Thus, NA-11 represents a tool for selective activation of PFKL, the main phosphofructokinase-1 isoform expressed in immune cells.

## In brief

The small molecule LDC7559 and its more potent analog, NA-11, suppress excessive NOX2-dependent oxidative burst and NETosis, as well as subsequent tissue damage and inflammation, without compromising basal ROS production. They selectively activate the glycolytic enzyme phosphofructokinase-1 liver type (PFKL) to suppress glycolytic flux through the pentose phosphate pathway that leads to NOX2-dependent outputs.

## Graphical Abstract

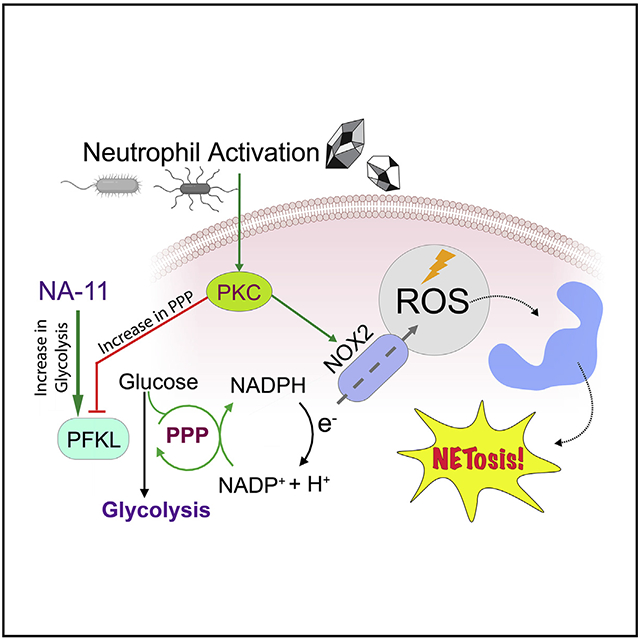

## INTRODUCTION

Phagocytes produce bactericidal reactive oxygen species (ROS) within the phagosome in an oxidative burst. The rapid increase in ROS is mediated by NOX2, a nicotinamide adenine dinucleotide phosphate (NADPH)-dependent oxygen reductase (Thomas, 2017). Assembly of the NOX2 complex on phagosome and cellular membranes is accompanied by an increase in oxygen consumption and glucose uptake (Zatti and Rossi, 1965). Glucose catabolism through the pentose phosphate pathway increases production of NADPH, which provides NOX2 the reducing equivalents needed to generate superoxide radicals.

Neutrophils are professional phagocytes that are essential for optimal antimicrobial defense and comprise 50%–70% of circulating leukocytes in humans (Mayadas et al., 2014). They rely on the oxidative burst for a multitude of functions, including phagocytosis (Rosales and Uribe-Querol, 2017), degranulation (Sengeløv et al., 1995), ROS production (Amulic et al., 2012), and formation of neutrophil extracellular traps (NETs) (Brinkmann, 2018; Brinkmann et al., 2004). Mutations inactivating the NOX2 complex impair the oxidative capacity of neutrophils and cause chronic granulomatous disease (CGD) (Roos et al., 2010). Patients with CGD are vulnerable to recurrent, chronic, and invasive bacterial and fungal infections (Heyworth et al., 2003).

Although neutrophils are crucial for innate immunity, excessive neutrophil activation can be deleterious. Local tissue damage, inflammation, and autoantigens stemming from NETs exacerbate the pathology of chronic conditions such as atherosclerosis, psoriasis, gout, and lupus (Brinkmann, 2018). Targeting the oxidative burst may have therapeutic potential, but there are safety concerns with inhibitors of NOX2 or enzymes of the pentose phosphate pathway, including glucose-6-phosphate dehydrogenase (G6PDH). Barriers to their use include suppression of innate immunity and general toxicity (Diebold et al., 2015; Kowalik et al., 2017).

NETosis of neutrophils is crucial for the killing of extracellular bacteria (Brinkmann et al., 2004), but the underlying molecular mechanisms remain largely unknown. Most physiological stimuli, including bacteria, fungi, and crystalline particulates, trigger NOX2-dependent NETosis, but some bacterial toxins acting as potassium and calcium ionophores promote NOX2-independent NETosis (Kenny et al., 2017). NOX2-dependent NETosis is described as a two-phase process (Neubert et al., 2020). During phase 1, active signaling cascades trigger a NOX2-induced oxidative burst, and histone-modifying enzymes such as neutrophil elastase (NE) and peptidyl-arginine deaminase 4 (PAD4) enter the nucleus. Phase 2 involves the entropic swelling of chromatin, rupture of the cell membrane, and dissemination of NETs composed of chromatin and granule proteins.

Inhibitors of NETosis may control chronic neutrophil-driven diseases. Recent phenotypic screening of a large chemical library in human neutrophils identified tool compound LDC7559 as an inhibitor of NOX2-dependent NETosis (Sollberger et al., 2018). It was proposed to target the pore-forming domain of gasdermin D (GSDMD), a protein that mediates a lytic form of cell death called pyroptosis. In macrophages, cleavage of GSDMD by human caspase-1, caspase-4, or caspase-5 (mouse caspase-1 or caspase-11) releases an N-terminal fragment that forms pores in membranes (Kayagaki and Dixit, 2019). In neutrophils, caspase-4 cleaves GSDMD in response to cytosolic lipopolysaccharide (LPS), leading to the extrusion of NETs (Chen et al., 2018). GSDMD may also be cleaved by neutrophil-specific proteases such as NE and cathepsin G (Burgener et al., 2019; Kambara et al., 2018). How LDC7559 inhibited GSDMD to prevent NETosis, and why LDC7559 inhibited NOX2-dependent NETosis, but not NOX2-independent NETosis, remained enigmatic.

We validated the activity of the pyrazolo-oxazepine LDC7559 in neutrophils and confirmed its ability to inhibit NETosis in the low-micromolar range. However, LDC7559 did not inhibit GSDMD. We designed a series of LDC7559 derivatives to improve on its potency and facilitate identification of its target. We found that the pyrazolo-oxazepine agonized the glycolytic enzyme phosphofructokinase-1 liver type (PFKL) to suppress the NOX2-dependent oxidative burst.

## RESULTS

### LDC7559 inhibits NETosis independent of GSDMD

Consistent with LDC7559 inhibiting NETosis (Sollberger et al., 2018), human peripheral blood neutrophils treated with 10 μM LDC7559 exhibited defective NETosis in response to phorbol 12-myristate 13-acetate (PMA) when compared with neutrophils treated with DMSO vehicle (Figure S1A). LDC7559 impaired chromatin expansion, citrullination of histone H3, and co-localization of myeloperoxidase with chromatin (Figure S1A). We quantified NETosis by measuring chromatin morphology and total area (van der Linden et al., 2017). Approximately 5%–10% of cells underwent spontaneous NETosis, whereas PMA induced NETosis in 70%–80% of neutrophils within 3 h (Figure S1B). LDC7559 inhibited PMA-induced NETosis in a dose-dependent manner but did not prevent spontaneous NETosis (Figures S1B and S1C).

**Figure 1.**
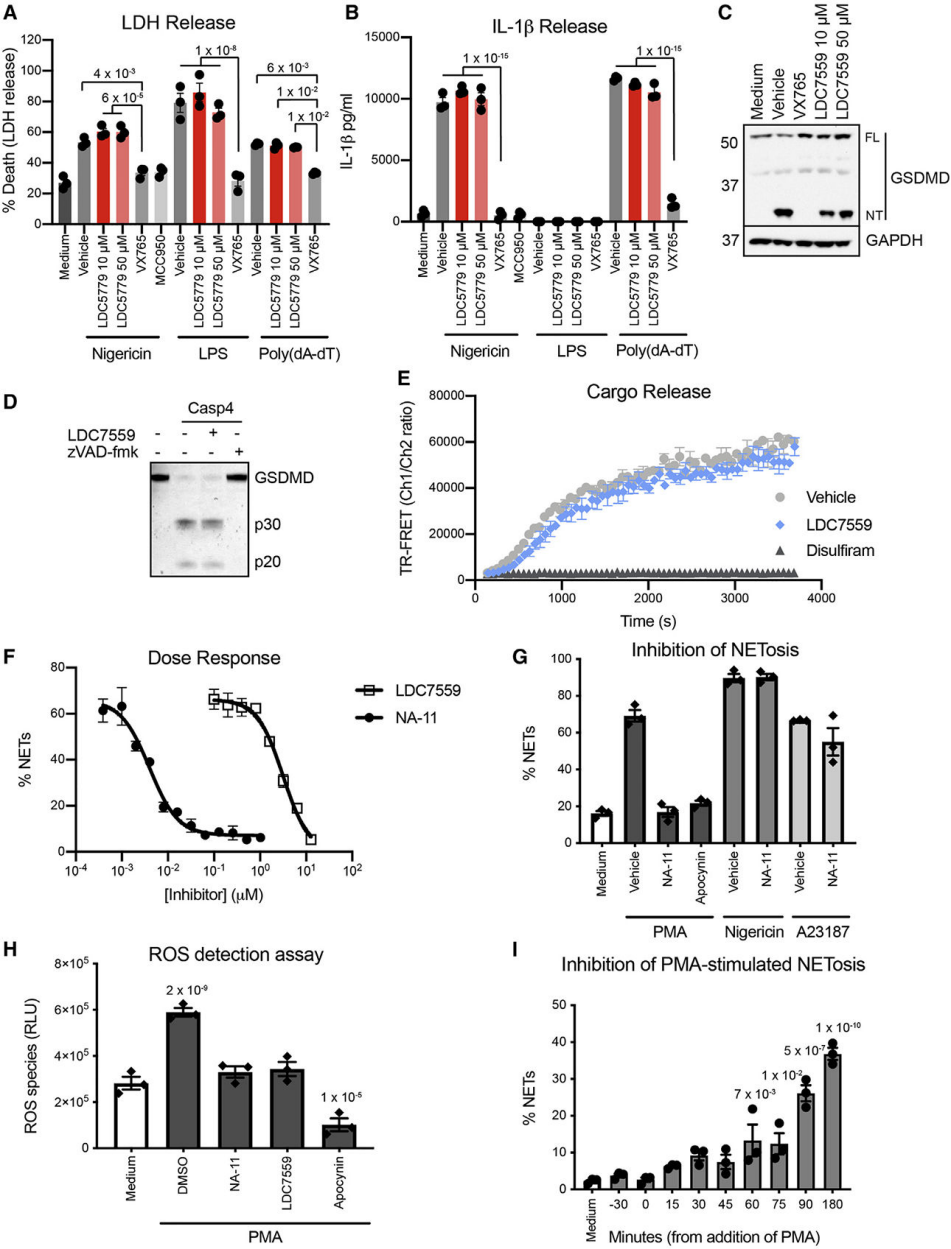
LDC7559 and NA-11 inhibit NETosis independent of GSDMD but elicit identical phenotypes in neutrophils (A and B) LDH (A) or IL-1β (B) released from primary human monocytes. Bars show the mean ± SEM of monocytes from three donors. (C) Western blots of monocytes at 1 h after electroporation with LPS. FL, full length GSDMD; NT, N-terminal fragment of GSDMD. Results representative of three independent experiments. (D) Coomassie blue staining of recombinant GSDMD. Results representative of three independent experiments. (E) TR-FRET assay measuring Europium-labeled biotin released from liposomes exposed to caspase-4 and GSDMD. Symbols indicate the mean ± SD of three independent experiments. (F and G) Percentage of polymorphonuclear leukocytes (PMNs) undergoing NETosis induced by PMA (F) or the stimuli indicated (G). Data are the mean ± SD of cells from three donors. (H) ROS production by PMNs. Bars show the mean ± SEM of PMNs from three donors. p values (two-way ANOVA, means compared to medium alone) are shown if p < 0.05. (I) Percentage of PMNs undergoing PMA-induced NETosis. The x axis indicates when NA-11 was added relative to the addition of PMA (t = 0). Bars show the mean ± SEM of PMNs from three donors. p values (one-way ANOVA, means compared to medium alone) are shown if p < 0.05. See also Figure S1.

LDC7559 was reported to block the pore-forming activity of GSDMD (Sollberger et al., 2018). We tested its ability to inhibit GSDMD-dependent pyroptosis in primary human monocytes and human monocytic THP-1 cells (Figures 1A-1C and S1D). Using lactate dehydrogenase (LDH) release to monitor cell death, LDC7559 did not alter GSDMD-dependent pyroptosis induced by nigericin, cytoplasmic LPS, or cytoplasmic poly(deoxyadenosine-deoxythymidine) (poly(dA-dT)) (Figures 1A and S1D). The GSDMD-dependent release of interleukin-1β (IL-1β) from monocytes in response to nigericin or poly(dA-dT) also occurred normally in the presence of LDC7559 (Figures 1B and S1D). Western blotting confirmed that LDC7559 did not prevent LPS-induced cleavage of GSDMD (Figure 1C). Accordingly, LDC7559 did not inhibit cleavage of recombinant GSDMD by caspase-4 (Figure 1D), nor did it prevent the GSDMD pore-forming fragment from permeabilizing liposomes (Figure 1E). In contrast, inhibition of GSDMD with disulfiram (Hu et al., 2020) prevented liposome permeabilization (Figure 1E). We conclude that LDC7559 does not inhibit GSDMD.

### Identification of NA-11, a potent analog of LDC7559

We initiated a combinatorial target identification approach using orthogonal chemical proteomic methods to identify the target(s) of LDC7559. We used photoaffinity labeling (PAL) and label-free thermal proteome profiling (TPP). The micromolar activity of LDC7559 in our phenotypic assay posed a limitation for the design of a PAL probe, because modifications with photoreactive linkers typically reduce the affinity for target engagement. We wanted a high-affinity probe to lower the concentration needed for full target engagement and limit off-target binding. Therefore, analogs with variant substitution on the pyrazolo-oxepine scaffold were tested for improved activity in the NETosis assay (Figure S1E). Replacement or removal of the acetamide was not tolerated, as NA-2 and NA-4 displayed no inhibitor activity. NA-6 and NA-7 with substitution at the meta and para positions, respectively, showed a slight increase in inhibitory activity, while unsubstituted NA-5 was ~10-fold more potent compared to LDC7559. NA-8, which lacked the phenyl ring, was inactive. Larger substitutions, as in NA-9 and NA-10, were largely tolerated at the meta position. Strikingly, a 4-pentynol substituent at the ortho position in NA-11 improved inhibition of NETosis significantly (half maximal inhibitory concentration (IC_50_) = 4 ± 0.5 nM); NA-11 was ~1,000-fold more potent than LDC7559 (Figure 1F).

We confirmed that NA-11 mimicked LDC7559 by inhibiting NOX2-dependent NETosis induced by PMA, but not NOX2-independent NETosis induced by the ionophores nigericin and calcimycin (A23187) (Figure 1G). Both LDC7559 and NA-11 also inhibited PMA-induced ROS production in neutrophils (Figure 1H). Like LDC7559 (Sollberger et al., 2018), NA-11 inhibited NETosis when added up to 1 h after PMA-induced neutrophil activation, after which NETosis became irreversible (Figure 1I). Finally, neither LDC7559 nor NA-11 inhibited recombinant NE (Figure S1F). Collectively, these data suggest that NA-11 and LDC7559 likely engage the same target to inhibit NETosis in neutrophils.

### Identification of PFKL as the main target of NA-11

**Figure 2.**
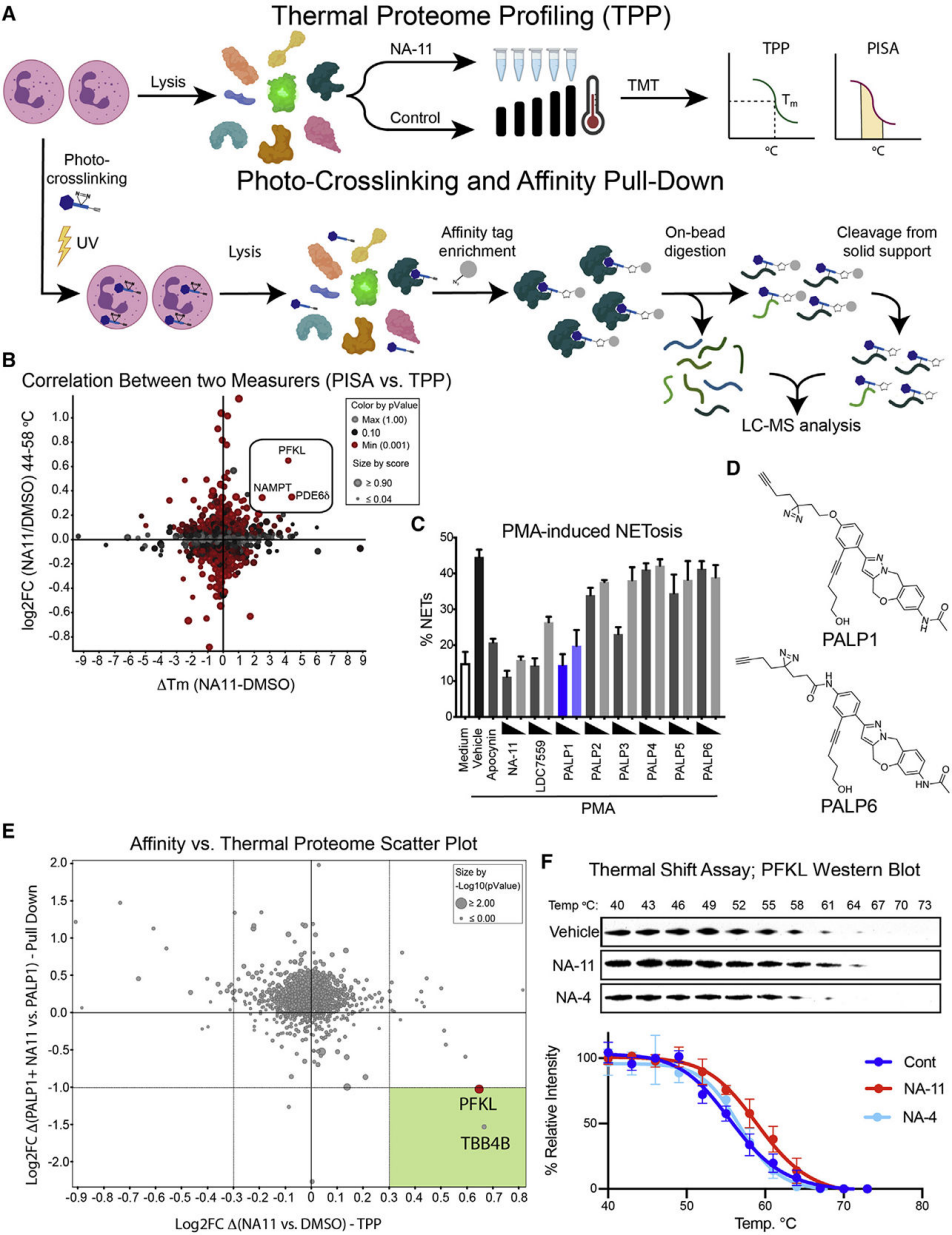
Chemoproteomic analysis of the global interactors and specific targets of NA-11 (A) Dual chemoproteomic workflow. (B) TPP analysis of neutrophil lysates. Graph plots the ΔTm induced by NA-11 between 37°C and 65°C versus log2(fold change in the area under the melting curve from 44°C to 58°C). Each circle represents a different protein. Top hits with an upward shift in Tm are boxed. p values were determined by paired Student’s t test. (C) Percentage of PMNs undergoing PMA-induced NETosis. Bars show the mean ± SD of cells from three donors. (D) Structures of PALP1 and PALP6. (E) Scatterplot shows the TPP (x axis) and affinity pull-down data (y axis; fold change of affinity pull-down using PALP1 ± NA-11). Dot size indicates the p values from the affinity pull-downs (paired Student’s t test). The green quadrant shows proteins that are selectively targeted by NA-11 in both methods. (F) Western blots of THP-1 lysates heated for 3 min. Graph indicates PFKL band intensities quantified using ImageJ. Data are the mean ± SD of three independent experiments. See also Figures S2 and S3.

We assessed global interactors of NA-11 by TPP coupled with 16-plex tandem mass tag (TMT) isobaric labeling and mass spectrometry. Neutrophil lysates were treated with NA-11, and changes in protein thermal stability, compared to vehicle, were evaluated across the proteome from 37°C to 65°C (Figure 2A). We identified 5,316 unique proteins and generated eight-point melting curves for ~3,000 proteins. One of the proteins most affected by NA-11 was the glycolytic enzyme PFKL, with a thermal shift of 4°C in melting temperature (Tm) (Figure S2A). We also assayed proteome integral solubility alteration (PISA) (Gaetani et al., 2019) from 44°C to 58°C, measuring the difference in the area under the curve between NA-11- and vehicle-treated samples (Figures S2B-S2D; Data S2). A correlation between these two thermal stability measurers highlighted three proteins (PFKL, nicotinamide phosphoribosyltransferase [NAMPT], and PDE6δ) with an upward shift in Tm as potential interactors of NA-11 (Figure 2B).

In our search for the optimal photoreactive probe, we synthesized several PAL probes (PALPs) based on the NA-11 scaffold (Figures 2C, 2D, and S2E). PALP1 best inhibited NETosis (Figure 2C). Its structure closely resembles that of NA-11, with a light-activatable diazirine and an alkyne tag appended at the para position of the phenyl ring through an ether bond (Figure 2D). PALP1 had an IC_50_ value of 271 ± 41 nM, which is ~60-fold higher than that of NA-11 but more than 10-fold lower than that of LDC7559 (Figure S2F). PALP6, which differed from PALP1 only by an amide bond instead of an ether bond at the para position, showed no inhibition of NETosis, suggesting complete loss of affinity to the target (Figures 2C and 2D). Therefore, PALP6 served as a negative control in subsequent photo-crosslinking experiments.

Human blood leukocytes were pooled from multiple donors and incubated with PALP1, PALP6, DMSO vehicle, or PALP1 with 10-fold excess NA-11. We expected a high-affinity binding target would be enriched by PALP1 only and competed by NA-11. The live cells were UV irradiated to induce crosslinking and lysed, and then an affinity tag was conjugated to the probes. Target proteins were affinity purified and identified by mass spectrometry (Figure 2A; Data S1). Multiple proteins were enriched in the PALP1 sample, but a limited number were absent in the negative controls (Figure S3A). PFKL was a prominent target for PALP1 and the only protein that was competed effectively by NA-11. Importantly, PALP6 showed minimal labeling of PFKL, consistent with its inability to inhibit NETosis (Figure S3B). While additional proteins emerged as potential targets of PALP1, cross referencing these results with the TPP experiment distinguished proteins that were stabilized by NA-11 from those that interacted directly with PALP1 with high affinity. Combining these two orthogonal approaches, PFKL was the only prominent protein with a high confidence based on combined quantitative changes, p values, and wide peptide coverage (Figures 2E and S3B). TBB4B was ruled an outlier based on a low score for interaction with PALP1 and inconsistent intensity across replicates (Figures S3A and S3C). Collectively, our analyses point to PFKL being the primary target of NA-11.

We validated the interaction of NA-11 with PFKL in THP-1 cells by immunoblotting PFKL in TPP samples (Figure 2F). NA-11, but neither vehicle nor inactive NA-4, caused a thermal shift in PFKL of 4°C, from 55°C ± 1°C to 59°C ± 1°C (Figure 2F). Although TPP highlighted NAMPT and phosphodiesterase PDE6δ as potential interactors of NA-11, they did not appear to contribute to NETosis inhibition. For example, NA-11 did not affect the activity of recombinant NAMPT (Figure S3D). Thus, NA-11 does not inhibit NETosis by directly modifying the enzymatic activity of NAMPT. PDE6δ sustains Ras signaling, which activates the Raf-MEK (mitogen-activated ERK kinase)-ERK (extracellular signal-regulated kinase) pathway (Chandra et al., 2011). NA-11 did not perturb PMA-induced activation of MEK or ERK in neutrophils (Figure S3E). Thus, NA-11 must not inhibit NETosis by compromising the MEK-ERK signaling cascade downstream of PDE6δ. Notably, neither the affinity pull-down nor TPP analyses identified GSDMD as a binding partner of NA-11 or PALP-1.

### NA-11 binds to the activating nucleotide effector site of PFKL

**Figure 3.**
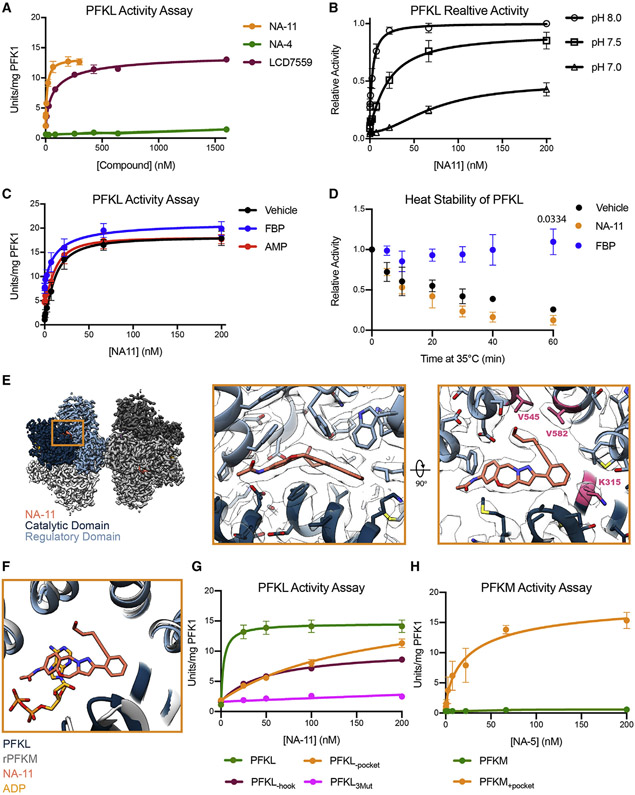
NA-11 binds at the AMP/ADP allosteric effector site to activate PFKL (A–D) Activity of recombinant PFKL. Data are the mean ± SEM of three independent experiments (A) or three measurements using two independent preparations of protein (B–D). In (D), p values at 1 h (paired Student’s t test) are shown when p < 0.05. (E) Cryo-EM structure of NA-11-bound PFKL. Regulatory and catalytic domains of one monomer are colored light and dark blue, respectively, with remaining monomers in gray. Higher magnifications of the NA-11 binding site are boxed in orange. V545, V582, and K315 are highlighted in pink. (F) Comparison of the orientation of NA-11 (orange) to ADP (yellow) in the nucleotide effector site of rabbit muscle PFK (PDB: 3O8N). (G and H) Activity of wild-type and mutant forms of PFKL (G) or PFKM (H). Data are the mean ± SEM of three measurements using two independent preparations of protein. Assay conditions in (A)–(C) and (G) are listed in Tables 1 and S1. See also Figures S4, S5, and S6.

To elucidate the mode of action of NA-11, we performed *in vitro* kinase assays with recombinant phosphofructokinase-1 (PFK1) enzymes. His-tagged human PFKL was allosterically regulated by the sugar substrate fructose 6-phosphate (F6P) and the nucleotide substrate and inhibitor ATP (Figures S4A-S4C; Table 1). Inhibition of PFKL by ATP (at a physiological concentration of 3.1 mM) was relieved by NA-11 with a half maximal effective concentration (EC_50_) of 14.00 ± 2.91 nM (mean ± SEM; Figure 3A; Table 1). Thus, NA-11 activates rather than inhibits PFKL. LDC7559 also activated PFKL, albeit at higher concentrations with an EC_50_ of 66.04 ± 18.93 nM. Thus, both NA-11 and LDC7559 engage PFKL. As predicted, the inactive analog NA-4 did not activate PFKL. Note that NA-11 and LDC7559 are the strongest, non-natural, small-molecule agonists of PFK1 with similar EC_50_ as the most potent natural activator of PFK1, fructose 2,6-bisphosphate (FDP) (Schöneberg et al., 2013).

We determined modulation of NA-11 activity by known allosteric effectors, namely pH, fructose 1,6-bisphosphate (FBP), and AMP. NA-11 activity to PFKL decreased with pH (Figure 3B; Table 1), suggesting that the protonation state of side chains in PFKL affects the ability of NA-11 to activate PFKL. All further assays for PFKL were performed at pH 7.5. The catalytic product FBP, which binds to the allosteric sugar effector site to stabilize tetrameric PFK1 and relieve ATP inhibition (Andrés et al., 1990; Foe et al., 1983; Hers and Van Schaftingen, 1982), did not alter the activity of NA-11 to PFKL but slightly increased the maximal velocity of reaction (Figure 3C; Table S1), suggesting an additive effect. AMP, which binds to a central nucleotide effector site in PFK1 and relieves ATP inhibition (Banaszak et al., 2011; Brüser et al., 2012), did not alter the activity of NA-11 for PFKL or the maximal velocity of reaction (Figure 3C; Table S1). Together, these data suggest that NA-11 binds to a site distinct from the allosteric sugar-binding site. In support of this prediction, FBP protected PFKL from thermal inactivation (Sánchez-Martínez and Aragón, 1997), but NA-11 did not (Figure 3D).

Cryoelectron microscopy (cryo-EM) of PFKL in the presence of ATP, F6P, and NA-11 revealed that PFKL primarily formed stacked pairs of tetramers (Figures S5A and S5B). These structures resembled a 25-Å negative-stain reconstruction of a PFKL filament (Webb et al., 2017). However, 3D classification revealed that one pair of tetramers was poorly resolved in most particles (Figures S5B and S5C), perhaps owing to damage at the air-water interface. This feature limited resolution of the NA-11-binding site. Therefore, a higher-resolution 2.9-Å structure of substrate- and NA-11-bound PFKL was determined by refinement focused on a single, masked tetramer (Figures 3E, 3F, S6A, and S6B) (PDB: 7LW1). Structures of the stacked tetramers and masked tetramer were in the same conformation (Cα root-mean-square deviation [RMSD] 0.65 Å). In the higher-resolution structure, ADP and F6P were clearly bound to the catalytic site, whereas the product and allosteric activator FBP was bound to the allosteric sugar-binding site (Figures S6C-S6F). NA-11 was bound at the AMP/ADP allosteric activation site in a different orientation from that of the nucleotides (Figure 3F), with the pyrazolo-oxazepine backbone of NA-11 bound to a pocket containing the aromatic residues F308, F537, Y578, and F670. We hypothesize the increased activity of NA-11 compared to LDC7559 results from improved interactions with PFKL. The acetamide nitrogen is in position to donate a hydrogen-bonding distance to D179 (3.1 Å), while the 4-pentynol substituent occupies a narrow channel with a terminal hydrogen-bonding interaction with Y578. The catalytic site of NA-11-bound PFKL was in the active, R-state conformation, as revealed by comparison to R-state bacterial PFK bound to F6P and ADP (PDB: 4PFK) (Figure S6E).

We tested if either LDC7559 or NA-11 could activate the other human PFK1 isoforms. Mammals have three *PFK1* genes, which encode the liver (PFKL), platelet (PFKP), and muscle (PFKM) isoforms. Most cell types express all three isoforms, but to varying degrees (Dunaway, 1983; Fernandes et al., 2020). The proteins are ~70% identical, with the differences imparting isoform-specific activity and regulation. Due to isoform-specific differences in F6P affinity and ATP inhibition, we optimized assays for each isoform to ensure >90% inhibition before performing titrations. Both LDC7559 and NA-11 failed to activate PFKM or PFKP at concentrations up to 100 μM (Figure S4D). We identified three residues in the NA-11-binding pocket that are unique to PFKL: K315, V545, and V582. Interestingly, mutation of these residues to those in the other isoforms in PFKL (K315R, V545L, and V582M), abbreviated as PFKL_3Mut_, increased the affinity for F6P from 1.97 ± 0.24 mM for wild-type PFKL to 0.57 ± 0.06 mM for PFKL_3Mut_ (Figure S4B; Table 1). To ensure similar activation in response to F6P, we performed ATP inhibition assays at an F6P concentration approximately twice the K_m,F6P_ values (Table 1). Under these conditions, both wild-type and mutant PFKL showed >80% inhibition at 3.1 mM ATP (Figure S4C). NA-11 had significantly reduced activity to PFKL_3Mut_, with no activation observed at 200 nM and only partial activation observed at 1 μM (Figures 3G and S4E; Table 1). These data confirm that K315, V545, or V582 in PFKL impart specificity for NA-11 activation.

The relative importance of the pyrazolo-oxazepine binding pocket and the 4-pentynol “hook” was determined using mutants PFKL(K315R) (named PFKL_−pocket_) and PFKL(V545L, V582M) (named PFKL_−hook_). Both mutants had decreased affinity for NA-11, with PFKL_−pocket_ having a more profound impact on NA-11 activation (Figure 3G; Table 1). Consistent with the significance of K315 in defining the binding pocket to allow binding of NA-11, the structure-activity relationship (SAR) analog NA-5, lacking the 4-pentynol (Figure S1E), had similar activity as NA-11 for wild-type PFKL (Table 1). In addition, NA-5 had comparable activity for PFKL_−hook_ as for wild-type PFKL, while the EC_50,NA-5_ of PFKL_−pocket_ was decreased ~30-fold (Figure S4F; Table 1).

We determined whether we could impart NA-5 sensitivity in PFKM by mutating the pocket arginine (R315) to lysine (PFKM_+pocket_) as in PFKL. PFKM_+pocket_ had a decreased maximal velocity of reaction and F6P affinity when compared with wild-type PFKM (Figure S4G). Therefore, we used twice the concentration of F6P for the mutant protein than we did for the wild-type enzyme to correct for the altered F6P affinity and ensured >90% inhibition at 4 mM ATP (Figure S4H). In contrast to the wild-type enzyme, PFKM_+pocket_ was activated by NA-5 with an EC_50_ of 10.94 ± 1.28 nM (Figure 3H, Table 1). Taken together, these data confirm that isoform-specific differences in the NA-11-binding site confer sensitivity to NA-11 activation.

### Activation of PFKL by NA-11 suppresses the oxidative burst

**Figure 4.**
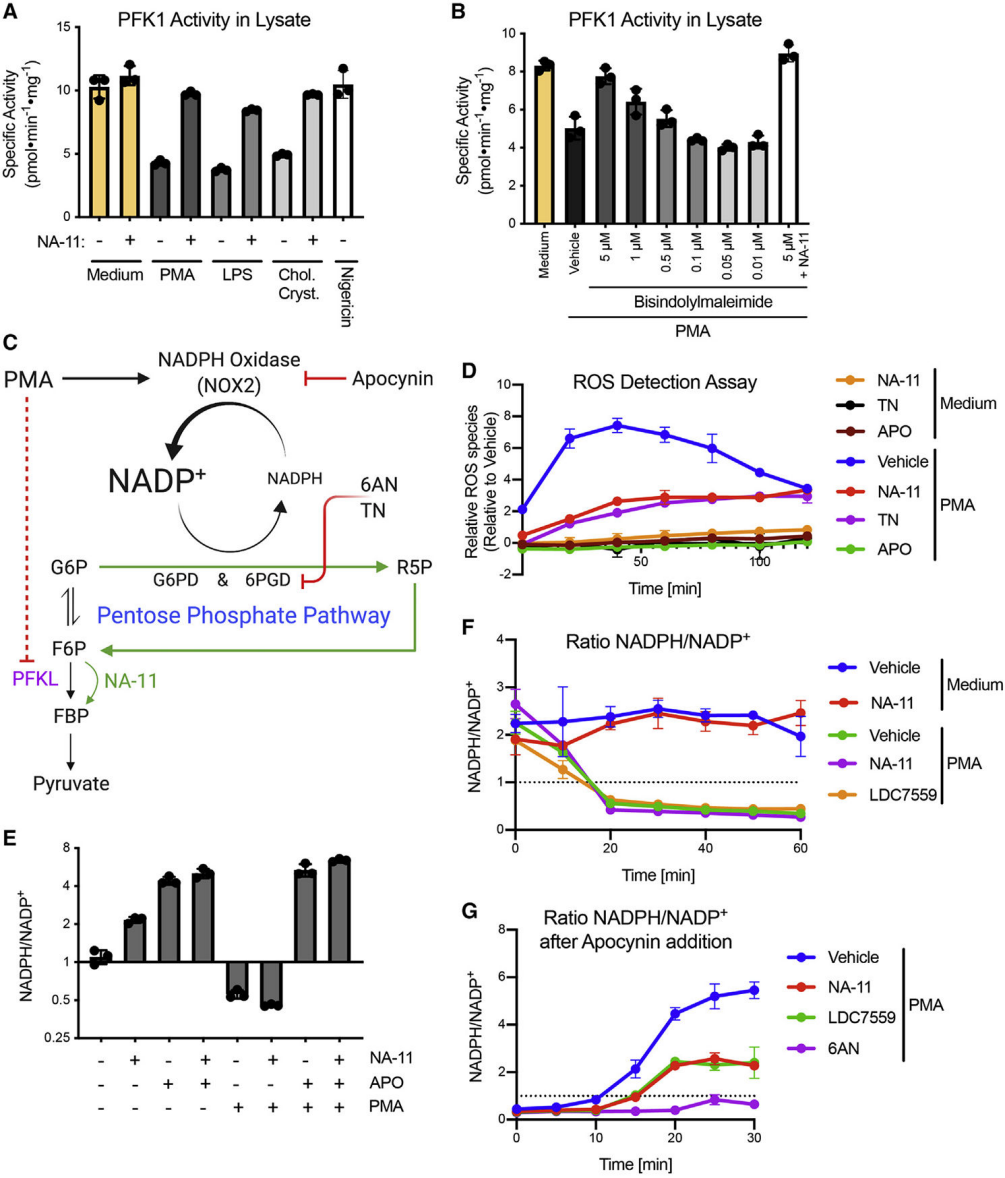
Reduced PFKL activity in NOX2-stimulated neutrophils is prevented by NA-11 (A and B) PFK1 activity in PMN lysates. Bars show the mean ± SD of PMNs from three donors. (C) Model for why activation of PFKL by NA-11 reduces NADPH availability for the oxidative burst. (D) ROS production by PMNs. Data are the mean ± SD of PMNs from three donors. (E–G) NADPH:NADP^+^ ratios in PMNs. Data are the mean ± SEM (E) or mean ± SD (F and G) of PMNs from three donors. In (G), apocynin was added 30 min after the treatments indicated, and ROS was measured over the course of 30 min to measure the rate of NADPH replenishment. See also Figure S7.

Although we cannot measure PFKL activity selectively in neutrophils, total PFK1 activity closely approximates the activity of PFKL, because *PFKL* is the dominant PFK1 isoform expressed in neutrophils (Fernandes et al., 2020). The effect of NA-11 on PFK1 activity in resting neutrophils was minimal, suggesting that PFK1 and the glycolysis pathway were already active (Figure 4A). Neutrophils treated with PMA, LPS, or cholesterol crystals showed less PFK1 activity when compared with resting neutrophils. This activation-induced reduction in PFK1 activity was prevented by NA-11 (Figure 4A) or LDC7559 (Figure S7A). Thus, NA-11 and LDC7559 may block NETosis by suppressing an activation-induced decrease in PFKL activity. Neutrophils activated with the NOX2-independent stimulus nigericin did not have lower PFK1 activity than resting neutrophils (Figure 4A), which fits with PFKL only modulating NOX2-dependent NETosis. NOX2-dependent NETosis requires activation of protein kinase C (PKC) (Hakkim et al., 2011). Treatment of neutrophils with the PKC inhibitor bisindolylmaleimide prior to activation with PMA prevented the decrease in PFKL activity, suggesting kinase-dependent PKC signaling inhibits PFKL (Figure 4B).

PFKL is a negative regulator of the phagocytic oxidative burst. Its knockdown in neutrophils shifts the glycolytic flux to the pentose phosphate pathway, resulting in increased intracellular NADPH for use by NOX2 (Graham et al., 2015). Accordingly, PFKL deficiency augments pathogen phagocytosis, increases ROS production, and enhances pathogen killing (Graham et al., 2015). Conversely, PFKL activation by NA-11 probably limits flux through the pentose phosphate pathway during NOX2-dependent NETosis (Figure 4C). Thus, cells are deprived of the NADPH required for the oxidative burst. To test our model, we monitored neutrophils for PMA-induced ROS. Production of ROS, which peaked after 20–40 min, was NOX2 dependent, because it was prevented by apocynin, an inhibitor of NADPH oxidase (Figure 4D). NA-11 markedly suppressed, but did not completely prevent, PMA-induced ROS. Thus, NA-11 may limit the oxidative burst without interfering with regular cellular redox activity (Figure 4D). Neutrophils treated with NA-11 resembled those treated with thionicotinamide (TN), a pseudosubstrate of NAD kinase and G6PDH, which are the main enzymes responsible for NADPH biosynthesis (Figure 4D).

We examined cellular levels of NADPH during neutrophil activation directly by measuring the ratio of NADPH/NADP^+^. PMA triggered a dramatic reduction in the NADPH/NADP^+^ ratio, reaching a new steady state within 30 min (Figures 4E and 4F). The ratio of NADH/NAD^+^ remained largely unchanged under these conditions (Figure S7B), suggesting that conversion of NADH to NADPH does not contribute significantly to the NADPH pool. Therefore, NADPH turnover through the pentose phosphate pathway is rate limiting to ROS production by activated neutrophils. Pretreatment of neutrophils with apocynin to inhibit NOX2 resulted in a high ratio of NADPH/NADP^+^ that was roughly twice that of untreated cells (Figure 4E). Importantly, NA-11 treatment of resting cells did not change the NADPH/NADP^+^ ratio, reinforcing the notion that NA-11 does not interfere with regular cellular redox activity (Figure 4F).

We also evaluated the effect of NA-11 on the rate of NADPH turnover through the pentose phosphate pathway. When apocynin was used to inhibit NOX2 in neutrophils that had been activated with PMA for 30 min, the cells quickly replenished NADPH and achieved a high NADPH/NADP^+^ ratio (Figure 4G). If NA-11 was present throughout the experiment, then the neutrophils were slower to replenish NADPH and ended up having a low NADPH/NADP^+^ ratio that approximated that observed in resting neutrophils (Figure 4G). Inhibition of G6PDH with 6-aminonicotinamide (6-AN) effectively blocked NADPH turnover, emphasizing the role of the pentose phosphate pathway in supplying NADPH to maintain the oxidative burst.

### NA-11 prevents a PMA-induced shift in glycolytic flux

**Figure 5.**
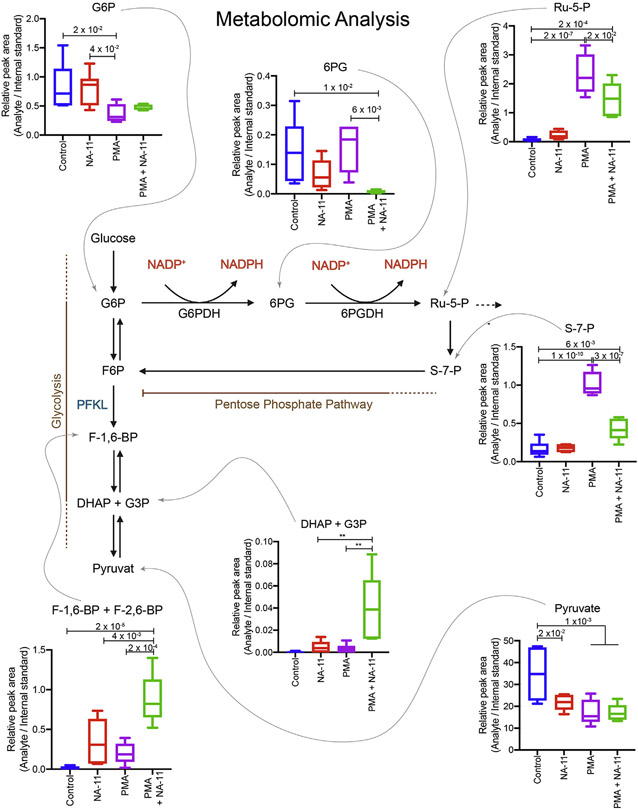
NA-11 prevents a switch in glycolytic flux in activated neutrophils Relative abundance of glycolysis and pentose phosphate pathway metabolites in PMNs treated with NA-11 or DMSO vehicle for 30 min and then PMA for 30 min. Box and whisker plots show the mean ± minimum and maximum of PMNs from six donors. p values (one-way ANOVA, multiple comparisons) are shown when p < 0.05.

Increased flux through the pentose phosphate pathway should alter the abundance of metabolites produced by the pathway. By mass spectrometry, PMA-activated neutrophils showed a significant decrease in glycolysis metabolites, including glucose-6-phosphate (G-6-P) and pyruvate, whereas pentose phosphate pathway metabolites, including 6-phosphogluconate (6-PG), ribulose-5-phosphate (Ru-5-P), and sedoheptulose-7-phosphate (S-7-P), were increased (Figure 5). Exposure to NA-11 alone slightly increased levels of FBP, which is the direct product of PFKL, but this did not translate into increased amounts of glycolysis products such as pyruvate. In the context of PMA activation, however, NA-11 maintained pentose phosphate pathway metabolites at baseline levels and increased glycolysis metabolites such as FBP, dihydroxyacetone phosphate (DHAP), and glyceraldehyde-3-phosphate (G-3-P). Thus, PFKL serves as a regulatory node for the oxidative burst. By maintaining the activity of PFKL, NA-11 limits flux through the pentose phosphate pathway and thereby deprives the cell of the NADPH needed for an effective oxidative burst.

### NA-11 inhibits the oxidative burst during phagocytosis and impairs bacterial killing

**Figure 6.**
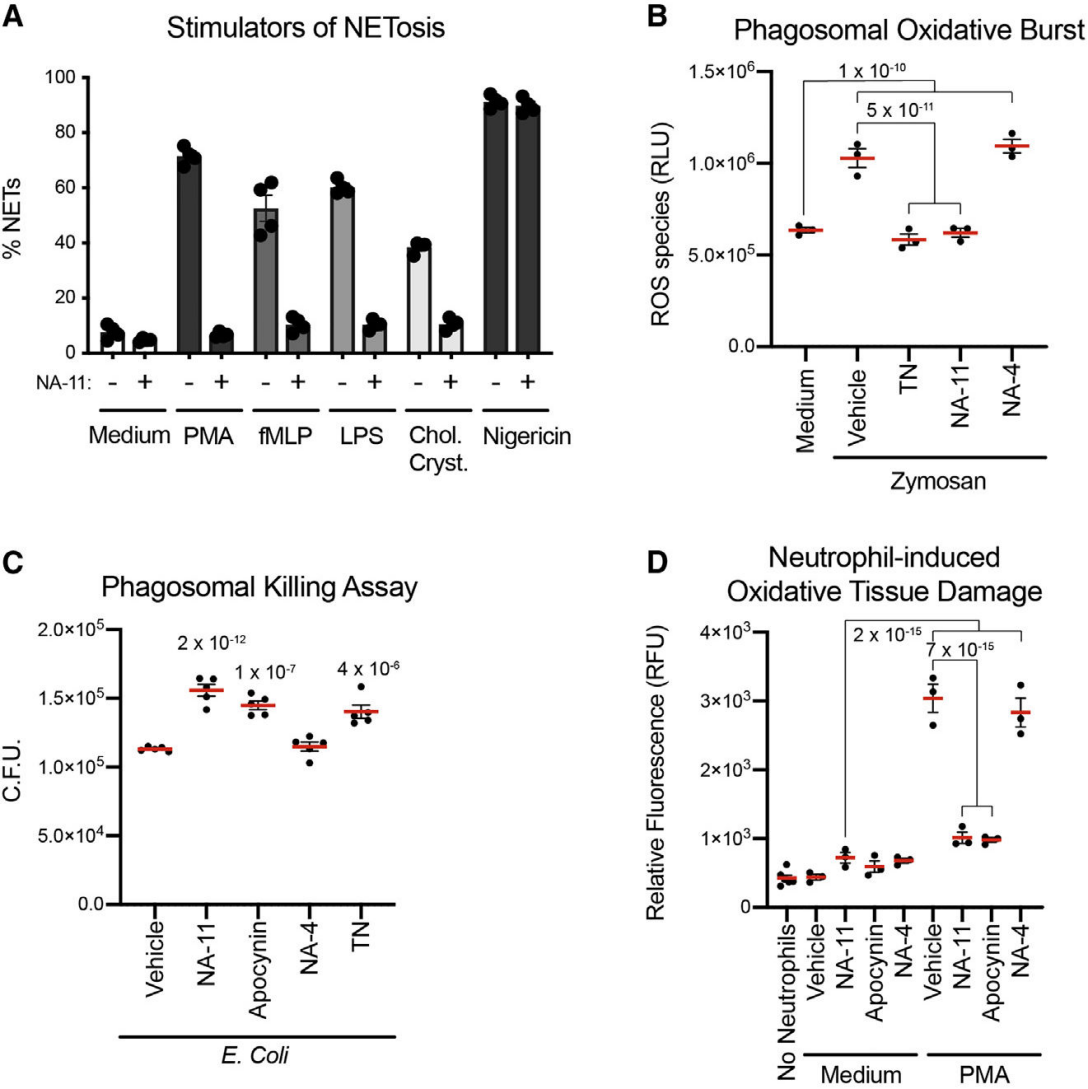
NA-11 impairs neutrophil defenses and prevents tissue damage (A) Percentage of PMNs undergoing NETosis. LPS indicates extracellular LPS. Bars show the mean ± SEM of PMNs from four donors. (B) ROS produced by PMNs during zymosan-induced phagocytosis. Data are the mean ± SEM of PMNs from three donors. p values (two-way ANOVA, multiple comparisons) are shown when p < 0.05. (C) *E. coli* colony-forming units (C.F.U.) recovered from PMNs after bacterial phagocytosis. Bars show the mean ± SEM of PMNs from five donors. p values (two-way ANOVA, means compared to vehicle) are shown when p < 0.05. (D) Graph indicates the amount of FITC-dextran to migrate through a monolayer of HBECs in the presence or absence of PMNs treated as indicated. RFU, relative fluorescence units. Data are the mean ± SEM of PMNs from three donors. p values (two-way ANOVA, multiple comparisons) are shown when p < 0.05. See also Figure S7.

Consistent with NA-11 suppressing the oxidative burst, it inhibited NOX2-dependent NETosis induced by the chemotactic peptide N-formyl-met-leu-phe (fMLP), LPS, and cholesterol crystals (Figures 6A and S7C). It also inhibited NOX2-dependent ROS production during phagocytosis (Figure 6B). Neutrophils cultured with opsonized zymosan particles nearly doubled their ROS production over 2 h, but pretreatment with either NA-11 or the pentose phosphate pathway inhibitor TN kept ROS production at basal levels. In contrast, inactive analog NA-4 did not suppress ROS production (Figure 6B). Importantly, neither NA-11 nor TN affected phagocytosis, because control and inhibitor-treated neutrophils contained comparable numbers of intracellular zymosan particles (Figure S7D).

Impaired ROS production by NA-11 should compromise neutrophil killing of phagocytosed bacteria. To test this possibility, intracellular bacteria were enumerated following phagocytosis. Neutrophils pretreated with NA-11, but not NA-4, produced more bacterial colonies than control neutrophils (Figure 6C). Thus, NA-11 makes neutrophils less efficient killers of phagocytosed bacteria, similar to apocynin or TN (Figure 6C).

### NA-11 prevents neutrophil-induced damage to an epithelial barrier

Neutrophil-mediated ROS and NETosis contribute to lung injury in inflammatory syndromes such as acute respiratory distress syndrome (ARDS) (Jasper et al., 2019; Yang et al., 2020). We modeled neutrophil-induced tissue damage in cell culture by layering neutrophils on top of human bronchial epithelial cells (HBECs) in a transwell plate (Figure 6D). When the neutrophils were activated with PMA, they damaged the epithelial layer, which allowed cell-impermeable dextran to migrate from the cultures into the receiver plate. The amount of dextran detected in the receiver plate increased as more neutrophils were added to the epithelial layer (Figure S7E). Both NA-11 and apocynin protected the epithelial layer from this neutrophil-induced oxidative damage, whereas NA-4 did not (Figure 6D). Thus, NA-11 inhibits NOX2-dependent NETosis, ROS production during phagocytosis, and oxidative damage to healthy bystander cells.

## DISCUSSION

The phagocyte oxidative burst is essential for host defense because it generates bactericidal ROS and promotes NETosis. Excessive NET formation is implicated in a wide range of pathologies, including chronic obstructive pulmonary disease (COPD), ARDS, and asthma (Jasper et al., 2019; Vorobjeva and Chernyak, 2020; Yang et al., 2020). Markers of NETosis are elevated in the serum of patients infected with SARS-CoV-2 (Zuo et al., 2020), and serum from coronavirus disease 2019 (COVID-19) patients can induce NETosis in the blood of healthy donors (Yoshida et al., 2020). Excessive NETosis induces immunothrombosis and correlates with the severity of ischemic stroke and myocardial infarction (Zucoloto and Jenne, 2019). NETosis activated by crystalline particulates contributes to the pathogenesis of atherosclerosis and pancreatitis (Leppkes et al., 2016; Warnatsch et al., 2015). Finally, components of NETs may stimulate production of antibodies and development of autoimmune diseases (Fousert et al., 2020).

Potential approaches to alleviate NETosis include anti-cytokine therapy to prevent neutrophil accumulation, clearance of NETs by exogenous nucleases, and inhibitors that target NE, PAD4, NOX2, or G6PDH in the NETosis pathway (Diebold et al., 2015; Kowalik et al., 2017; Papayannopoulos et al., 2011; Vorobjeva and Chernyak, 2020). While inhibition of the oxidative burst prevents NETosis, complete inhibition of NOX2 and ROS production is undesirable, because ROS regulate major pathways in immunity. Type 1 interferon signaling, autophagy, and the production of inflammatory cytokines and chemokines are modulated by ROS (Thomas, 2017). Inhibitors of the pentose phosphate pathway exhibit low selectivity and general toxicity (Ghergurovich et al., 2020; Kowalik et al., 2017). LDC7559 and NA-11 represent a family of compounds based on a pyrazolo-oxazepine scaffold that suppress the oxidative burst without compromising basal ROS by limiting the availability of NADPH. This inhibitory function is achieved through selective activation of PFKL, which prevents activated neutrophils from fully engaging the pentose phosphate pathway.

Here, we used a combination of two orthogonal chemical proteomic methods to identify the target of LDC7559 that is relevant for its pharmacologic phenotype in neutrophils. While combining experimental strategies is increasingly popular (Ha et al., 2021; Wilkinson et al., 2020), the standard approach to target identification involves a crude proteome-wide analysis initially, followed by target validation for select hits. Utilizing two proteome-wide, unbiased orthogonal methods is rare. We demonstrate that a multiplexed analysis of PAL with global TPP provides a robust platform for target identification and validation.

The structure of human PFKL bound with NA-11 revealed the activating nucleotide effector site as its bona fide binding site and shed light on its mode of action. PFK1 isoform selectivity is due to three residues unique to PFKL: K315, V545, and V582. NA-11 binding in the nucleotide effector site mimics allosteric activation by AMP or ADP, inducing the active, R-state conformation. NA-11 had improved efficacy in cells when compared to LDC7559, with a 1,000-fold decrease in IC_50_ for the inhibition of NETosis. In contrast, *in vitro* analysis with recombinant PFKL showed only a moderate difference in PFKL agonism by NA-11 and LDC7559, with EC_50_ values of 14.00 ± 2.91 nM and 66.04 ± 18.93 nM, respectively. The discrepancy may reflect differences in the intrinsic stability and cell permeability of the two compounds.

Our study highlights the importance of the acetamide group in NA-11 for agonism of PFKL. Of note, Sollberger et al. modified the acetamide group of LDC7559 to make compound LDC2618 for immobilization and target pull-down. It is unclear, however, if LDC2618 blocked NETosis (Sollberger et al., 2018). Thus, we hypothesize that PFKL was not identified as a target of LDC7559 previously, because the modification in LDC2618 prevented binding to PFKL.

LDC7559 and its more potent analog, NA-11, are non-natural agonists of PFKL, with activities that are comparable to the most potent natural allosteric activator, FDP. Pharmacologic activators of glycolysis are scarce and may have pharmacologic potential. For example, activators of pyruvate kinase (PKM2), the rate-limiting enzyme catalyzing the last step of glycolysis, have been shown to reduce the ROS-detoxification capacity of cancer cells and suppress tumor growth (Shahruzaman et al., 2018). Activation of PKM2 has also been shown to limit progression of diabetic glomerular pathology and mitochondrial dysfunction (Qi et al., 2017). Isoform-specific differences in binding pocket topology, together with accessibility to the pyrazolo-oxazepine scaffold, establish the possibility of generating isoform-specific activators of PFK1. These tools will be a valuable resource to determine isoform-specific functions of PFK1 in glucose metabolism and their dysregulation in diseases. At the level of transcription, the dominant isoform of *PFK1* expressed in most human cells is *PFKP,* although *PFKM* dominates in muscle cells, and *PFKL* is the dominant isoform in immune cells (Fernandes et al., 2020). It is unclear if PFK1 protein levels, which are also regulated post-translationally (Feng et al., 2020; Park et al., 2020), follow this pattern. Isoform selectivity likely confers specificity toward cells of the immune system.

The role of metabolic pathways in the oxidative burst was first noted in the context of phagocytosis (Zatti and Rossi, 1965). A sharp increase in oxygen consumption and glucose uptake increases NADPH levels through the pentose phosphate pathway. Subsequently, it was found that PFK1 regulates the oxidative burst during phagocytosis. In macrophages, knockdown of PFK1 increases ROS production in response to zymosan (Graham et al., 2015). The suppression of PFK1 and increased NADPH generation was also shown to potentiate cancer cell survival under metabolic stress (Kim et al., 2017). We now show the impact of PFK1 activity in neutrophils, where it suppressed the NOX2-dependent oxidative burst. PMA, LPS, or cholesterol crystals reduced PFK1 activity in neutrophils to promote NOX2-dependent NETosis, except when the cells were treated with NA-11 or LDC7559. In contrast, NETosis induced in a NOX2-independent manner by the ionophore nigericin did not change PFK1 activity and was not blocked by NA-11.

The activity of PFKL is suppressed by the kinase PKC, but precisely how PKC limits PFKL activity is unclear. PKC may regulate PFK1 indirectly through FDP, which is a potent activator of PFK1 and an important modulator of glycolysis. For example, PKC is known to phosphorylate some of the 6-phosphofructo-2-kinase/fructose-2,6-bisphosphatase (PFKFB) enzymes that regulate the abundance of FDP (Rider et al., 1992; Dasgupta et al., 2018; Sakakibara et al., 1997). It is unclear, however, which of the PFKFBs is dominant in neutrophils.

Identification of NA-11 as a suppressor of the phagocyte oxidative burst raises the possibility that it may have therapeutic benefit in neutrophil-driven diseases. Neutrophils are rarely considered druggable cells because they are key players in innate immunity, but inhibition of induced rather than basal activation of NOX2 may provide an intervention opportunity. The molecular understanding of PFK isoform selectivity, coupled with future studies of the pyrazolo-oxazepine scaffold, could open the door to selective activation of PFKM and PFKP, and the development of new pharmacological therapies for diabetes and cancer.

### Limitations of the study

The selectivity for binding and activation of PFKL by NA-11 is an important aspect of this work. We show that NA-11 agonizes PFKL homotetramers, but it is unclear if it would agonize heterotetramers composed of different isoform combinations. Given that all three PFK1 isoforms are expressed at different ratios in different cells, it is hard to predict the extent to which NA-11 will affect overall PFK1 activity. Besides neutrophils, T cells and hepatocytes also express PFKL, but the activity of NA-11 on these cells remains to be evaluated. Finally, the phenotypes and cellular processes characterized in this study are related to human neutrophils. The activity of NA-11 toward PFK1 from other species needs to be determined before a preclinical model can be selected to evaluate the pharmacologic efficacy of NA-11.

## STAR★METHODS

### RESOURCE AVAILABILITY

#### Lead contact

Further information should be requested from the Lead Contact, Vishva M. Dixit (dixit@gene.com, 1-650-438-7064).

#### Materials availability

Details on the synthesis of the new chemical entities described in this study are provided in the supplemental information.

#### Data and code availability

CryoEM data have been deposited at PDB and EMDB. Mass spectrometry raw data have been deposited in the MassIVE database (https://massive.ucsd.edu/ProteoSAFe/static/massive.jsp). Accession numbers are listed in the key resources table.

### EXPERIMENTAL MODEL AND SUBJECT DETAILS

#### Human blood samples

Peripheral blood samples from healthy male and female donors at least 18 years of age were kindly provided by the Samples4S-cience donor program at Genentech. Donors provided written informed consent and sample collection was approved by the Western Institutional Review Board.

#### Human cell lines

THP-1 cells were cultured in RPMI 1640 medium supplemented with 5% fetal bovine serum (FBS) and 2 mM L-glutamine. Human bronchial epithelial cell (HBEC) line UNCN1T was cultured in complete PneumaCult culture medium supplemented with 10 μM ROCK inhibitor (Y-27632). Cells were passaged at a 1:20 dilution every 2-4 days.

### METHOD DETAILS

#### Isolation of human peripheral blood neutrophils and monocytes

Blood (25 mL) was layered on top of a biphasic Histopaque gradient (25 mL) and centrifuged at 700 x g for 30 min with acceleration and deceleration ramps set to lowest. The top layer of PBMCs was collected and monocytes were purified with EasySep isolation kit (StemCell Tech.) using immunomagnetic negative selection. Neutrophils were recovered from the interface between the Histopaque 1077 and 1119 layers with a wide mouth Pasteur pipette and diluted to 50 mL with D-PBS (without calcium and magnesium). Cells were pelleted by centrifugation at 300 x g for 5 min. Red blood cells were lysed by resuspending pellets in 5 mL of 0.2% NaCl for 30 s, and then 5 mL of 1.6% NaCl was added. Cells were pelleted again by centrifugation (300 x g for 3 min), washed twice with D-PBS, and resuspended in warm RPMI 1640 medium supplemented with 2 mM L-Glutamine and 10 mM HEPES. Neutrophil purity was assessed by Giemsa staining to be > 95%.

#### Stimulation of neutrophils for NETosis and immunofluorescence assays

Freshly purified neutrophils were plated at 6 × 10^3^ cells/well in glass bottom 96-well plates (SensoPlate, Greiner Bio-One 82050-792) in the RPMI medium described above. Cells were pretreated with DMSO vehicle, 0.5 μM NA-11, 100 μM apocynin, 0.5 μM and 5 μM PALP, or 0.5 μM and 5 μM LDC7559 for 30 min, and then stimulated with 50 nM PMA, 1 μM f-MLP, 5 μg/mL cholesterol crystals, 10 μg/mL LPS, 25 μM nigericin, or 25 μM calcymicin (A23187) for 3 h (except in Figures S1A, 6A and S7C, where cells were stimulated for 4 h). Cells were fixed and permeabilized in 2.5% paraformaldehyde (PFA) and 0.02% Triton X-100 in PBS for 30 min. For quantification of NETosis, cells were stained with 150 nM SYTOX Green and imaged in a KEYENCE Fluorescence Microscope (KEYENCE, ZB-X800E) at 10 x magnification. For immunolabeling, fixed cells were blocked with 2% BSA for 1 h, and then stained with anti-CitH3 and anti-MPO antibodies at a dilution of 1/200 in 1% BSA in PBS. Images were taken at 60 x magnification using an ImageXpress Micro Confocal high-content imaging system (Molecular Devices).

#### Quantification of NETosis using SYTOX Green

Semi-automated analysis of both phase and fluorescence images of SYTOX Green-stained neutrophils used a specialized MATLAB algorithm. Total cells were counted by scoring phase standard deviation and radial symmetry. NETosis was classified in cells by measuring the DNA area in the fluorescence image and comparing with a select internal training set for each experiment. Unstimulated cells and PMA-stimulated, pyocyanin-inhibited cells served as negative controls. PMA-stimulated cells served as a positive control. NET-forming cells are presented as a percentage of total cells.

#### GSDMD cleavage assays

1 μg recombinant GSDMD (Aglietti et al., 2016) was incubated with 10 μM LDC7559, 30 μM zVAD-fmk, or DMSO vehicle for 20 min, and then combined with 0.15 μg recombinant caspase-4 (750 mU; Abcam) in 20 μL of 50 mM HEPES, pH 7.2, 50 mM NaCl, 0.1% Chaps, 10 mM EDTA and 5% glycerol for 30 min at 37°C.

#### TR-FRET assay

GSDMD-dependent liposome cargo release was conducted by Confluence Discovery Technologies with modifications to the procedure described previously (Aglietti et al., 2016). Briefly, liposomes with an encapsulated cargo of europium-chelate conjugated biotin were formed from a lipid mixture of DOPC:DOPE (1:1). 0.15 mg/mL of cargo-loaded liposomes were mixed with 0.3 μM of recombinant GSDMD and incubated with 10 μM LDC7559, 10 μM disulfiram or DMSO vehicle. 0.15 μM caspase-4 and 50 nM streptavidin-Alexa Fluor 647 were added and TR-FRET was read over 1 h using a PHERAstar FSX Microplate Reader (BMG Labtech) equipped with a TR-FRET module.

#### Inflammasome assays

Primary human monocytes were primed with 1 μg/mL ultrapure LPS and 1μg/mL Pam3CSK4 for 3 h. THP-1 cells were primed with 1 μg/mL Pam3CSK4 for 4 h. Primed cells (1 × 10^5^) were treated for 1 h with DMSO vehicle, 5 μM LDC7559 (unless indicated otherwise), 10 μM MCC950, or 50 μM VX765, and then for 1 h with 10 μg/mL nigericin, or 25 μg/mL LPS (electroporated using the NEON electroporation system; ThermoFisher Scientific), or they were transfected over 3 h with 1 μg/mL poly(dA-dT) using lipofectamine 2000 (0.5 μL/well).

LDH released into the culture medium was measured 1-3 h post-activation using a CellTox Green Cytotoxicity assay (Promega). IL-1β secretion was measured using a MSD Cytokine Assay, 96-well multi-array Tissue Culture Kit (Meso Scale Discovery). For immunoblotting, monocytes (2 × 10^6^) were treated with 10-50 μM LCD7559 or 50 μM VX765 and electroporated with 25 μg/mL LPS using the NEON electroporation system. Cells were lysed after 1 h using lysis buffer (50 mM Tris pH 7.4, 150 mM NaCl, 10% glycerol, 1% NP-40, 1 mM MgCl and 1X Roche cOmplete protease inhibitor cocktail) and protein concentration was determined via BCA protein assay (Peirce). Antibodies for immunoblotting were GSDMD (L60, Cell Signaling Technologies), cleaved GSDMD-Asp275 (E7H9G, Cell Signaling Technologies) and GAPDH (14C10, Cell Signaling Technologies).

#### NE activity assay

NA-11 and LDC7559 were incubated at the indicated concentrations with 25 ng of recombinant NE for 20 min. NE activity was measured using a fluorescence NE activity assay kit (Abcam).

#### NAMPT activity assay

NA-11 was diluted in ultra-pure water at concentrations between 500 nM to 160 pM, and preincubated with recombinant NAMPT for 30 min. NAMPT activity was measured using a fluorescence NAMPT Inhibitor Screening assay kit (BPS Bioscience).

#### Chemical synthesis

Compounds and photoreactive probes were synthesized at Wuxi Apptech Inc. See Methods S1 in the supplementary information for synthesis procedures and chemical characterizations.

#### Preparation of cholesterol crystals

Cholesterol crystals were prepared as described (Warnatsch et al., 2015). Briefly, 12.5 mg cholesterol (Sigma-Aldrich) was solubilized in 1 mL of 95% ethanol at 65°C. Crystals were formed by 5 consecutive freeze/thaw cycles, collected by centrifugation resuspended in D-PBS.

#### Thermal protein profiling (TPP)

Neutrophils (3 × 10^7^) were treated with 50 nM PMA for 20 min. Cell were washed twice in D-PBS, resuspended in KB buffer (25 mM Tris-HCL pH 7.5, 1 mM DTT, 10 mM MgCl_2_) and frozen in liquid nitrogen. Lysates were prepared by 5-7 freeze-thaw cycles in KB buffer. Protein concentration was determined via Bradford assay and lysates were incubated on ice for 30 min with either 10 μM NA-11 or DMSO vehicle. Samples were analyzed in two formats: (1) classical thermal proteome profiling (TPP) with full melting curves using 16-plex TMT labeling (8 temperatures between 37-65°C), and (2) Proteome Integral Solubility Alteration (PISA) assay with aggregated protein abundance from a defined temperature range using 10-plex TMT labeling (Five replicates 44-58°C). Aliquots (5 mg) were divided and heated at 8 temperatures in the 37-65°C range or 6 temperatures in the 44-58°C range, and cooled on ice for 3 min.

For TPP, heated lysates from different temperatures were kept separate. For PISA, lysates from different temperatures were combined based on treatment. Samples were centrifuged at 20,000 x g for 20 min at 4°C and supernatants were isolated for analysis. Proteins were denatured in 4 M urea, reduced with 5 mM DTT at 60°C for 20 min, and alkylated with 11 mM iodoacetamide (IAA) for 20 min. Urea was diluted to 2 M with 20 mM HEPES. Proteins were digested with Lys-C (Wako) diluted 1:50 for 3 h at 37°C, and then with Trypsin (PTMScan) diluted 1:100 overnight at 37°C. Peptides were cleaned on C18 Sep-Pak cartridges, 50 mg absorbent (Waters, WAT054960) and labeled with 16-plex TMTpro or 10-plex TMT. Labeled peptides were pooled, cleaned on a C18 Sep-Pak cartridge, and fractionated by offline high pH reversed phase separation and combined into 12 fractions in checkerboard fashion. Peptides were cleared on C18 STAGE-tips before mass spectrometry.

#### LC-MS analysis of metabolites of glycolysis and the pentose phosphate pathway

Neutrophils (1 × 10^7^) were untreated or pretreated with 0.5 μM NA-11 for 25 min, and then activated with 50 nM PMA for 30 min. Cell were washed twice in D-PBS and frozen in liquid nitrogen. Metabolites were extracted following protein precipitation using 500 μL cold (−80°C) MeOH:H_2_O (8:2, V:V) mixture. Aliquots (250 μL) were evaporated and reconstituted with 75 μL of MeOH:H2O (8:2, V:V) containing a series of stable isotope labeled internal standards. LC-MS analysis was performed on a Shimadzu Nexera HPLC series system (Shimadzu, Kyoto, Japan) couple with a Thermo Q Exactive Plus Hybrid Quadrupole-Orbitrap Mass Spectrometer (Thermo Fisher Scientific, Waltham, MA, USA). A Phenomenex Luna NH2 HPLC column (100 Å, 250 mm × 2 mm × 3 μm, Phenomenex, Torrance, CA, USA) was used to separate the analytes of interest. Samples (5 μL) were analyzed under Heated Electrospray Ionization (HESI) in negative mode with a flow rate of 0.4 mL/min and a total run time of 50 min. The mobile phases consisted of (A) 20 mM ammonium hydroxide and 20 mM ammonium acetate in H_2_O and (B) acetonitrile. The gradient was 0 min, 85% B; 0.5 min, 85% B; 15 min, 0% B; 38 min, 0% B; 40 min, 85% B; 50 min, 85% B. The autosampler temperature was 4°C, and the column temperature was 40°C. The Q Exactive Plus Mass Spectrometer parameters were: sheath gas flow rate, 50 units; aux gas flow rate, 13 units; aux gas temperature, 425°C; capillary temperature, 263°C; spray voltage, −2500 V; scan mode, full MS; scan range, 60-900 m/z; resolution, 70,000; AGC target, 3 × 10^6^; maximum IT, 200 ms. Data was processed with TraceFinder (Thermo Fisher Scientific, Waltham, MA, USA). %RSDs of a series of stable isotope-labeled internal standards were calculated to check system suitability and data robustness. Relative MS peak area ratio (Peak Area of Analyte / Peak Area of Internal Standard) was used for compound quantification. For LC-MS/MS analysis of pyruvate, sample derivatization procedures and instrument parameters were similar to that described previously (Daemen et al., 2018). Pyruvate concentration was quantified using a calibration curve of chemical standard (with stable isotope labeled internal standard, Pyruvate-^13^C3) in the surrogate matrix.

#### Photoreactive-probe pull-down with DADPS biotin azide linker

Buffy coats (100 mL) were diluted with ice-cold water (540 mL) for 30 s to lyse red blood cells. Following addition of 10 x HBSS buffer (50 mL) leukocytes were pelleted for 3 min at 300 x g, then washed twice in D-PBS and resuspended in RPMI medium. Cells (2 × 10^7^) were plated on 15-cm dishes in DMSO vehicle, 1 μM PALP1 or 1 μM PALP6 for 30 min at 37°C. For competition, 10 μM NA-11 was added for 15 min, and then 1 μM PALP1 was added for an additional 30 min. To crosslink the photoaffinity probe, cells were UV-irradiated (High intensity mercury spot lamp, 100W, 365 nm, UVP) in RPMI for 5 min on ice. Cells were lysed in 200 μL 10 mM Tris pH 7.5, 150 mM NaCl, 1% NP-40, 2.5 mM MgCl_2_, 0.5 mM CaCl_2_, 2% glycerol, 10 μg/mL DNase-I, and 1 x EDTA-free Protease inhibitor cocktail. Lysates from 5 plates for each condition were pooled and incubate 10 min on ice.

Biotin-DADPS tagging, biotin-streptavidin affinity purification, and on-bead digestion were performed as described (Hewings et al., 2018). Briefly, lysates were cleared by centrifugation and the lysate concentration adjusted to 5 mg/mL. Streptavidin Ultralink Resin (100 μL of 50% slurry, Pierce) was washed twice with PBS and mixed with 1 mL of lysate for 30 min at room temperature. Captured proteins were washed twice with PBS, and then the acid-cleavable DADPS Biotin Azide linker (Click Chemistry Tools, Cat# 1330) was added via Cu-catalyzed azide-alkyne cycloaddition. The following reagents were added sequentially with vortexing between each addition: 100 μM Biotin DADPS Azide (10 μL of a 10 mM stock solution made in DMSO), 2 mM 2-(4-((bis((1-(tert-butyl)-1H-1,2,3-triazol-4-yl)methyl)amino)methyl)-1H-1,2,3-triazol-1-yl)acetic acid (BTTAA, 20 μL of a 100 mM stock solution made in H_2_O), 2 mM CuSO_4_ (50 μL of 40 mM stock solution made in H_2_O) and 4 mM sodium ascorbate (40 μL of a 100 mM freshly prepared solution made in H_2_O). Tubes were rotated at room temperature for 1 h. Reactions were stopped with 5 mM EDTA (20 μL of a 250 mM stock solution). Proteins were precipitated with ice-cold methanol (3 mL), followed by the addition of chloroform (750 μL) and water (2 mL). Tubes were vortexed and then centrifuged at 3000 x g for 10 min. Precipitates were washed twice with methanol, air-dry for 5 min, and then solubilized in 0.2 mL of PBS containing 2% sodium dodecyl sulfate (SDS) and 6 M urea, with sonication (30% power, 5 s bursts). PBS was then added to dilute the SDS to ~0.25%. Labeled proteins were captured with PBS-washed Streptavidin Ultralink Resin (200 μL of 50% slurry) at 4°C for 24 h. Beads were then washed in 3 × 1 mL of the following solutions, with a 5 min incubation for the first wash in each solution: (i) 2% SDS, 10 mM EDTA in PBS; (ii) 1 M NaCl, 0.2% NP-40 in PBS; (iii) 8 M urea in PBS; (iv) PBS. Washed beads were resuspended in 0.5 mL 6 M urea in PBS. 10 mM tris(2-carboxyethyl)phosphine (TCEP, 10 μL of a 500 mM solution, pH 7) was added and tubes were rotated at 37°C for 30 min. 25 mM IAA (25 μL of a 500 mM solution) was added and the tubes were incubated for 30 min at room temperature in the dark. Beads were washed twice with PBS, twice with 50 mM Tris pH 8.0, and then resuspended in 67 μL 6 M urea in 50 mM Tris pH 8.0.

On bead digestion was performed with 2 μL of 2 mg/mL Trypsin/Lys-C Mix, Mass Spec Grade (Promega) for 3 h at 37° C. 323 μL of 50 mM Tris pH 8.0 was added and the tubes were rotated at 37°C overnight. Peptides were recovered by brief centrifugation. Further material was eluted from the beads with 100 μL 1 M urea in 50 mM Tris pH 8.0, and then 100 μL water. Combined eluates were desalted on C18 STAGE-tips and labeled with 0.16 mg 11-plex TMT. After labeling, peptides were pooled and cleaned again on a C18 STAGE-tip. DADPS linker cleavage was performed in 2% formic acid in water.

#### LC-MS/MS analysis

Data was collected on an Orbitrap Fusion Lumos Tribrid Mass Spectrometer (Thermo Fisher Scientific). Peptides were loaded onto a 25 cm IonOpticks Aurora Series column (IonOpticks) in Solvent A (98% water, 2% acetonitrile, 0.1% formic acid) via a Nanospray Flex Ion-Source (Thermo Scientific) with a flow rate of 0.5 μL/min in Solvent A (98% water/2% acetonitrile/0.1% formic acid). Separation occurred using a Thermo UltiMate 3000 RSLCnano ProFlow (Thermo Fisher Scientific) at a flow rate of 0.3 μL/min with a linear gradient of 2 to 35% solvent B (98% acetonitrile/2% water/0.1% formic acid) over 158 min and sprayed into the mass spectrometer via a Nanospray Flex-Ion Source (Thermo Scientific) at a voltage of 1.5 kV. Full MS scans were collected in the orbitrap with a resolution of 120,000, across a range from 350 to 1350 m/z, an automatic gain control (AGC) target of 1 × 10^6^, and a maximum injection time of 50 ms. MS^2^ ions were then selected with an isolation width of 0.5 Da, an AGC of 1.5 × 10^4^, and a maximum injection time of 100 ms using a top speed data dependent mode. Fragmentation occurred with CID energy of 35 and subsequent analysis in the ion trap. MS^3^ spectra were acquired in the orbitrap by isolating 8 MS^2^ ions in synchronized precursor selection (SPS) mode and fragmenting with a higher collision dissociation energy (HCD) of 55, AGC of 1.5 × 10^5^, a maximum injection time of 150 ms, isolation width of 1.2 Da, and a resolution of 50,000 at 200 m/z.

#### Proteomic data analysis

Raw files were converted to mzXML using ReadW (v4.3.1) available through https://sourceforge.net/projects/sashimi/files/ReAdW%20(Xcalibur%20converter)/. MS/MS spectra were searched using Mascot (v 2.4.1) licensed from Matrix Sciences with protein sequences comprising of UniProt DB (2017_08) prefiltered with a taxonomy ‘9606’, common contaminating proteins and all decoy sequences. Search parameters included trypsin cleavage with allowance of up to 2 missed cleavage events, a precursor ion tolerance of 50 ppm, and a fragment ion tolerance of 0.8 Da. Searches included variable modifications of methionine oxidation (+15.9949 Da), static carbamidomethylation modification of cysteine (+57.0215 Da), static TMT modification of lysine and peptide n terminus (+229.1629 Da for 10plex TMT or +304.2071 Da for 16plex TMTpro). Peptide spectra matches (PSMs) were filtered with a false discovery rate (FDR) of 2% at the peptide level and 2% at the protein level using linear discrimination (Huttlin et al., 2010). TMT reporter ions were quantified with Mojave (Vartanian et al., 2016) by calculating the highest peak within 20 ppm of theoretical reporter mass windows and correcting for isotope purities. Mojave is an in-house tool developed to report TMT reporter ion intensity values and is available upon request. Quantified PSMs were filtered by total TMT reporter ion intensity greater than 30,000 and isolation specificity greater than 0.7. Peptides shorter than 7 residues were removed prior to downstream analyses in R (version 3.5.0).

For the classical TPP, quantitative data from each temperature were used to generate a thermal titration curve and calculate a melting point. PSM level quantitation was log2 transformed and summarized to protein level by aggregating all PSMs from the same primary Uniprot reference, and systematic variation between the treatments was normalized at each temperature. A thermal titration curve was fitted from experimental data for each protein and for each treatment condition using Self-Starting Nls Four-Parameter Logistic Model from stats v3.6.2 in R. Melting point was calculated at the 50% between intensity at the lowest temperature and intensity at the highest temperature. A quality score is calculated for each protein by evaluating R squares, curve slopes and similarity in slopes between the two fitted curves. For the PISA experiment, quantitative data from each replicate of combined temperatures were used to calculate fold changes and p values between the treatment conditions. Quantified PSMs were normalized based on global median abundance among all PSMs, and then summarized to the protein level using statistical package MSstats_3.14.1 (Choi et al., 2014). Fold changes and p values were calculated between pre-defined treatment groups by the MSstats package. For the photoaffinity probe based pull-down study, pairwise differential analysis was applied to treatment groups. TMT abundance of all PSMs was aggregated to their respective proteins. Ratios between each treatment group were calculated based on their mean protein abundance from replicates and a Student’s t test was applied to each pair to calculate p values. Fold-change tables for PAL and TPP analyses are provided as supplemental datasets.

#### Generation of recombinant PFK1 and *in vitro* kinase assays

Recombinant N-terminal His-tagged human PFKL (NP_002617), PFKM (NP_000280), and PFKP (NM_002627.4) were generated as described (Webb et al., 2015, 2017). Point mutants of PFK1 were generated using the QuikChange Lightning kit (Aligent).

His-tagged PFK1 was expressed and purified as described with slight modifications (Webb et al., 2015, 2017). Briefly, baculovirus was generated using the Bac-to-Bac Expression system (Invitrogen). PFK1 was expressed in 2-5 × 10^6^ sf9 cells at a multiplicity of infection of two for 48 h. Cells were lysed in 20 mM HEPES, pH 7.5, 80 mM potassium phosphate, 1 mM 2-mercapentoethanol, 10% glycerol, 10 mM imidazole, and EDTA-free Protease Inhibitor Cocktail (Abcam) with a Dounce homogenizer. The soluble lysate was incubated with HisPur cobalt resin (ThermoFisher Scientific). The resin was washed with 5 bead volumes lysis buffer, 10 bead volumes lysis buffer containing 2 M NaCl, and 5 further bead volumes lysis buffer. PFK1 was eluted in lysis buffer containing 100 mM imidazole. Fractions containing protein were pooled, passed over a desalting column equilibrated in freezing buffer [20 mM HEPES pH 7.5, 1 mM DTT, 500 μM ammonium sulfate, 5% glycerol, 1 mM ATP, and 100 μM EDTA] and concentrated using an Amicon Ultracel-30K Centrifugal Filter Unit (MilliporeSigma). Protein was quantified using a Bradford Protein Assay kit (ThermoFisher Scientific), and aliquotes frozen in liquid nitrogen for storage at −80°C.

#### PFK1 activity assays

PFK1 activity was determined using the rate of FBP production via an auxiliary enzyme assay adapted to a 96-well format (Brüser et al., 2012; Webb et al., 2015). The auxiliary enzymes aldolase, triosephosphate isomerase, and glycerol phosphate dehydrogenase were purchased from MilliporeSigma as ammonium sulfate slurries and desalted using an Amicon Ultracel-10K Centrifugal Filter Unit. For assays containing FBP, the rate of ADP production was determined using the auxiliary enzymes pyruvate kinase (PK) and lactate dehydrogenase (LDH) (MilliporeSigma) in a buffer containing 250 μM phosphoenolpyruvic acid, 250 μmM NADH, 50 mM HEPES pH 7.5, 100 mM KCl, 1 mM DTT, and effector concentrations as described in the text. The temperature was equilibrated to 25°C for 5 min before initiating the reaction 10 mM MgCl_2_. Absorbance at 340 nm was measured using a Varioskan LUX Multimode microplate reader (ThermoFisher). Kinetic parameters were generated by nonlinear regression analysis using Prism (GraphPad Software) and are the mean of a minimum of three measurements from two independent preparations of protein. One unit of activity was defined as the amount of enzyme that catalyzed the formation of 1 μmol of fructose 1,6-bisphosphate per min at 25°C. Assays for PFKM were performed at pH 7.0 due to the pH-dependence of ATP inhibition (Trivedi and Danforth, 1966).

PFK1 activity assays from neutrophil lysates were modified from Abrantes et al., 2012. Briefly, neutrophils (10^7^) were cultured with 0.5 μM NA-11 for 30 min, and then stimulated with 50 nM PMA, 5 μg/mL cholesterol crystals, 10 μg/mL LPS or 25 μM nigericin for 30 min. Cells were lysed in 10 mM PBS, 1% Triton X-100, and 1 x EDTA-free protease inhibitor cocktail on ice for 10 min. The soluble protein lysates were quantified by BCA assay (Pierce) and adjusted to 0.3125 mg/mL with lysis buffer. Lysate (80 μL) was mixed with 100 μL 2x enzyme cocktail solution (100 mM Tris-HCL pH7.4, 10 mM MgCl_2_, 0.5 mM ATP, 0.3 mM NADH, 1.35 units/mL Aldolase, 10 units/mL Triose phosphate isomerase, 4 units/mL glycerophosphate dehydrogenase, 20 mM ammonium sulfate) equilibrated to reach room temperature for 5 min. 20 μL of Fructose-6-phosphate (40 mM) was added and the reaction monitored for NADH decrease (absorbance at 340 nm) every 30 s for 30 min. Specific activity is in pmoles FBP per min per mg lysate.

#### Thermal inactivation assay

Thermal inactivation assays were performed as previously described for PFKP, with modifications to account for the decreased stability of PFKL (Sánchez-Martínez et al., 2000). PFKL was diluted to 10 ng/mL, with a final buffer composition of 53.3 mM HEPES, 100 mM KCl, 0.167 mM DTT, 83.3 μM ammonium sulfate, 0.83% glycerol, 0.170 mM ATP, and 16.7 mM EDTA. Samples were incubated at 35°C. At the times indicated, samples were removed and residual PFK1 activity measured using the PK/LDH auxiliary enzyme assay described above with 125 μM ATP and 5 mM F6P. To determine if small molecule ligands altered PFKL stability, 200 μM FBP and 200 nM NA-11 were added.

#### Cryo-electron microscopy

9 μM purified PFKL was combined with 1 mM ATP, 2 mM F6P, and 50 μM NA-11 in 20 mM HEPES pH 7.5, 1 mM DTT, 500 μM ammonium sulfate, 5% glycerol, and 100 μM EDTA. The PFKL sample was applied to glow-discharged CFLAT 2/2 holey-carbon grids (Protochips) and blotted away 4 times successively, before being plunged into liquid ethane using a Vitrobot (ThermoFisher). Data collection was performed on a Titan Krios (ThermoFisher) equipped with a Quantum GIF energy filter (Gatan Inc.) operating in zero-loss mode with a 20 eV slit width. Movies were acquired on a K-2 Summit Direct Detect camera (Gatan Inc.), operating in super-resolution mode with a pixel size of 0.525 Å/pixel, with 50 frames and a total dose of 90 electrons/Å2. Images were collected at both 0° and 30° tilt. Data collection was automated using Leginon software (Suloway et al., 2005). Data collection and refinement statistics are listed in Table S2.

#### Cryo-electron microscopy image processing

Workflows for cryo-EM data processing of NA-11-bound PFKL are shown in Figures S5 and S6. Movie frames were aligned, dose-weighted and summed using MotionCor2 (Zheng et al., 2017). CTF parameters were estimated using CTFFIND4 (Rohou and Grigorieff, 2015) for un-tilted images, and cryoSPARC (Punjani et al., 2017) patch CTF estimation for 30° tilted images. Automated particle picking and 2D classification were performed in cryoSPARC. Particles from selected 2D classes were then exported to Relion (Scheres, 2012) for 3D auto-refinement and 3D classification. Beamtilt, anisotropic magnification, and defocus refinement as well as Bayesian particle polishing were also performed in Relion. Density modification to produce final maps was performed using the ResolveCryoEM application in Phenix (Adams et al., 2010; Terwilliger et al., 2020).

#### ROS detection assay

ROS were measured using the ROS-Glo bioluminescent assay (Promega) for the direct detection of H_2_O_2_. Briefly, neutrophils (2 × 10^4^) were plated in white 96-well plates (Greiner) in high glucose DMEM medium. Cells were cultured with DMSO vehicle, 0.5 μM NA-11, 5 μM LDC7559, 100 μM apocynin, or 200 μM thionicotinamide for 30 min, and then stimulated with 50 nM PMA or treated with 10 μg/mL zymosan beads, pre-opsonized in 20% serum for 30 min. Luminescence, after the addition of H_2_O_2_ substrate solution of 20 min, and then ROS-Glo detection solution for 20 min, was recorded on a plate reader (Envision).

#### NADPH/NADP^+^ detection assay

The ratio of NADPH/NADP^+^ (or NADH/NAD^+^) was calculated after measuring the individual concentrations of NADPH and NADP^+^ (or NADH and NAD^+^) with NADP/NADPH-Glo (or NAD/NADH-Glo) assays (Promega). Neutrophils (2 × 10^4^) were cultured in white 96-well plates (Greiner) in high glucose DMEM medium containing DMSO vehicle, 0.5 μM NA-11, 5 μM LDC7559, 100 μM Apocynin, or 200 μM 6-aminonicotinamide for 30 min, and then treated with 50 nM PMA. Cells were lysed in plates by addition of 1% dodecyltrimethylammonium bromide (DTAB). To measure NADPH, half of the samples was heated at 60^°^C for 30 min. The other half was treated with HCl before heating to 60°C, to measure NADP^+^. All samples were neutralized before adding luciferase detection reagent, then incubated for 1 h with gentle shaking. Luminescence was recorded on a plate reader (Envision).

#### Phagocytosis assay

Neutrophils (1 × 10^6^) were cultured in 6-well tissue in RPMI medium containing DMSO vehicle, 0.5 μM NA-11, 0.5 μM NA-4, 100 μM Apocynin or 100 μM thionicotinamide (TN) for 30 min. *E. coli* grown in LB broth overnight (2 mL) were opsonized with human serum (1 mL pooled from three donors) for 1 h at room temperature. Opsonized bacteria were washed with PBS, resuspended in LB broth to an OD_600_of 0.1 and then added to cultured neutrophils at an MOI of 1 for 30 min. Cells were washed with medium three times and then cultured with 300 μg/mL gentamicin for 2 h to kill any extracellular bacteria. Cells were then washed again, twice with medium and once with PBS. To release phagocytosed bacteria, neutrophils were lysed in 0.05% saponin in PBS. Lysates were passed through a 25’ gauge needed several times and the number of viable phagocytosed bacteria was determined by colony counting.

#### Epithelial barrier tissue damage assay

Human bronchial epithelial cells (HBECs) (4 × 10^4^ in 50 mL complete PneumaCult-Ex plus medium) were added to a HTS Transwell-96 permeable support plate (polyester membrane with a 0.4 μm pores) and immersed in receiver plates containing 150 μL of complete PneumaCult-Ex plus medium for 3 days. The medium on confluent monolayers was then replaced with RPMI medium containing neutrophils (4 × 10^4^) in RPMI buffer that were previously treated with 5 μM NA-11, 100 μM apocynin, 5 μM NA-4, or DMSO vehicle and then activated with 100 nM PMA. The transwell plate was cultured in a new receiver plate containing 150 μL Hank’s balanced salt solution (HBSS) for 2 h. 10 μL of DMEM supplemented with 5% FBS and 2 mM L-glutamine were added to each well and the plate was incubated for additional 18 h. To measure the permeability of the epithelial barrier, FITC-dextran (200 μg/mL) and DNase-1 (22 Units/mL) were added to the apical side for 1 h. FITC-dextran in the receiver plate was measured (ex: 494 em: 521).

### QUANTIFICATION AND STATISTICAL ANALYSIS

All data summarization, visualization, and statistical analyses were performed using GraphPad Prism v9.1.2 (GraphPad Software, San Diego, CA). Unless otherwise noted in the text, n represents the number of biological replicates using cells from different donors or independent cultures. Detailed descriptions of quantifications and statistical analyses (exact values of n and statistical tests used) can be found in the figures, figure legends, or methods section. Differences between groups were considered significant if *p* was less than 0.05. No methods were used for sample randomization or sample size estimation and no data were excluded from analyses.

## Supplementary Material

Data S1

Data S2

Document S1. Tables S1 and S2 and Methods S1

4

## Figures and Tables

**Table 1.** Kinetic properties of PFK1 constructs

	F6P						
				
	V_max,F6P_	h_F6P_	K_M,F6P_ (mM)	n	EC_50, NA-11_ (n)	EC_50, NA-5_ (n)	EC_50, LDC7559_ (n)
PFKL							
Wild type	13.28 ± 1.69	3.16 ± 0.89	1.97 ± 0.24	7	14.00 ± 2.91 (8)	19.19 ± 6.79 (4)	66.04 ± 18.93 (7)
PFKL_3Mut_	17.36 ± 0.87	3.07 ± 0.87	0.57 ± 0.06	6	ND (6)	ND (5)	
PFKL_−pocket_	14.68 ± 0.98	3.26 ± 0.48	2.06 ± 0.12	5	166.99 ± 27.83 (6)	614.64 ± 137.89 (4)	
PFKL_−hook_	11.50 ± 0.57	2.87 ± 0.50	1.20 ± 0.09	4	53.28 ± 5.77 (6)	18.06 ± 3.19 (5)	
PFKM							
Wild type	19.14 ± 0.74	4.62 ± 1.38	0.61 ± 0.05	5	ND	ND (5)	ND (4)
PFKM_+pocket_	17.29 ± 0.93	3.64 ± 0.69	1.41 ± 0.08	5	ND	10.94 ± 1.28 (5)	

F6P titrations performed at pH 7.5 with 225 μM ATP (PFKL) or at pH 7.0 with 250 μM ATP (PFKM). The maximal velocity of reaction (V_max,F6P_) is in micromoles FBP per minute per milligram PFK1. K_M,F6P_ is the Michaelis constant. h_F6P_ is the Hill coefficient. NA-11, LDC7559, and NA-5 titrations were performed at pH 7.5 with 3.1 mM ATP (PFKL) or at pH 7.0 with 4.0 mM ATP (PFKM). F6P concentrations were 4 mM (PFKL wild type, PFKL_−pocket_), 2.5 mM (PFKL_−hook_), 1.5 mM (PFKL_3Mut_), 0.5 mM (PFKM wild type), or 1.0 mM (PFKM_+pocket_). Data are the mean ± SEM. n, number of determinations; ND, not determined.

**Table:** KEY RESOURCES TABLE

REAGENT or RESOURCE	SOURCE	IDENTIFIER
Antibodies		
Anti-Histone H3 (citrulline R2 + R8 + R17) antibody	Abcam	ab5103; RRID:AB_304752
Anti-Myeloperoxidase (MPO)	DAKO	A0398; RRID:AB_2335676
Anti-Phosphofructokinase liver type (PFKL)	Abcam	ab181064
Anti-Gasdermin D (GSDMD) L60	Cell Signaling Tech.	93709S; RRID:AB_2800210
Anti-cleaved Gasdermin D (Asp275) E7H9G	Cell Signaling Tech.	36425S; RRID:AB_2799099
Anti-ERK1/2 (p44/42 MAPK)	Cell Signaling Tech.	9102S; RRID:AB_330744
Anti-phospho ERK (pERK1/2)	Cell Signaling Tech.	9101S; RRID:AB_331646
Anti-phosphoMEK1/2 (pMEK) 4169	Cell Signaling Tech.	9154S; RRID:AB_2138017
Anti-Tubulin	Abcam	ab6046; RRID:AB_2210370
Anti-Neutrophil elastase (NE)	Abcam	ab68672; RRID:AB_1658868
Anti-GAPDH HRP-conjugated 14C10	Cell Signaling Tech.	3683S; RRID:AB_1642205
Bacterial and virus strains		
*E. coli,* Top 10 One Shot, cloning strain	Invitrogen	C404010
Biological samples		
Peripheral blood human neutrophils	Genentech	N/A
THP-1 cell line	Genentech	CL586040
Chemicals, peptides, and recombinant proteins		
Histopaque 1119	Sigma-Aldrich	11191
Histopaque 1077	Sigma-Aldrich	10771
Phorbol 12-myristate 13-acetate (PMA)	Sigma-Aldrich	P1585
Cholesterol	Sigma-Aldrich	C3045
SYTOX Green	Invitrogen	S7020
Lipopolysaccharide (LPS) from *E. coli* strain 0111	Fisher Scientific Co	NC0202558
Lipopolysaccharide (LPS) from *Salmonella enterica*	Sigma-Aldrich	L6143
Nigericin	Tocris	4312
Calcymicin (A23187)	Sigma-Aldrich	100105
Poly(deoxyadenin-deoxytymidine) poly(dA-dT)	Invivogen	Tlrl-patn
Lipofectamine 2000	Invitrogen	11668-019
Recombinant GSDMD, human	Genentech	N/A
Recombinant Caspase-4	Abcam	ab51994
Recombinant Neutrophil Elastase	Abcam	ab91099
Recombinant PFKL, human	This paper	NP_002617
Recombinant PFKM, human	This paper	NP_000280
Recombinant PFKP, human	This paper	NM_002627.4
Zymosan, A form *Saccahromyces cerevisiae*	Fisher Scientific Co	NC9008227
Zymosan, A form *S. cerevisiae* Texas Red Conjugate	Fisher Scientific Co	Z2843
FITC-Dextran	Sigma-Aldrich	FD4-100MG
PneumaCult-Ex Plus Medium kit	StemCell Tech	05040
Hydrocortisone	StemCell Tech	07925
ROCK inhibitor Y-27632	Sigma-Aldrich	SCM075
Critical commercial assays		
CellTox cytotoxicity assay	Promega	G8741
MSD Cytokine Assay, 96-well multi-array Tissue Culture Kit	Meso Scale Discovery	K151AGB-2
ROS-Glo H_2_O_2_ assay	Promega	G8821
NAD/NADH-Glo assay	Promega	G9082
NADP/NADPH-Glo assay	Promega	G9072
EasySep human monocyte isolation kit	StemCell Tech.	19359
Neutrophil Elastase Activity Assay Kit	Abcam	ab204730
NAMPT Inhibitor Screening Assay Kit	BPS Bioscience	71276-1
Deposited data		
Human phosphofructokinase-1 liver type bound to activator NA-11	This study	PDB: 7LW1 EMDB: EMD-23544
Mass spectrometry raw data and PSM deposited on MassIVE database (https://massive.ucsd.edu/ProteoSAFe/static/massive.jsp)	This study	MSV000087015
Experimental models: cell lines		
HBEC cell line UNCN1T	Genentech	RRID:CVCL_ZC91
Recombinant DNA (list of primers)		
PFKL Val545Leu Sense: gctacctggccaccatgactggcattgct Antisense: agcaatgccagtcatggtggccaggtagc	This paper	N/A
PFKL Val582Met Sense: ggctccgacactgctttaaatgccgccatgg Antisense: ccatggcggcatttaaagcagtgtcggagcc	This paper	N/A
PFKL Lys315Arg Sense: gatcctgagcagcaggatgggcatggagg Antisense: cctccatgcccatcctgctgctcaggatc	This paper	N/A
PFKM Arg315Lys Sense: gcttccacacccatcttgctgcccagaattc Antisense: gaattctgggcagcaagatgggtgtggaagc	This paper	N/A
Software and algorithms		
MATLAB	Mathworks	R2017b
GraphPad Prism	GraphPad Software	v9.1.2
cryoSPARC	Punjani et al., 2017	https://cryosparc.com
Relion 3	Scheres, 2012	https://github.com/3dem/relion
ReadW (v4.3.1)	SourceForge	https://sourceforge.net/projects/sashimi/files/ReAdW%20(Xcalibur%20converter)/

## Highlights

- Small molecules LDC7559 and NA-11 inhibit the phagocytic oxidative burst
- NA-11 dampens flux through the pentose phosphate pathway to limit cellular NADPH
- Cryo-EM confirms agonism of PFKL through binding at the AMP/ADP allosteric pocket
- PFKL is a regulatory node for NOX2-dependent NETosis and tissue damage
