# Supplementary material for: Selective activation of PFKL suppresses the phagocytic oxidative burst
Source: Cell. Author manuscript; Available in PMC 2022 Jan 31. (PMC8802628; doi:10.1016/j.cell.2021.07.004)
Supplement: Document S1. Tables S1 and S2 and Methods S1 [file NIHMS1770610-supplement-Document_S1__Tables_S1_and_S2_and_Methods_S1.pdf]

Cell, Volume *184*

Supplemental information

Selective activation of PFKL suppresses the phagocytic oxidative burst

Neri Amara, Madison P. Cooper, Maria A. Voronkova, Bradley A. Webb, Eric M. Lynch, Justin M. Kollman, Taylur Ma, Kebing Yu, Zijuan Lai, Dewakar Sangaraju, Nobuhiko Kayagaki, Kim Newton, Matthew Bogyo, Steven T. Staben, and Vishva M. Dixit

Supplemental Information

Selective Activation of Phosphofructokinase Liver Type Suppresses the Phagocytic Oxidative Burst.

Neri Amara, Madison P. Cooper, Maria A. Voronkova, Bradley A. Webb, Eric M. Lynch, Justin M. Kollman, Taylur Ma, Kebing Yu, Zijuan Lai, Dewakar Sangaraju, Nobuhiko Kayagaki, Kim Newton, Matthew Bogyo, Steven T. Staben, and Vishva M. Dixit.

Correspondence to V.M.D. (dixit@gene.com)

Table S1. Related to Figure 3. Summary of effects of various effectors on NA-11 activity to PFKL

	**$EC_{50, NA-11}$ (n)**	
**pH**		
pH 8.0	4.11 ± 0.92 (4)	
pH 7.5	21.81 ± 6.21 (4)	
pH 7.0	73.98 ± 20.86 (4)	
**Ligands**		
	**$EC_{50, NA-11}$ (n)**	**V_{max} (n)**
Control	11.98 ± 2.73 (4)	18.19 ± 1.29 (4)
50 μM AMP	12.34 ± 3.38 (3)	18.11 ± 1.23 (3)
200 μM FBP	12.04 ± 6.99 (4)	21.31 ± 2.44 (4)

Assays were performed at pH 7.5, unless otherwise indicated, with 4.0 mM F6P and 3.1 mM ATP. NA-11 affinity is reported as the half maximal effective concentration ($EC_{50,NA-11}$). The maximal velocity of reaction ($V_{max,F6P}$) is in μmoles FBP per min per mg PFK1. Means ± s.e.m. are reported with the number of determinations (n) for each condition.

Table S2. Related to STAR methods. EM data collection and refinement statistics.

	NA-11-bound PFKL tetramer (EMD-23544, PDB 7LW1)
**Data collection**	
Electron microscope	Titan Krios
Voltage (kV)	300
Electron detector	K2 summit
Exposure time (s)	10
Total electron exposure (e^-/Å^2)	90
Magnification (nominal)	130,000X
Super-resolution pixel size (Å)	0.525
Frames/movie	50
Micrographs collected	2529
Defocus range (μm)	-0.7 to -2.8
**Reconstruction**	
Pixel size (Å)	1.05
3D refinement package	Relion
Point group symmetry	D2
Particles	63296
Resolution (0.143 FSC) (Å)	3.1
Resolution, density modified (0.5 FSCref) (Å)	2.9
**Model composition**	
Protein residues	744
Ligands	ADP, F6P, FBP, NA-11
**Validation**	
Clashscore	2
Poor rotamers (%)	1
Molprobity score	1.5
RMSZ bond lengths	0.68
RMSZ bond angles	1.12
**Ramachandran plot**	
Favored (%)	95
Allowed (%)	4
Outliers (%)	1

Methods S1. Relates to STAR methods. Synthesis procedures.

Abbreviations

AcOH – Acetic acid

ACN – Acetonitrile

BuLi – Butyllithium

DCM – Dichloromethane

DIAD – Diisopropyl azidocarboxylate

DME – Dimethoxyethane

EDCI – 1-Ethyl-3-(3-dimethylaminopropyl)carbodiimide

EtOAc – Ethyl acetate

EtOH – Ethanol

HATU – Hexafluorophosphate azabenzotriazole tetramethyl uronium

DIPEA – *N,N*-diisopropylethylamine

HMPA – Hexamethylphosphoric triamide

MeOH – Methanol

MTBE – Methyl tert-butyl ether

NIS – *N*-Iodosuccinimide

RT – Room temperature

TBSCl – Tert-Butyldimethylsilyl chloride

TBAF – Tetra-n-butylammonium fluoride

TEA – Triethylamine

THF – Tetrahydrofurane

TLC – Thin layer chromatography

TosCl – 4-toluenesulfonyl chloride

SYNTHESIS PROCEDURES

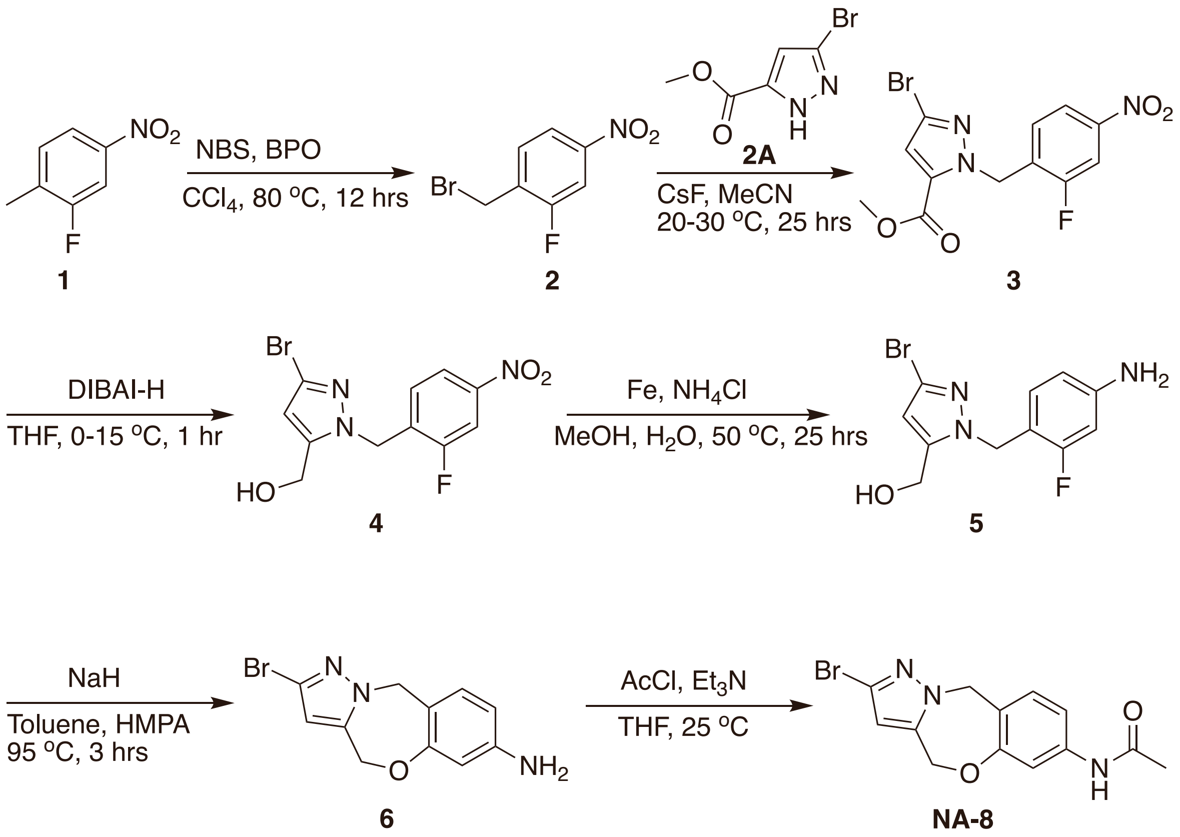

1-(bromomethyl)-2-fluoro-4-nitrobenzene (2): To a solution of **1** (120 g, 774 mmol) in CCl_4 (840 mL) at 80°C was added BPO (12.0 g, 49.5 mmol) and NBS (151 g, 851 mmol). The mixture was stirred at 80°C for 12 h. TLC (petroleum ether/ethyl acetate = 20/1, reactant 1 R_f = 0.40, product R_f = 0.30) showed the reaction was complete. The reaction mixture was cooled to RT. The residue was poured into ice-water (600 mL) and stirred for 10 min. The aqueous phase was extracted with DCM (300 mL x 3). The combined organic phase was washed with brine (300 mL x 1), dried with anhydrous Na_2SO_4, filtered and concentrated under vacuum. The residue was purified by column chromatography (SiO_2, petroleum ether/ethyl acetate = 400/1 to 30/1) then

filtered and concentrated under vacuum. Compound **2** (120 g, 513 mmol, 66.3% yield) was obtained as a yelllow solid.

Methyl 3-bromo-1-(2-fluoro-4-nitrobenzyl)-1*H*-pyrazole-5-carboxylate (3): To a solution of compound **2** (30.0 g, 146 mmol) in ACN (210 mL) was added CsF (44.5 g, 293 mmol) and compound **2A** (37.7 g, 161 mmol). The mixture was stirred at 25°C for 5 h. TLC (petroleum ether/ethyl acetate = 3/1, reactant R_{f1} = 0.30, reactant R_{f2} = 0.60, product R_f = 0.45) showed the reaction was incomplete. The mixture concentrated under vacuum to give a crude residue. The residue was purified by column chromatography (SiO_2, petroleum ether/ethyl acetate = 80/1 to 3/1). Compound **3** (31.0 g, 86.6 mmol, 59.1% yield) was obtained as a white solid.

(3-bromo-1-(2-fluoro-4-nitrobenzyl)-1*H*-pyrazol-5-yl)methanol (4): To a mixture of compound **3** (62.0 g, 173 mmol) in THF (620 mL) was added DIBAL-H (1.00 M, 346 mL) at 0°C. The mixture was stirred at 15°C for 1 h under N_2 atmosphere. TLC (Plate 1: petroleum ether/ethyl acetate = 2/1, reactant 1 R_f = 0.50, product R_f = 0) indicated compound **3** was consumed completely and one main spot formed. The reaction mixture was quenched by addition of saturated NH_4Cl solution (300 mL). The resulting mixture was dissolved in EtOAc/EtOH = 1000 mL/200 mL and filtered to remove the insoluble. The filtrate was concentrated under vacuum. The residue was purified by column chromatography (SiO_2, petroleum ether/ethyl acetate = 5/1 to 0/1, plate 2: petroleum ether/ethyl acetate = 1/1, compound **4** R_f = 0.35). The combined organic layers were concentrated under vacuum. Compound **4** (50.9 g, 154.2 mmol, 89.0% yield) was obtained as a white solid.

(1-(4-amino-2-fluorobenzyl)-3-bromo-1*H*-pyrazol-5-yl)methanol (5): To a solution of compound **4** (50.9 g, 154.2 mmol) in MeOH (510 mL) and H_2O (51.0 mL) was added Fe (34.4 g, 617 mmol) and NH_4Cl (41.2 g, 771 mmol). The mixture was stirred at 50°C for 3 h, then additional Fe (8.61 g, 154 mmol) and NH_4Cl (16.5 g, 308 mmol) were added. The mixture was stirred at 50°C for 22 h. TLC (petroleum ether/ethyl acetate = 1/1, reactant 1 R_f = 0.57, product R_f = 0.47) indicated compound **4** was consumed completely and one main spot formed. The reaction mixture was quenched by addition saturated NH_4Cl solution (200 mL), then washed with EtOAc (200 mL x 3). The combined organic layers were concentrated under vacuum and the residue was extracted

with DCM (200 mL x 3). The combined organic layers were washed with brine (200 mL), dried over sodium sulfate, filtered and concentrated under vacuum. Compound **5** (40.3 g, 134 mmol, 87.0% yield) was obtained as a yellow solid.

2-bromo-4*H*,10*H*-benzo[*f*]pyrazolo[5,1-*c*][1,4]oxazepin-7-amine (6): Compound **5** (23.0 g, 76.6 mmol) was dissolved in HMPA (230 mL) and added to a suspension of NaH (6.13 g, 153 mmol, 60.0% purity) in toluene (2300 mL) under N_2. The mixture was stirred under N_2 at 95°C for 3 h. TLC (petroleum ether/ethyl acetate = 1/1, reactant 1 $R_f = 0.40$, product $R_f = 0.56$) indicated compound **5** was consumed completely and one main spot formed. The reaction mixture was quenched by addition H_2O (1000 mL) and extracted with toluene (500 mL x 3). The combined organic layers were washed with brine (500 mL), dried over sodium sulfate, filtered and concentrated under vacuum. The residue was purified by column chromatography (SiO_2, petroleum ether/ethyl acetate = 10/1 to 0/1). The combined organic layers were concentrated under vacuum. Compound **6** (7.50 g, 26.7 mmol, 34.9% yield) was obtained as a yellow solid.

***N*-(2-bromo-4*H*,10*H*-benzo[*f*]pyrazolo[5,1-*c*][1,4]oxazepin-7-yl)acetamide (NA-8):** Compound **6** (4.00 g, 14.3 mmol) was dissolved in THF (120 mL). Et_3N (2.89 g, 28.6 mmol, 3.98 mL), then acetyl chloride (1.35 g, 17.14 mmol, 1.22 mL) were added and the mixture was stirred at 25°C for 25 min. TLC (petroleum ether/ethyl acetate = 1/1, compound **6** $R_f = 0.50$, product 1 R_f =0.30) indicated compound **6** was consumed completely and one main spot formed. The reaction mixture was quenched by addition H_2O (100 mL) and extracted with EtOAc (100 mL x 3). The combined organic layers were washed with brine (100 mL), dried over sodium sulfate, filtered and concentrated under vacuum. The residue was purified by column chromatography (SiO_2, petroleum ether/ethyl acetate = 10/1 to 0/1). The combined organic layers were concentrated under vacuum. Compound **NA-8** (3.50 g, 10.8 mmol, 75.6% yield, 99.4% purity) was obtained as a yellow solid.

^{1}H NMR: DMSO 400MHz, δ: 9.96 (s, 1H), 7.30 (d, *J* = 2 Hz, 1H), 7.25 (d, *J* = 8.4 Hz, 1H), 7.14 (d, *J* = 8.4Hz, 1H), 6.45 (s, 1H), 5.49 (s, 2H), 5.28 (s, 2H), 2.52 - 2.55 (m, 1H), 2.02 (s, 3H). **LCMS:** ($M+H^+$): 321.9, calculated 321.01.

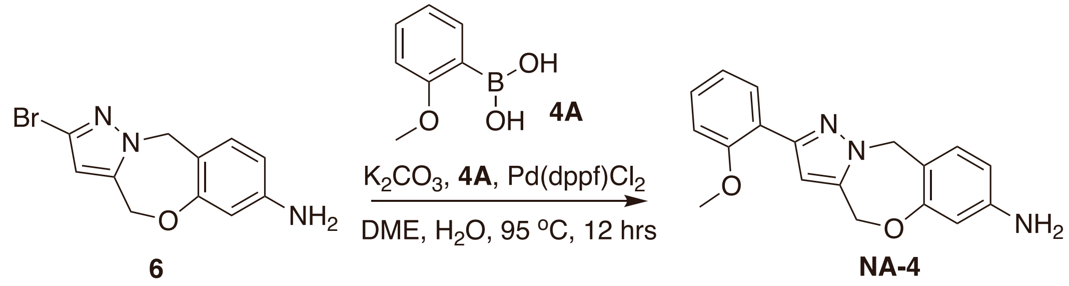

2-(2-methoxyphenyl)-4*H*,10*H*-benzo[*f*]pyrazolo[5,1-*c*][1,4]oxazepin-7-amine (NA-4): A mixture of compound **6** (4.00 g, 14.3 mmol), compound **4A** (2.60 g, 17.1 mmol), Pd(dppf)Cl_2 (2.09 g, 2.86 mmol) and K_2CO_3 (3.95 g, 28.6 mmol) in DME (40.0 mL) and H_2O (4.00 mL) was degassed and purged with N_2 for 3 times. The mixture was stirred at 95°C for 12 h under N_2 atmosphere. TLC (petroleum ether/ethyl acetate = 1/1, compound **6** R_f = 0.43, product R_f = 0.24) showed the reaction was complete. The mixture was poured into H_2O (100 mL) and extracted with EtOAc (50.0 mL x 3). The combined organic layers were washed with brine (20.0 mL), dried over Na_2SO_4, filtered and concentrated under vacuum. The residue was purified by column chromatography (SiO_2, petroleum ether/ethyl acetate = 10/1 to 0/1). Compound **NA-4** (3.00 g, 9.63 mmol, 67.4% yield, 98.6% purity) was obtained as a yellow solid.

^{1}H NMR: DMSO 400MHz, δ: 7.84 (m, *J* = 7.62 Hz, 1H), 7.28 (m, *J* = 8.4 Hz, 1H), 7.07 (m, *J* = 7.82 Hz, 1H), 6.94 - 7.00 (m, 2H), 6.72 (s, 1H), 6.21 (m, *J* = 8.12 Hz, 1H), 6.16 (m, *J* = 2.4 Hz, 1H), 5.40 (s, 2H), 5.24 (s, 2H), 5.15 (s, 2H), 3.84 (s, 3H), 3.32 (s, 2H). **LCMS:** ($M+H^+$): 308.05, calculated 307.13.

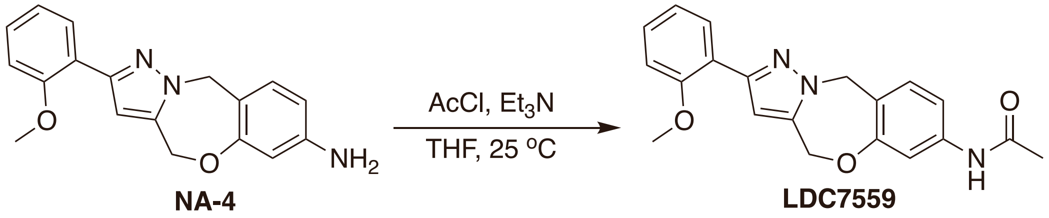

***N*-(2-(2-methoxyphenyl)-4*H*,10*H*-benzo[*f*]pyrazolo[5,1-*c*][1,4]oxazepin-7-yl)acetamide (LDC7559):** Compound **NA-4** (200 mg, 651 µmol) was dissolved in THF (6.00 mL). Et_3N (132

mg, 1.30 mmol), then Acetyl chloride (61.3 mg, 781 µmol) were added and the mixture was stirred at 18°C for 25 min. TLC (petroleum ether/ethyl acetate = 1/1, **NA-4** R_f = 0.50, product R_f = 0.30) indicated **NA-4** was consumed completely and one main spot formed. The reaction mixture was quenched by addition H_2O (15.0 mL) and extracted with EtOAc (15.0 mL x 3). The combined organic layers were washed with brine (10.0 mL), dried over sodium sulfate, filtered and concentrated under vacuum. The residue was purified by column chromatography (SiO_2, petroleum ether/ethyl acetate = 50/1 to 0/1). The combined organic layers were concentrated under vacuum. The residue was purified by HPLC using a Waters Xbridge 150 mm X 25 mm and 5 µm particle size. The mobile phase was composed of 10 mM NH_4HCO_3 (aq) and ACN, gradient of ACN from 25%-55% over 20 min. The collected fractions were concentrated by freeze-drying to yield compound **LDC7559** (188 mg, 532 µmol, 81.7% yield, 98.8% purity) as a white powder.
^{1}H NMR: $CDCl_3$ 400MHz, δ: 7.87 (d, *J* = 7.45 Hz, 1H), 7.27 - 7.37 (m, 3H), 7.14 - 7.20 (m, 1H), 6.94 - 7.11 (m, 3H), 6.69 (s, 1H), 5.51 (s, 2H), 5.27 (s, 2H), 3.89 (s, 3H), 2.16 (s, 3H), 1.91 - 2.05 (m, 2H). **LCMS:** ($M+H^+$): 350.05, calculated 349.14.

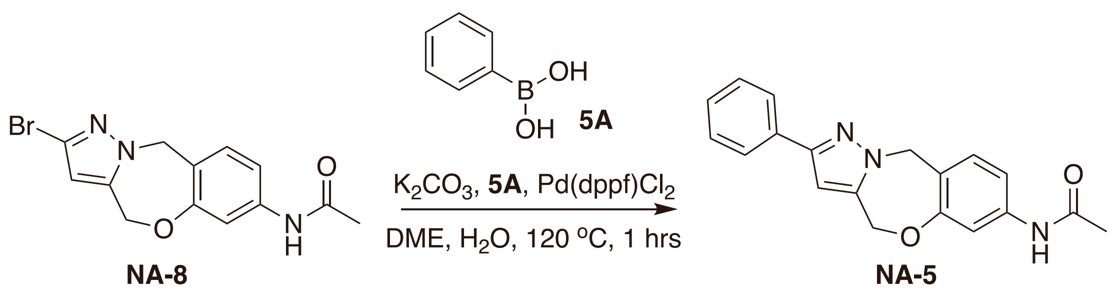

***N*-(2-phenyl-4*H*,10*H*-benzo[*f*]pyrazolo[5,1-*c*][1,4]oxazepin-7-yl)acetamide (NA-5):** Compound **NA-8** (200 mg, 621 µmol), compound **5A** (98.4 mg, 807 µmol), K_2CO_3 (172 mg, 1.24 mmol) and $Pd(PPh_3)_4$ (143 mg, 124 µmol) were suspended in DME (4.00 mL) and H_2O (2.00 mL). The mixture was heated to 120°C for 1 h in a microwave. TLC (petroleum ether/ethyl acetate = 1/1, **NA-8** R_f = 0.35, product R_f = 0.20) indicated compound **NA-8** was consumed completely and one main spot formed. The reaction mixture was quenched by addition H_2O (10.0 mL) and extracted with EtOAc (10.0 mL x 3). The combined organic layers were washed with brine (10.0 mL), dried over sodium sulfate, filtered and concentrated under vacuum. The residue was purified by column chromatography (SiO_2, petroleum ether/ethyl acetate = 10/1 to 0/1, petroleum

ether/ethylacetate = 1/1, product 1 R_f = 0.2). The combined organic layers were concentrated under vacuum. Compound **NA-5** (75.0 mg, 234 μmol, 37.6% yield, 99.5% purity) was obtained as a yellow solid.

^{1}H NMR: DMSO 400MHz, δ: 9.95 (s, 1H), 7.77 (d, *J* = 7.2 Hz, 2H), 7.39 (m, *J* = 7.6 Hz, 2H), 7.26 - 7.32 (m, 3H) 7.14 (d, *J* = 8.4 Hz, 1H), 6.78 (s, 1H), 5.58 (s, 2H), 5.34 (s, 2H), 2.02 (s, 3H). **LCMS:** ($M+H^+$): 320.05, calculated 319.13.

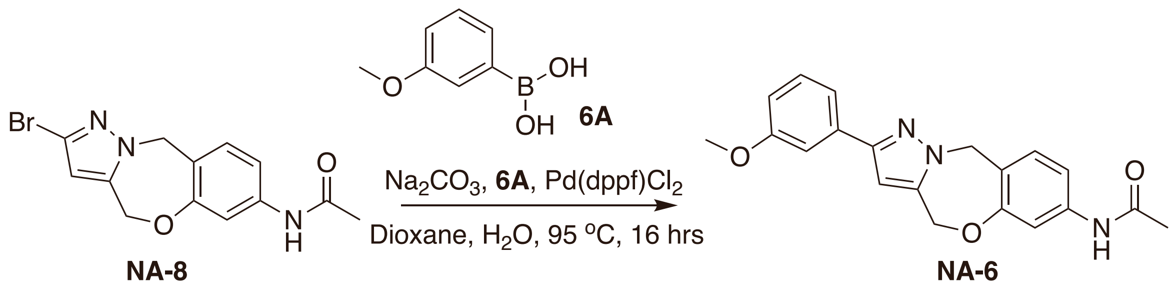

***N*-(2-(3-methoxyphenyl)-4*H*,10*H*-benzo[*f*]pyrazolo[5,1-*c*][1,4]oxazepin-7-yl)acetamide (NA-6): NA-8** (200 mg, 621 μmol), compound **6A** (189 mg, 1.24 mmol), Na_2CO_3 (132 mg, 1.24 mmol) and Pd(dppf)Cl_2 (45.4 mg, 62.1 μmol) were suspended in H_2O (2.00 mL) and dioxane (10.0 mL). The mixture was heated to 95°C for 16 h under N_2 atmosphere. TLC (petroleum ether/ethyl acetate = 1/1, **NA-8** R_f = 0.30, product R_f = 0.18) indicated **NA-8** was consumed completely and one main spot formed. The reaction mixture was quenched by addition H_2O (10.0 mL) and extracted with EtOAc (10.0 mL x 3). The combined organic layers were washed with brine (10.0 mL), dried over sodium sulfate, filtered and concentrated under vacuum. The residue was purified by column chromatography (SiO_2, petroleum ether/ethyl acetate = 10/1 to 0/1). The combined organic layers were concentrated under vacuum. Compound **NA-6** (61.0 mg, 169 μmol, 27.2% yield, 96.7% purity) was obtained as a red brown solid.

^{1}H NMR: DMSO 400MHz, δ: 9.96 (s, 1H), 7.24 - 7.35 (m, 5H), 7.13 (d, *J* = 7.8 Hz, 1H), 6.86 (d, *J* = 7.4 Hz, 1H), 6.79 (s, 1H), 5.57 (s, 2 H), 5.34 (s, 2H), 3.78 (s, 3H), 2.01 (s, 3H). **LCMS:** ($M+H^+$): 350.05, calculated 349.14.

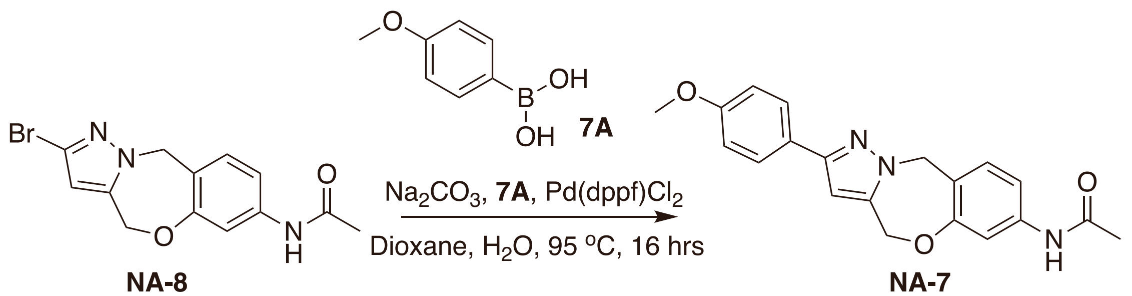

***N*-(2-(4-methoxyphenyl)-4*H*,10*H*-benzo[*f*]pyrazolo[5,1-*c*][1,4]oxazepin-7-yl)acetamide (NA-7):** **NA-8** (200 mg, 621 µmol), compound **7A** (189 mg, 1.24 mmol), Pd(dppf)Cl_2 (45.4 mg, 62.1 µmol) and Na_2CO_3 (132 mg, 1.24 mmol) were suspended in H_2O (2.00 mL) and dioxane (10.0 mL). The mixture was heated to 95°C for 16 h under N_2 atmosphere. TLC (petroleum ether/ethyl acetate = 1/1, **NA-8** R_f = 0.30, product R_f = 0.20) indicated **NA-8** was consumed completely and one main spot formed. The reaction mixture was quenched by addition H_2O (10.0 mL) and extracted with EtOAc (10.0 mL x 3). The combined organic layers were washed with brine (10.0 mL), dried over sodium sulfate, filtered and concentrated under vacuum. The residue was purified by column chromatography (SiO_2, petroleum ether/ethyl acetate = 10/1 to 0/1). The combined organic layers were concentrated under vacuum. Compound **NA-7** (105 mg, 295 µmol, 47.5% yield, 98.2% purity) was obtained as a white solid.

^{1}H NMR: DMSO 400MHz, δ: 9.96 (s, 1H), 7.69 (d, J = 7.8 Hz, 2H), 7.25 - 7.29 (m, 2H), 7.13 (d, J = 8 Hz, 1H), 6.96 (s, 1H), 6.94 (s, 1H), 6.69 (s, 1H), 5.55 (s, 2H), 5.32 (s, 2H), 3.77 (s, 3H), 3.17 - 3.31 (m, 3H), 2.01 (s, 3H). **LCMS:** (M+H$^+$): 350.05, calculated 349.14.

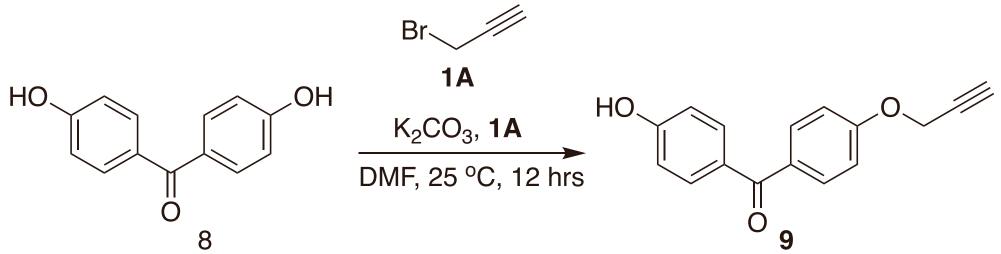

(4-hydroxyphenyl)(4-(prop-2-yn-1-yloxy)phenyl)methanone (9): A mixture of compound **1** (10.0 g, 46.7 mmol, 1.00 eq), K_2CO_3 (9.68 g, 70.0 mmol, 1.50 eq) in DMF (50.0 mL) was degassed and purged with N_2 for 3 times. The mixture was stirred at 25°C for 20 min under N_2 atmosphere. Then compound **1A** (8.33 g, 70.0 mmol, 6.04 mL, 1.50 eq) was added and the mixture was

degassed and purged with N_2 for 3 times. The mixture was stirred at 25°C for 12 h under N_2 atmosphere. TLC (petroleum ether/ethyl acetate = 2/1, compound **8** R_f = 0.45, product R_f = 0.57) indicated compound **8** was consumed completely and one main spot formed. The reaction mixture was quenched by addition H_2O (250 mL) and extracted with EtOAc (100 mL x 3). The combined organic layers were washed with brine (100 mL), dried over sodium sulfate, filtered and concentrated under vacuum. The residue was purified by column chromatography (SiO_2, petroleum ether/ethyl acetate = 20/1 to 2/1). The combined organic layers were concentrated under vacuum. Compound **9** (3.66 g, 14.5 mmol, 31.0% yield) was obtained as a white solid.

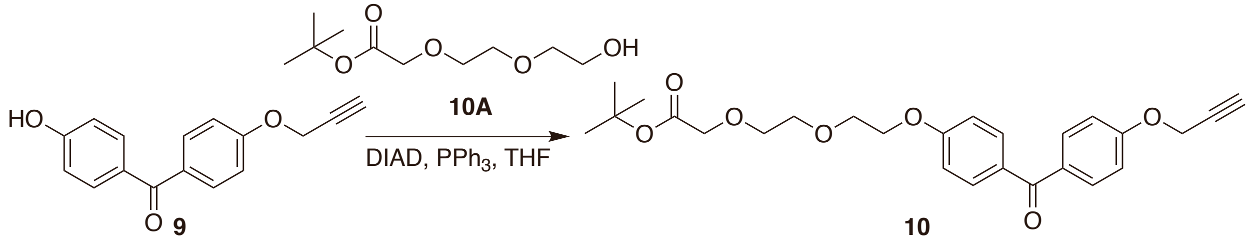

***tert*-butyl 2-(2-(2-(4-(4-(prop-2-yn-1-yloxy)benzoyl)phenoxy)ethoxy)ethoxy)acetate (10):** A mixture of compound **9** (1.50 g, 5.95 mmol, 1.00 eq), compound **10A** (1.57 g, 7.14 mmol, 1.20 eq), PPh_3 (1.87 g, 7.14 mmol, 1.20 eq) and DIAD (1.44 g, 7.14 mmol, 1.39 mL, 1.20 eq) were dissolved in THF (20.0 mL), degassed and purged with N_2. The mixture was then stirred at 25°C for 18 h under N_2 atmosphere. The reaction was monitored by LCMS until completion, after which the mixture was poured into H_2O (30.0 mL) and extracted with EtOAc (20.0 mL x 3). The combined organic layers were washed with brine (10.0 mL), dried over Na_2SO_4, filtered and concentrated under vacuum. The residue was purified by column chromatography (SiO_2, petroleum ether/ethyl acetate = 50/1 to 1/1). Compound **10** (2.50 g, 5.50 mmol, 92.5% yield) was obtained as a colorless oil.

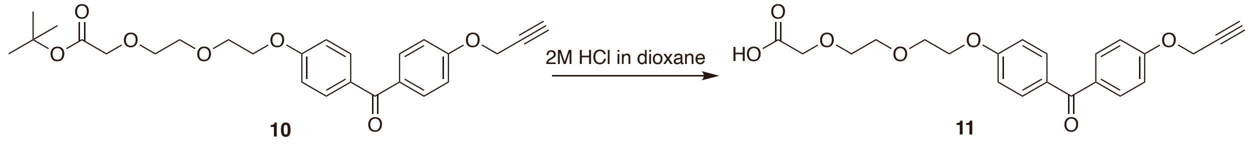

2-(2-(2-(4-(4-(prop-2-yn-1-yloxy)benzoyl)phenoxy)ethoxy)ethoxy)acetic acid (11): To a solution of compound **10** (300 mg, 660 µmol, 1.00 eq) in dioxane (5.00 mL) was added 4M HCl/dioxane (5.00 mL). The mixture was stirred at 25°C for 12 h. TLC (petroleum ether/ethyl acetate = 1/1, compound **10** $R_f = 0.20$, product $R_f = 0.00$) showed the reaction was complete. The reaction mixture was concentrated under vacuum. Compound **4** (260 mg, 475 µmol, 71.9% yield, 72.7% purity) was obtained as a colorless oil.

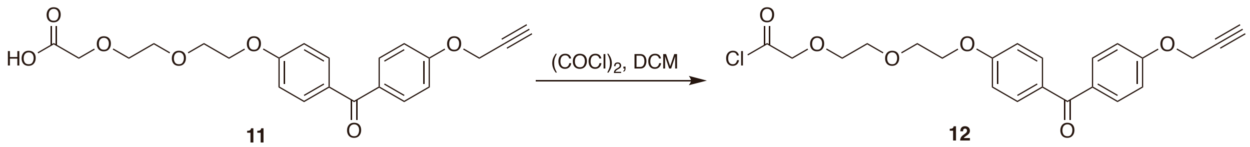

2-(2-(2-(4-(4-(prop-2-yn-1-yloxy)benzoyl)phenoxy)ethoxy)ethoxy)acetyl chloride (12): To a solution of compound **11** (230 mg, 577 µmol, 1.00 eq) in DCM (5.00 mL) was added $(COCl)_2$ (293 mg, 2.31 mmol, 202 µL, 4.00 eq). The mixture was stirred at 25°C for 20 min. TLC (dichloromethane/methanol = 10/1, compound **11** $R_f = 0.24$, product $R_f = 0.84$) showed the reaction was complete. The reaction mixture was concentrated under vacuum. Compound **5** (240 mg, 576 µmol, 99.7% yield) was obtained as a yellow solid.

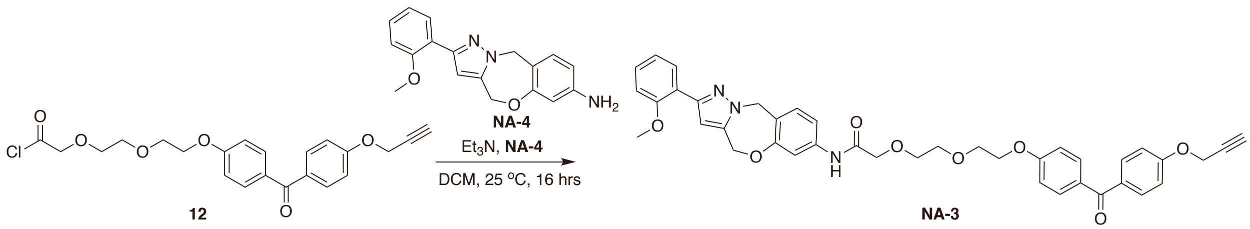

***N*-(2-(2-methoxyphenyl)-4*H*,10*H*-benzo[*f*]pyrazolo[5,1-*c*][1,4]oxazepin-7-yl)-2-(2-(2-(4-(4-(prop-2-yn-1-yloxy)benzoyl)phenoxy)ethoxy)ethoxy)acetamide (NA-3):** To a solution of compound **12** (240 mg, 576 µmol, 1.00 eq) in DCM (10.0 mL) was added Et_3N (175 mg, 1.73 mmol, 240 µL, 3.00 eq) and compound **NA-4** (177 mg, 576 µmol, 1.00 eq). The mixture was stirred at 25°C for 18 h. The reaction was monitored by LCMS until completion, after which the mixture was poured into H_2O (10.0 mL) and extracted with DCM (10.0 mL x 3). The combined organic layers were washed with brine (5.00 mL), dried over Na_2SO_4, filtered and concentrated under vacuum. The residue was purified by column chromatography (SiO_2, petroleum ether/ethyl

acetate = 10/1 to 0/1). Compound **NA-3** (96.3 mg, 131 μmol, 22.8% yield, 93.9% purity) was obtained as a yellow solid.

^{1}H NMR: DMSO 400MHz, δ: 9.63 (s, 1H), 7.81 (dd, *J* = 7.6, 1.6 Hz, 1H), 7.64 - 7.72 (m, 4H), 7.32 (d, *J* = 2.0 Hz, 1H), 7.17 - 7.29 (m, 3H), 7.02 - 7.13 (m, 5H), 6.83 - 7.01 (m, 2H), 6.76 (s, 1H), 5.55 (s, 2H), 5.32 (s, 2H), 4.88 - 4.91 (m, 2H), 4.14 - 4.22 (m, 2H), 4.06 (s, 2H), 3.79 - 3.84 (m, 5H), 3.60 - 3.72 (m, 5H). **LCMS:** ($M+H^{+}$): 688.25, calculated 687.26.

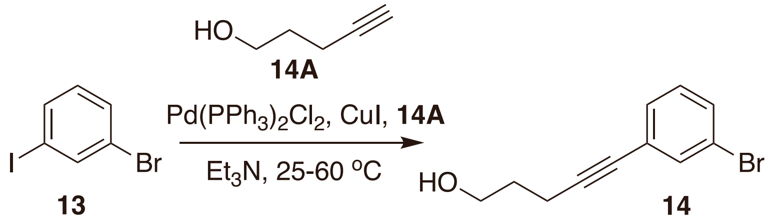

5-(3-bromophenyl)pent-4-yn-1-ol (14): A mixture of compound **13** (2.50 g, 8.84 mmol, 1.13 mL) ,CuI (101 mg, 530 μmol), $Pd(PPh_3)_2Cl_2$ (186 mg, 265 μmol) in Et_3N (20.0 mL) was stirred under N_2 at 25°C for 30 min. Compound **14A** (892 mg, 10.6 mmol) was added and the mixture was stirred at 60°C for 2 h under Ar atmosphere. TLC (petroleum ether/ethyl acetate = 5/1, compound **13** R_f = 0.70, product R_f = 0.20) indicated compound **13** was consumed completely and one main spot formed. The reaction mixture was quenched by addition H_2O (20.0 mL) and extracted with EtOAc (20.0 mL x 3). The combined organic layers were washed with brine (20.0 mL), dried over sodium sulfate, filtered and concentrated under vacuum. The residue was purified by column chromatography (SiO_2, petroleum ether/ethyl acetate = 50/1 to 0/1). The combined organic layers were concentrated under vacuum. Compound **14** (2.00 g, 8.36 mmol, 94.6% yield) was obtained as a brown solid.

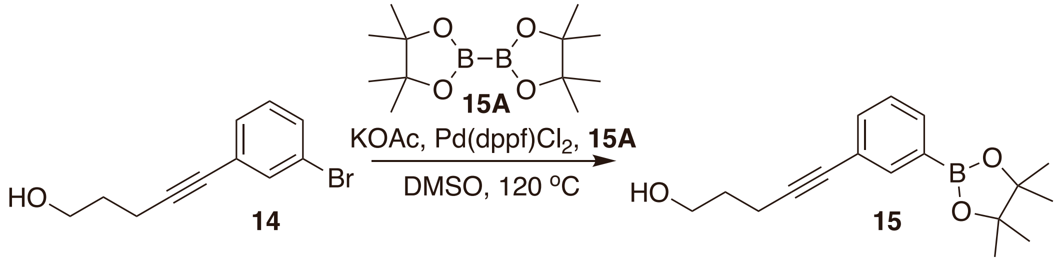

5-(3-(4,4,5,5-tetramethyl-1,3,2-dioxaborolan-2-yl)phenyl)pent-4-yn-1-ol (15): A mixture of compound **14** (1.00 g, 4.18 mmol), KOAc (821 mg, 8.36 mmol) and compound **15A** (1.27 g, 5.02 mmol) in DMSO (10.0 mL) was degassed and purged with N_2 for 3 times. Then Pd(dppf)Cl_2 (306 mg, 418 µmol) was added and the mixture was stirred at 120°C for 18 h under N_2 atmosphere. TLC (petroleum ether/ethyl acetate = 2/1, compound **14** R_f = 0.35, product R_f = 0.3) and HPLC analysis indicated that the reactant **14** was consumed completely and one main spot formed. The reaction mixture was quenched by addition H_2O (50.0 mL) and extracted with EtOAc (50.0 mL x 3). The combined organic layers were washed with brine (30 mL), dried over sodium sulfate, filtered and concentrated under vacuum. The residue was purified by column chromatography (SiO_2, petroleum ether/ethyl acetate = 8/1 to 0/1). The combined organic layers were concentrated under vacuum. Compound **15** (800 mg, 2.80 mmol, 66.8% yield) was obtained as a yellow oil.

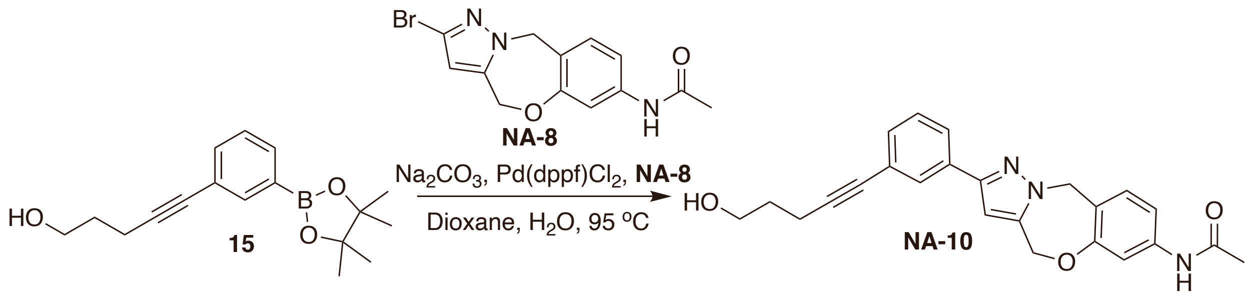

***N*-(2-(3-(5-hydroxypent-1-yn-1-yl)phenyl)-4*H*,10*H*-benzo[*f*]pyrazolo[5,1-*c*][1,4]oxazepin-7-yl)acetamide (NA-10):** Compound **15** (200 mg, 699 µmol), **NA-8** (113 mg, 349 µmol), Pd(dppf)Cl_2 (25.6 mg, 34.9 µmol), and Na_2CO_3 (74.0 mg, 699 µmol) were suspended in H_2O (1.00 mL) and dioxane (5.00 mL) and heated to 95°C for 16 h under N_2 atmosphere. TLC (petroleum ether/ethyl acetate = 1/1, **NA-8** R_f = 0.30, product R_f = 0.20) and LCMS analysis indicated that the reactant **15** was consumed completely and one main spot formed. The reaction mixture was quenched by addition H_2O (20.0 mL) and extracted with EtOAc (20.0 mL x 3). The combined organic layers were washed with brine (10.0 mL), dried over sodium sulfate, filtered and concentrated under vacuum. The residue was purified by HPLC using a 10-micron Kromasil C18 column 100 mm X 40 mm. The mobile phase was composed of water (0.1% TFA) and ACN, gradient of ACN from 32%-54% over 10 min. The collected fractions were concentrated by freeze-drying. The product was dissolved in a solution of aqueous HCl (0.5%, 10.0 mL) and MeCN (2.00

mL), then the liquor was concentrated again by freeze-drying. Compound **NA-10** (67.8 mg, 158 μmol, 45.2% yield, 93.7% purity) was obtained as a white solid.

^{1}H NMR: $CDCl_3$ 400MHz, δ: 7.84 (s, 1H), 7.77 (d, J = 6.4 Hz, 1H), 7.48 (s, 1H), 7.31 - 7.37 (m, 2H), 7.21 - 7.25 (m, 1H), 7.18 (s, 1H), 3.86 (m, J = 6.2 Hz, 2H), 2.57 (m, J = 7.1 Hz, 2H), 2.19 (s, 3H), 1.85 - 1.93 (m, 3H), 1.27 (s, 3H). **LCMS:** ($M+H^+$): 402.15, calculated 401.17.

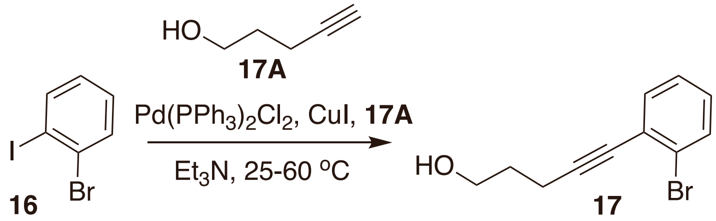

5-(2-bromophenyl)pent-4-yn-1-ol (17): A mixture of compound **16** (2.50 g, 8.84 mmol, 1.14 mL), CuI (100 mg, 530 μmol) and $Pd(PPh_3)_2Cl_2$ (186 mg, 265 μmol) in Et_3N (20.0 mL) was stirred under N_2 at 25°C for 30 min. Then compound **17A** (892 mg, 10.6 mmol) was added and the mixture was stirred at 60°C for 3 h under Ar atmosphere. TLC (petroleum ether/ethyl acetate = 5/1, compound **16** R_f = 0.7, product R_f = 0.2) indicated compound **16** was consumed completely and one main spot formed. The reaction mixture was quenched by addition H_2O (20.0 mL) and extracted with EtOAc (20.0 mL x 3). The combined organic layers were washed with brine (20.0 mL), dried over sodium sulfate, filtered and concentrated under vacuum. The residue was purified by column chromatography (SiO_2, petroleum ether/ethyl acetate = 10/1 to 0/1). The combined organic layers were concentrated under vacuum. Compound **17** (1.71 g, 7.15 mmol, 80.9% yield) was obtained as a white solid.

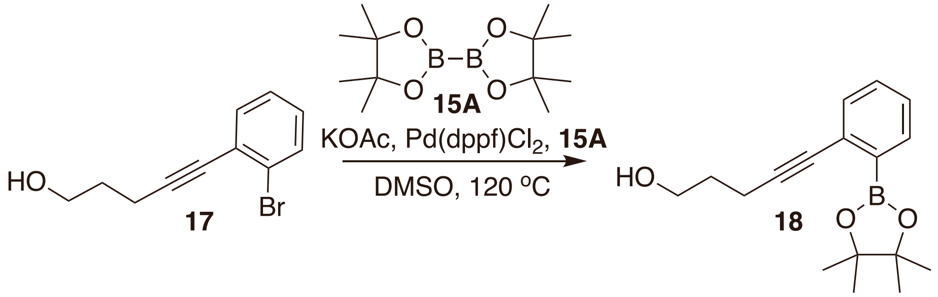

5-(2-(4,4,5,5-tetramethyl-1,3,2-dioxaborolan-2-yl)phenyl)pent-4-yn-1-ol (18): A mixture of compound **17** (1.00 g, 4.18 mmol), KOAc (821 mg, 8.36 mmol), and compound **15A** (1.27 g, 5.02 mmol) in DMSO (10.0 mL) was degassed and purged with N_2 for 3 times, then Pd(dppf)Cl_2 (306 mg, 418 µmol) was added and the mixture was stirred at 120°C for 18 h under N_2 atmosphere. TLC (petroleum ether/ethyl acetate = 2/1, compound **17** $R_f = 0.35$, product $R_f = 0.3$) and HPLC analysis indicated that compound **17** was consumed completely and one main spot formed. The reaction mixture was quenched by addition H_2O (50.0 mL) and extracted with EtOAc (50.0 mL x 3). The combined organic layers were washed with brine (30.0 mL), dried over sodium sulfate, filtered and concentrated under vacuum. The residue was purified by column chromatography (SiO_2, petroleum ether/ethyl acetate = 8/1 to 0/1). The combined organic layers were concentrated under vacuum. Compound **18** (750 mg, 2.62 mmol, 62.7% yield) was obtained as a yellow oil.

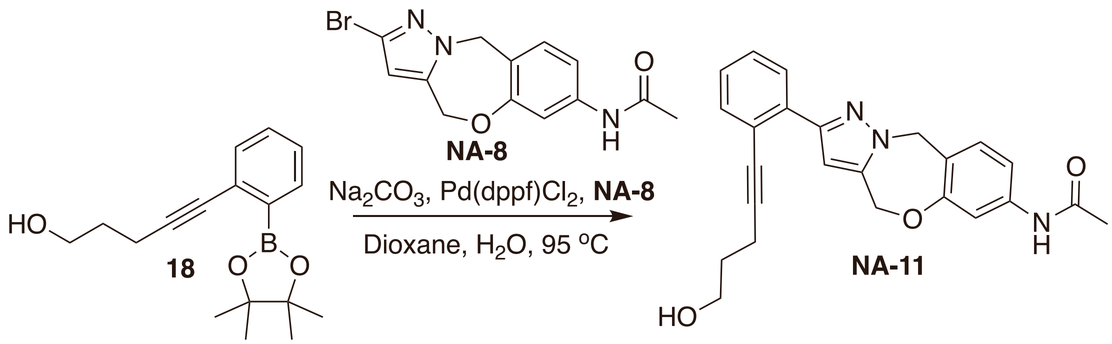

***N*-(2-(2-(5-hydroxypent-1-yn-1-yl)phenyl)-4*H*,10*H*-benzo[*f*]pyrazolo[5,1-*c*][1,4]oxazepin-7-yl)acetamide (NA-11):** Compound **NA-8** (113 mg, 349 µmol), compound **18** (200 mg, 699 µmol), Pd(dppf)Cl_2 (25.6 mg, 34.9 µmol) and Na_2CO_3 (74.1 mg, 699 µmol) were suspended in H_2O (1.00 mL) and dioxane (5.00 mL) and heated to 95°C for 16 h under N_2 atmosphere. TLC (petroleum ether/ethyl acetate = 1/1, compound 18 $R_f = 0.30$, product $R_f = 0.20$) and LCMS analysis indicated that **NA-8** was consumed completely and one main spot formed. The reaction mixture was quenched by addition H_2O (10.0 mL) and extracted with EtOAc (10.0 mL x 3). The combined organic layers were washed with brine (10.0 mL), dried over sodium sulfate, filtered and concentrated under vacuum. The residue was purified by HPLC using a 10-micron Kromasil C18 column 100 mm X 40 mm. The mobile phase was composed of water (0.1% TFA) and ACN, gradient of ACN from 32%-54% over 10 min. The collected fractions were concentrated by freeze-drying. The product was dissolved in a solution of aqueous HCl (0.5%, 10.0 mL) and MeCN (2.00

mL), then the liquor was concentrated again by freeze-drying. Compound **NA-11** (36.5 mg, 87.9 µmol, 25.1% yield, 96.6% purity) was obtained as a white solid.

^{1}H NMR: $CDCl_3$ 400MHz, δ: 7.82 (d, *J* = 7.82 Hz, 1H), 7.46 - 7.51 (m, 2H), 7.29 - 7.43 (m, 3H), 7.21 - 7.25 (m, 1H), 7.10 (d, *J* = 8.80 Hz, 1H), 6.87 (s, 1H), 5.67 (s, 2H), 5.30 (s, 2H), 3.77 (m, *J* = 5.9 Hz, 2H), 2.57 (m, *J* = 6.8 Hz, 2H), 2.18 (s, 3H), 1.84 (m, *J* = 6.4 Hz, 2H), 1.26 (s, 2H).
LCMS: ($M+H^+$): 402.1, calculated 401.17.

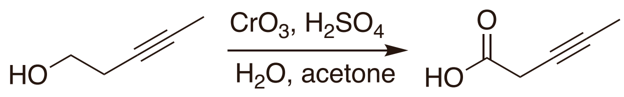

19 20

Pent-3-ynoic acid (20): To a solution of compound **19** (3.80 g, 45.2 mmol) in ACETONE (38.0 mL) was added CrO_3 (9.03 g, 90.4 mmol), H_2SO_4 (31.0 g, 316 mmol) and H_2O (22.8 mL). The mixture was stirred at 17°C for 2 h. TLC (petroleum ether/ethyl acetate = 1/1, compound **19** R_f = 0.46, product R_f = 0.28) indicated compound **19** was consumed completely. The reaction was quenched by addition of 8.00 mL of isopropanol at 15°C. Then the suspension was filtered through a pad of Celite and the filter cake was washed with EtOAc (8.00 mL x 2). The reaction mixture was poured onto ice-water (8.00 mL) and extracted with ethyl acetate (9.00 mL x 3). The combined organic phase was dried with anhydrous Na_2SO_4, filtered and concentrated in vacuum. Compound **20** (3.05 g, 31.1 mmol, 68.8% yield) was obtained as a yellow solid.

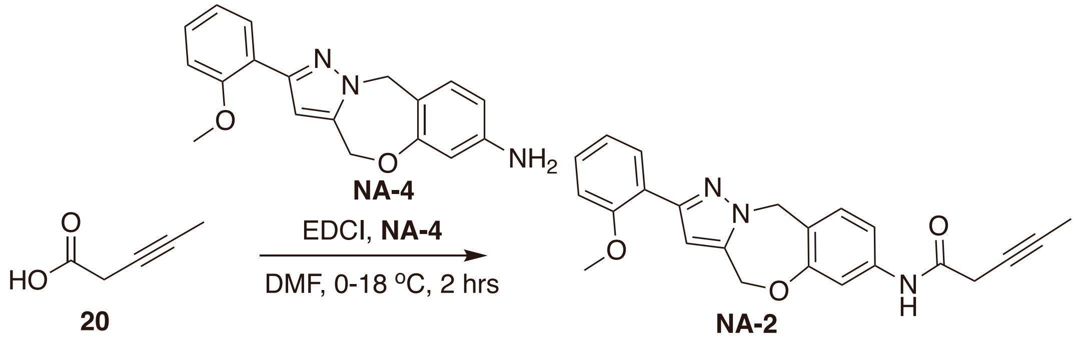

20 NA-2

***N*-(2-(2-methoxyphenyl)-4*H*,10*H*-benzo[*f*]pyrazolo[5,1-*c*][1,4]oxazepin-7-yl)pent-3-ynamide (NA-2):** To a solution of **NA-4** (300 mg, 976 µmol) and compound **20** (192 mg, 1.95 mmol) in DMF (10.0 mL) was added EDCI (374 mg, 1.95 mmol) at 0°C. The mixture was stirred at 18°C

for 2 h. TLC (petroleum ether/ethyl acetate = 1/1, **NA-4** R_f = 0.35, product R_f = 0.45) and LCMS analysis indicated **NA-4** was consumed completely and a main peak for product was detected. The reaction mixture was quenched by addition H_2O (10.0 mL) and extracted with EtOAc (10.0 mL x 3). The combined organic layers were filtered and concentrated under vacuum. The residue was purified by HPLC using a 5-micron Boston Prime C18 column with 150 mm x 30 mm ID. The mobile phase was composed of water (with 0.05% HCl) and a gradient of ACN from 35%-60% over 10 min. The combined product fractions were concentrated by freeze-drying. Compound **NA-2** (73.6 mg, 187 µmol, 19.1% yield, 98.3% purity) was obtained as a white solid.

^{1}H NMR: $CDCl_3$ 400MHz, δ: 8.36 (s, 1H), 8.17 (d, *J* = 7.0 Hz, 1H), 7.79 (s, 1H), 7.44 (m, *J* = 7.9 Hz, 2H), 7.00 - 7.08 (m, 2H), 6.98 - 7.16 (m, 1H), 6.81 (s, 1H), 6.11 (s, 2H), 5.35 (s, 2H), 3.98 (s, 3H), 3.34 (d, *J* = 2.19 Hz, 2H), 1.95 (m, *J* = 2.41 Hz, 3H). **LCMS:** ($M+H^+$): 388.15, calculated 387.16.

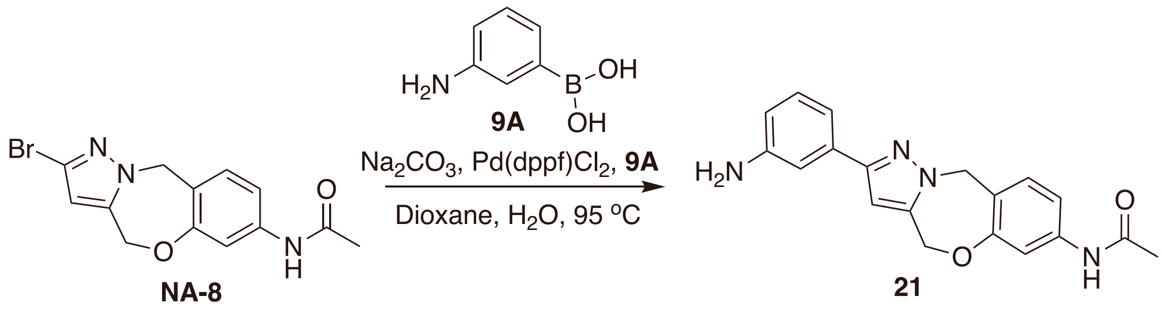

***N*-(2-(3-aminophenyl)-4*H*,10*H*-benzo[*f*]pyrazolo[5,1-*c*][1,4]oxazepin-7-yl)acetamide (21):** Compound **NA-8** (500 mg, 1.55 mmol), compound **9A** (425 mg, 3.10 mmol), Pd(dppf)Cl_2 (114 mg, 155 µmol) and Na_2CO_3 (329 mg, 3.10 mmol) were suspended in H_2O (5.00 mL) and dioxane (25.0 mL) and heated to 95°C for 16 h under N_2 atmosphere. TLC (petroleum ether/ethyl acetate = 1/1, **NA-8** R_f= 0.30, product R_f = 0.25) indicated **NA-8** was consumed completely and one main spot formed. The reaction mixture was quenched by addition H_2O (20.0 mL) and extracted with EtOAc (20.0 mL x 3). The combined organic layers were washed with brine (10.0 mL), dried over sodium sulfate, filtered and concentrated under vacuum. The residue was purified by column chromatography (SiO_2, petroleum ether/ethyl acetate = 20/1 to 0/1). The combined organic layers were concentrated under vacuum. Compound **21** (250 mg, 748 µmol, 48.2% yield) was obtained as a yellow solid.

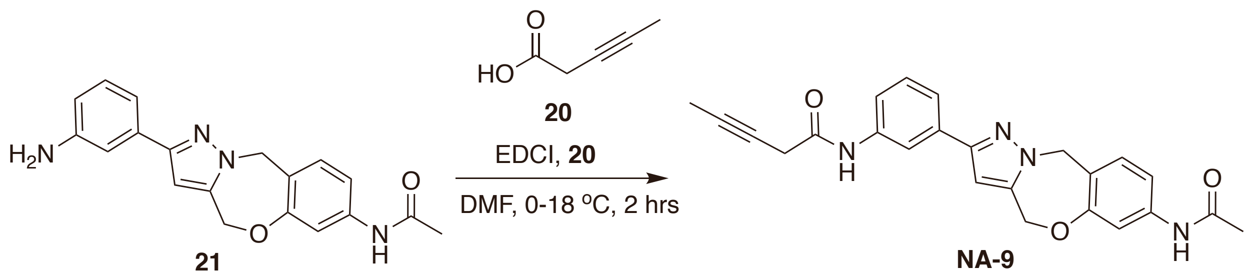

***N*-(3-(7-acetamido-4*H*,10*H*-benzo[*f*]pyrazolo[5,1-*c*][1,4]oxazepin-2-yl)phenyl)pent-3-ynamide (NA-9):** To a solution of compound **20** (117 mg, 1.20 mmol) and compound **21** (200 mg, 598 µmol) in DMF (10.0 mL) was added EDCl (229 mg, 1.20 mmol) at 0°C. The mixture was stirred at 18°C for 2 h. TLC (petroleum ether/ethyl acetate = 0/1, compound **21** R_f = 0.30, product R_f = 0.43) and LCMS analysis indicated **NA-4** was consumed completely and a main peak for product was detected. The reaction mixture was quenched by addition H_2O (10.0 mL) and extracted with EtOAc (10.0 mL x 3). The combined organic layers were filtered and concentrated under vacuum. The residue was purified by HPLC using a 5-micron Boston Prime C18 column with 150 mm x 30 mm ID. The mobile phase was composed of water (with 0.05% HCl) and a gradient of ACN from 25%-50% over 10 min. The combined product fractions were concentrated by freeze-drying. Compound **NA-9** (148 mg, 353 µmol, 59.0% yield, 98.4% purity) was obtained as a white solid.

^{1}H NMR: DMSO-d6 400MHz, δ: 10.06 (s, 1H), 9.98 (s, 1H), 8.05 (s, 1H), 7.49 (d, *J* = 7.45 Hz, 1H), 7.44 (d, *J* = 7.9 Hz, 1H), 7.25 - 7.34 (m, 3H), 7.14 (m, *J* = 8.10 Hz, 1H), 6.71 (s, 1H), 5.57 (s, 2H), 5.35 (s, 2H), 4.19 (s, 7H), 3.25 - 3.29 (m, 2H), 2.52 - 2.54 (m, 1H), 2.02 (s, 3H), 1.80 (m, *J* = 2.64 Hz, 3H). **LCMS:** (M+H$^+$): 415.16, calculated 414.17.

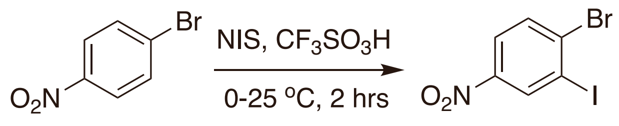

22

23

1-bromo-2-iodo-4-nitrobenzene (23): To a solution of compound **22** (10.0 g, 49.5 mmol) in CF_3SO_3H (22.0 mL) was added portion-wise NIS (11.1 g, 49.5 mmol) at 0°C. After addition, the mixture was stirred at 25°C for 2 h. TLC (petroleum ether, starting material: $R_f = 0.5$, product: $R_f = 0.45$) indicated compound **22** was consumed completely. The reaction mixture was quenched with ice-water (20.0 mL) and extracted with DCM (25.0 mL x 3). The combined organic extracts were washed with aqueous 10% sodium sulfite solution (20.0 mL) and water (10.0 mL), dried over Na_2SO_4, and evaporated under reduced pressure to give compound **23** (11.5 g, 70.9% yield) as a brown solid, which was used into the next step without further purification.

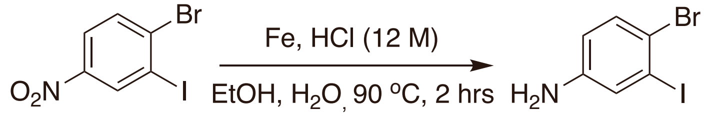

23 **24**

4-bromo-3-iodoaniline (24): To a solution of compound **23** (11.6 g, 35.4 mmol) in EtOH (15.0 mL) and H_2O (15.0 mL) was added drop-wise HCl (12 M, 4.42 mL) and Fe (5.93 g, 106 mmol) at 90°C. The resulting mixture was stirred at 90°C for 2 h. TLC (petroleum ether/ethyl acetate = 3/1, starting material: $R_f = 0.5$, product: $R_f = 0.3$) indicated compound **23** was consumed completely. The residue was poured into water (10.0 mL). The aqueous phase was extracted with ethyl acetate (30.0 mL x 3). The combined organic phase was dried with anhydrous Na_2SO_4, filtered and concentrated under vacuum to give compound **24** (10.5 g, 99.6% yield) as a brown oil, which was used into the next step without further purification.

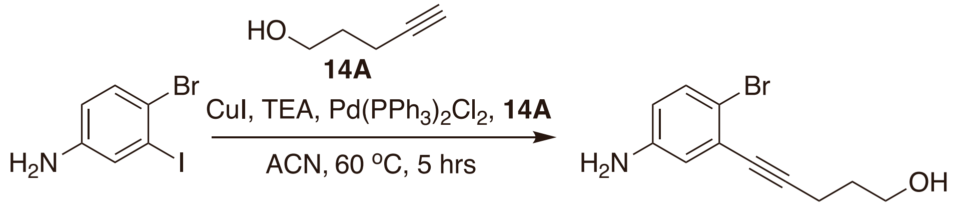

24 **25**

5-(5-amino-2-bromophenyl)pent-4-yn-1-ol (25): To a solution of compound **24** (5.50 g, 18.5 mmol) in ACN (20.0 mL) was added compound **14A** (1.86 g, 22.2 mmol) and TEA (3.74 g, 36.9 mmol, 5.14 mL). The mixture was degassed with Ar, then CuI (70.3 mg, 369 µmol) and

$Pd(PPh_3)_2Cl_2$ (259 mg, 369 umol) were added. The reaction was heated at 60°C for 5 h. TLC (petroleum ether/ethyl acetate = 3/1, starting material: R_f = 0.3, product: R_f = 0.2) indicated compound **24** was consumed completely. The reaction mixture was concentrated under reduced pressure to remove ACN. The residue was diluted with H_2O (10.0 mL) and extracted with EtOAc (10.0 mL x 3), and dried over Na_2SO_4, filtered and concentrated under vacuum. The residue was purified by column chromatography (SiO_2, petroleum ether/ethyl acetate = 10/1 to 2/1). Compound **25** (3.40 g, 72.5% yield) was obtained as a brown solid.

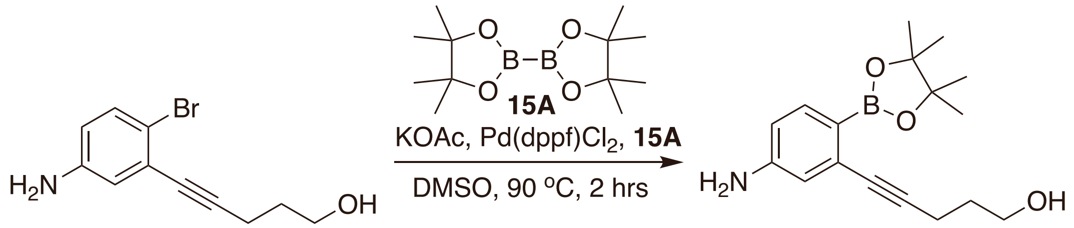

25 **26**

5-(5-amino-2-(4,4,5,5-tetramethyl-1,3,2-dioxaborolan-2-yl)phenyl)pent-4-yn-1-ol (26): A mixture of compound **25** (4.40 g, 17.3 mmol), compound **15A** (8.79 g, 34.6 mmol) and KOAc (5.10 g, 51.9 mmol) in DMSO (25.0 mL) was degassed and purged with N_2 for 3 times. $Pd(dppf)Cl_2 \cdot CH_2Cl_2$ (1.41 g, 1.73 mmol) was added and then the mixture was stirred at 90°C for 2 h under N_2 atmosphere. TLC (petroleum ether/ethyl acetate = 1/1, starting material: R_f = 0.4, product: R_f = 0.45) indicated compound **25** was consumed completely. LCMS analysis showed compound **25** was consumed completely and ~21.6 % of desired mass was detected. The residue was diluted with H_2O (20.0 mL) and extracted with EtOAc (20.0 mL x 3), and dried over Na_2SO_4, filtered and concentrated under vacuum. The residue was purified by column chromatography (SiO_2, petroleum ether/ethyl acetate = 10/1 to 1/1) to give compound **26** (660 mg, 12.7% yield) as a brown oil.

^{1}H NMR: $CDCl_3$-400MHz, δ: 7.50 (d, *J* = 8.0 Hz, 1 H), 6.65 (d, *J* = 2.4 Hz, 1 H), 6.50 (dd, *J* = 8.0, 2.4 Hz, 1 H), 4.05 (q, *J* = 7.2 Hz, 1 H), 3.80 (t, *J* = 6.0 Hz, 2 H), 2.50 (t, *J* = 6.4 Hz, 2 H), 1.97 (s, 1 H), 1.79 (q, *J* = 6.4 Hz, 3 H), 1.47 (s, 3 H), 1.26 (s, 12 H).

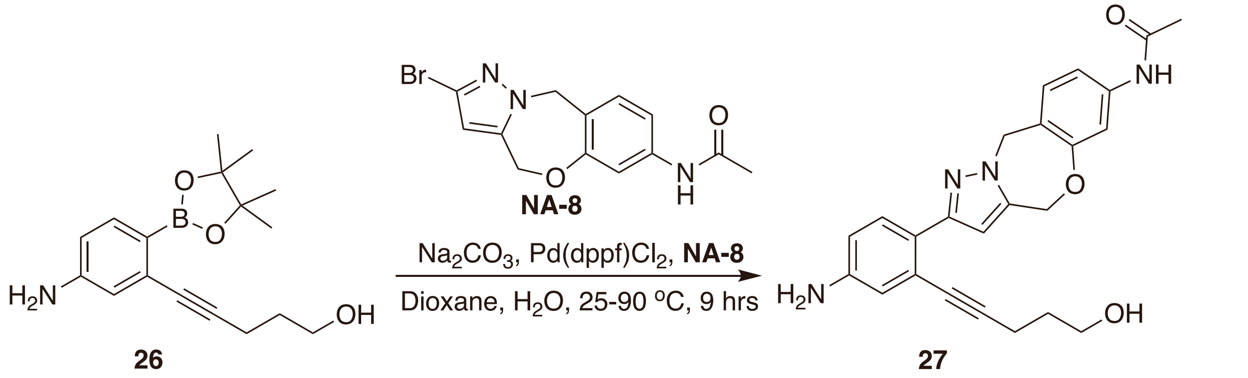

***N*-(2-(4-amino-2-(5-hydroxypent-1-yn-1-yl)phenyl)-4*H*,10*H*-benzo[*f*]pyrazolo[5,1-*c*][1,4]oxazepin-7-yl)acetamide (27):** A mixture of compound **26** (467 mg, 1.55 mmol), compound **NA-8** (250 mg, 776 μmol) and Na_2CO_3 (165 mg, 1.55 mmol) in H_2O (2.50 mL) and dioxane (12.5 mL) was degassed and purged with N_2 for 3 times, then Pd(dppf)Cl_2 (56.8 mg, 77.6 μmol) was added and the mixture was stirred at 90°C for 9 h under N_2 atmosphere. TLC (ethyl acetate, starting material: R_f = 0.7, product: R_f = 0.3) and LCMS analysis indicated compound **26** was consumed completely and ~22.4 % of desired product was detected. The reaction mixture was poured into water (10.0 mL) and extracted with ethyl acetate (15.0 mL x 3), dried over Na_2SO_4, filtered and concentrated under vacuum. The crude product was purified by column chromatography (SiO_2, petroleum ether/ethyl acetate = 30/1 to 1/3). Compound **27** (100 mg, 19.3 % yield) was obtained as a brown solid.

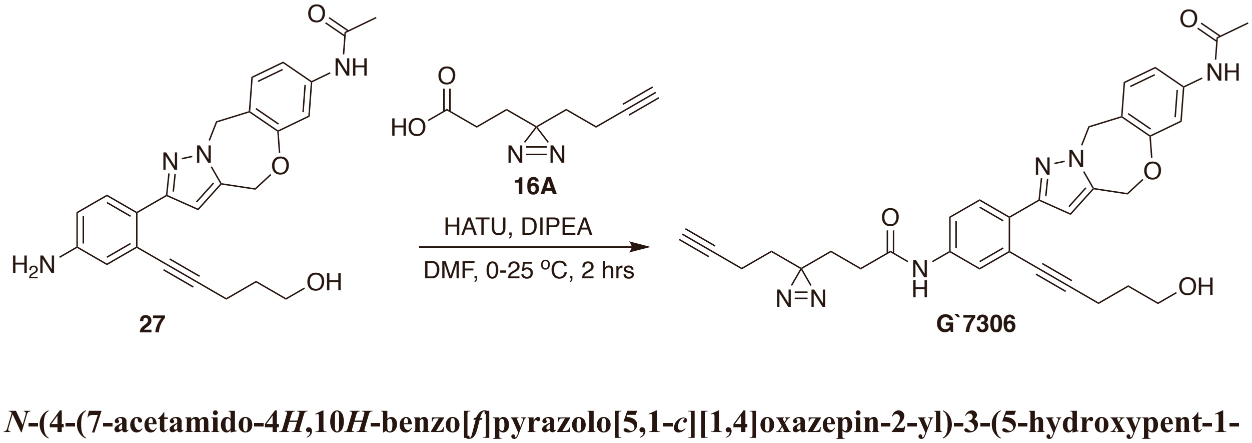

***N*-(4-(7-acetamido-4*H*,10*H*-benzo[*f*]pyrazolo[5,1-*c*][1,4]oxazepin-2-yl)-3-(5-hydroxypent-1-yn-1-yl)phenyl)-3-(3-(but-3-yn-1-yl)-3*H*-diazirin-3-yl)propenamide (G`7306):** To a solution of compound **27** (38.0 mg, 229 μmol) in DMF (1.50 mL) was added HATU (130 mg, 343 μmol), DIPEA (59.1 mg, 457 μmol, 79.7 μL) and compound **16A** (100 mg, 240 μmol) at 0°C. The mixture

was stirred at 25°C for 2 h. LCMS analysis showed compound **27** was consumed completely and ~31.4% of desired product was detected. The residue was purified by HPLC using a Waters Xbridge 150 mm X 25 mm and 5 µm particle size. The mobile phase was composed of 10 mM NH_4HCO_3 (aq) and ACN, gradient of ACN from 25%-55% over 20 min. The collected fractions were concentrated by freeze-drying. Compound **G`7306** (36.7 mg, 28.4% yield, 100% purity) was obtained as a light yellow solid.

^{1}H NMR: $CDCl_3$-400MHz, δ: 7.73 (d, J = 8.4 Hz, 1 H), 7.66 (s, 1 H), 7.43 (d, J = 9.2 Hz, 1 H), 7.44 (s, 1 H), 7.16 - 7.22 (m, 2 H), 7.07 - 7.15 (m, 2 H), 6.81 (s, 1 H), 5.52 (s, 2 H), 5.27 (s, 2 H), 3.76 (d, J = 6.0 Hz, 2 H), 2.55 (t, J = 6.8 Hz, 2 H), 2.18 (s, 3 H), 2.13 (t, J = 6.8 Hz, 2 H), 2.03 - 2.08 (m, 2 H), 2.00 (s, 1 H), 1.92 - 1.98 (m, 2 H), 1.83 (t, J = 6.4 Hz, 2 H), 1.70 (t, J = 7.6 Hz, 2 H). **LCMS:** ($M+H^+$): 563.2, calculated 564.25.

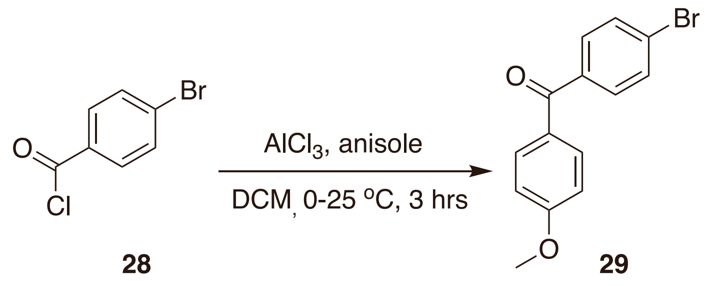

(4-bromophenyl)(4-methoxyphenyl)methanone (29): A solution of anisole (2.00 g, 18.5 mmol, 2.01 mL) in DCM (8.00 mL) was cooled to 0°C. $AlCl_3$ (2.71 g, 20.3 mmol, 1.11 mL) was added in several portions, then a mixture of compound **28** (4.06 g, 18.5 mmol) in DCM (4.00 mL) was added drop wise at 0°C. The resulting mixture was stirred at 25°C for 3 h under N_2. TLC (petroleum ether/ethyl acetate = 5/1, product R_f = 0.6) showed the anisole (R_f = 1.0) was consumed and a main new spot with larger polarity was formed. The mixture was quenched with ice water (30.0 mL), extracted with DCM (20.0 mL x 2) and the organic layers were combined, dried over Na_2SO_4, filtered and concentrated under vacuum. Compound **29** (5.10 g, 94.7% yield) was obtained as an off white solid.

^{1}H NMR: $CDCl_3$ 400MHz, δ: 7.81-7.79 (m, 2 H), 7.65-7.58 (m, 4 H), 7.26 (s, 1 H), 6.99-6.96 (m, 2 H), 3.89 (s, 3 H).

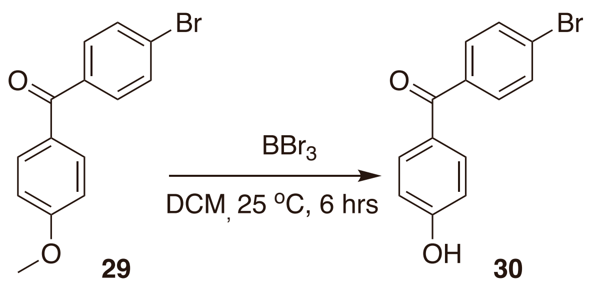

(4-bromophenyl)(4-hydroxyphenyl)methanone (30): A mixture of compound **29** (5.10 g, 17.5 mmol) in dry DCM (30.0 mL) was cooled to -70 °C under N_2. Then BBr_3 (17.6 g, 70.1 mmol, 6.75 mL) was added dropwise at -70°C, after addition, the resulting mixture was stirred at 25°C for 14 h under N_2. TLC (petroleum ether/ethyl acetate = 5/1, product R_f = 0.3) showed compound **29** (R_f = 0.6) was consumed, a main new spot appeared. The mixture was cooled to 0°C, MeOH (50.0 mL) was added drop wise and the resulting mixture was concentrated under vacuum. The residue was purified by column chromatography (SiO_2, petroleum ether/ethyl acetate = 100/1 to 1/1). Compound **30** (5.30 g, crude) was obtained as a yellow solid.

^{1}H NMR: MeOD 400MHz, δ: 7.71-7.67 (m, 4 H), 7.63-7.60 (m, 2 H), 6.91-6.87 (m, 2 H).

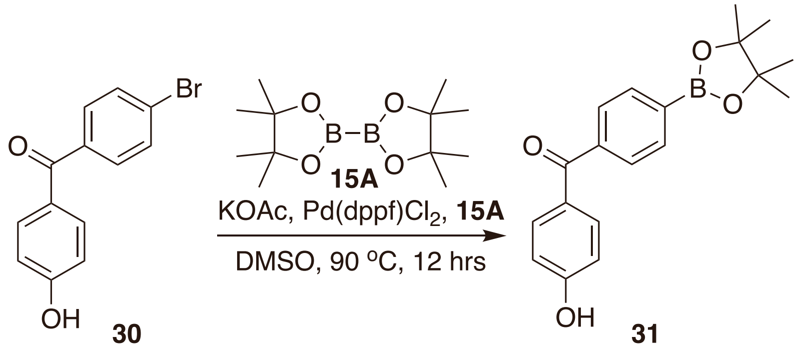

(4-hydroxyphenyl)(4-(4,4,5,5-tetramethyl-1,3,2-dioxaborolan-2-yl)phenyl)methanone (31): To a solution of compound **30** (4.20 g, 15.2 mmol) in dioxane (63.0 mL) was added compound **15A** (5.00 g, 19.7 mmol), KOAc (2.97 g, 30.3 mmol) and Pd(dppf)Cl_2 (1.11 g, 1.50 mmol) at 25°C. The reaction mixture was degassed and purged with N_2 for 3 times, and then the mixture was stirred at 90°C for 12 h under N_2 atmosphere. TLC (petroleum ether/ethyl acetate = 3/1, product R_f = 0.5) and LCMS analysis showed compound **30** (R_f = 0.55) was consumed and a new peak of the desired product was formed. The crude product was filtered to remove the insoluble

and the filtrate was concentrated under vacuum. The residue was purified by column chromatography (SiO_2, petroleum ether/ethyl acetate = 50/1 to 1/1). Compound **31** (3.38 g, 68.8% yield) was obtained as a white solid.

^{1}H NMR: DMSO 400MHz, δ: 10.46 (s, 1 H), 7.81 (d, *J* = 8.0 Hz, 2 H), 7.66 - 7.63 (m, 4 H), 6.89 (d, *J* = 8.8 Hz, 2 H), 1.32 (s, 1 H).

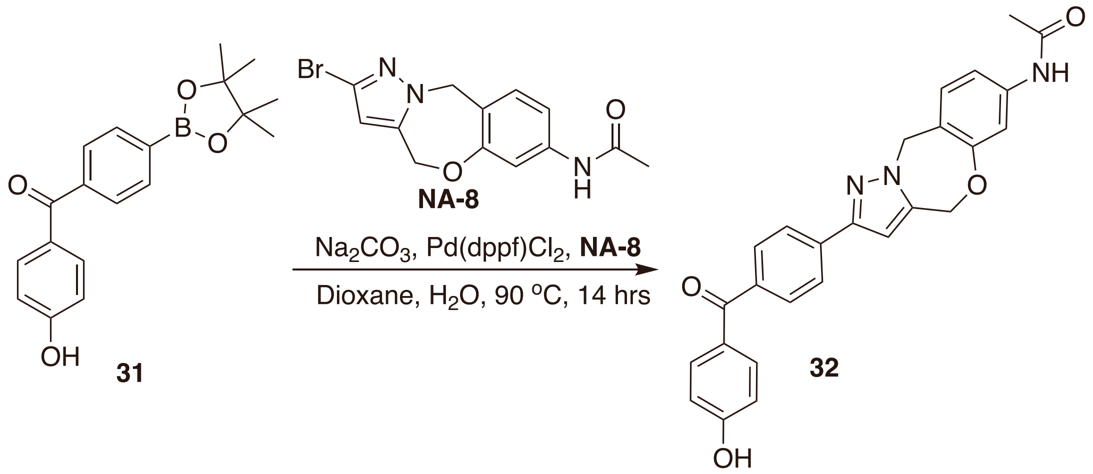

***N*-(2-(4-(4-hydroxybenzoyl)phenyl)-4*H*,10*H*-benzo[*f*]pyrazolo[5,1-*c*][1,4]oxazepin-7-yl)acetamide (32):** To a solution of compound **31** (403 mg, 1.24 mmol) in dioxane (10.0 mL) and H_2O (2.00 mL) was added compound **NA-8** (200 mg, 621 μmol), Pd(dppf)Cl_2 (45.4 mg, 62.1 μmol) and Na_2CO_3 (132 mg, 1.24 mmol) at 25°C, the reaction mixture was degassed and purged with N_2 for 3 times and then the mixture was stirred at 90°C for 14 h under N_2 atmosphere. TLC (petroleum ether/ethyl acetate = 1/2, product R_f = 0.3) showed compound **6** (R_f = 0.45) was consumed, several new spots formed. The reaction mixture was quenched by addition H_2O (10.0 mL), extracted with EtOAc (25.0 mL x 5), the organic layers were combined, dried over sodium sulfate, filtered and concentrated under vacuum. The crude product was triturated with MTBE (2.50 mL) at 25°C for 15 mins to give compound **32** (100 mg, 36.7% yield) as a gray solid.

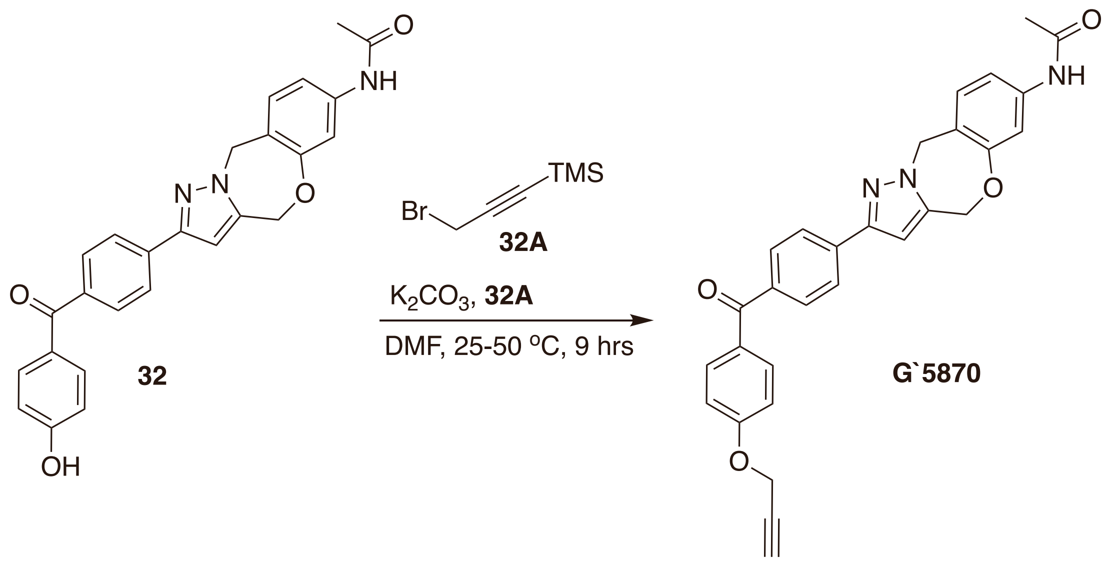

***N*-(2-(4-(4-(prop-2-yn-1-yloxy)benzoyl)phenyl)-4*H*,10*H*-benzo[*f*]pyrazolo[5,1-*c*][1,4]oxazepin-7-yl)acetamide (G`5870):** To a solution of compound **32** (100 mg, 228 µmol) in DMF (6.00 mL) was added compound **32A** (52.2 mg, 273 µmol, 44.6 µL) and K_2CO_3 (94.4 mg, 683 µmol) at 25°C. The reaction mixture was degassed and purged with N_2 for 3 times. The reaction was stirred at 25°C for 2 h, then at 50°C for 7 h under N_2 atmosphere. TLC (petroleum ether/ethyl acetate = 1/2, product R_f = 0.35) and HPLC analysis showed compound **32** was consumed and a main peak of the desired product was detected. The mixture was cooled to 25°C and filtered. The filtrate was purified by HPLC using a 10-micron Waters Xbridge Prep OBD C18 column with ID of 150 mm X 40 mm. The mobile phase was composed of water (with 10 mM NH_4HCO_3) and a gradient of ACN from 35% - 60% over 8 min. Compound **G`5870** (60.7 mg, 55.0% yield, 98.5% purity) was obtained as a white solid.

^{1}H NMR: DMSO 400MHz, δ: 9.96 (s, 1 H), 7.95 (d, *J* = 8.4 Hz, 2 H), 7.79 - 7.74 (m, 4 H), 7.30 (d, *J* = 8.0 Hz, 2 H), 7.15 (d, *J* = 8.4 Hz, 3 H), 6.92 (s, 1 H), 5.62 (s, 2 H), 5.38 (s, 2 H), 4.94 - 4.93 (m, 2 H), 3.65 (s, 1 H), 2.02 (s, 3 H). **LCMS:** (M+H): 478.2, calculated 477.17.

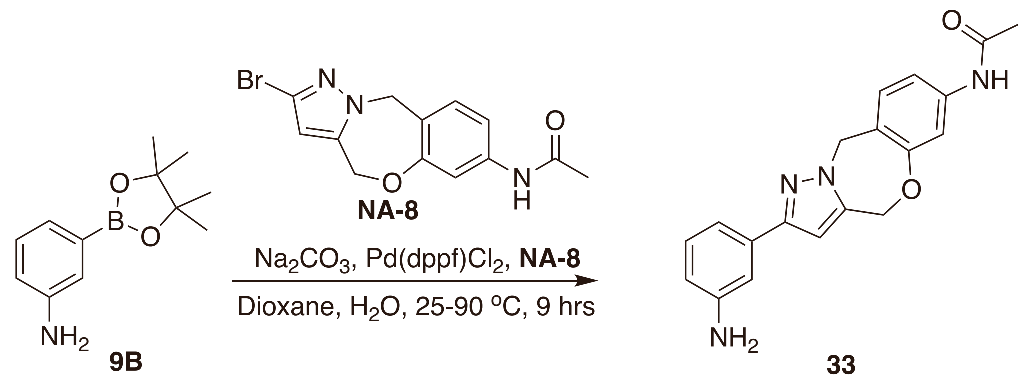

***N*-(2-(3-aminophenyl)-4*H*,10*H*-benzo[*f*]pyrazolo[5,1-*c*][1,4]oxazepin-7-yl)acetamide (33):** To a solution of compound **NA-8** (400 mg, 1.24 mmol) in dioxane (2.00 mL) and H_2O (0.50 mL) was added compound **9B** (544 mg, 2.48 mmol*)*, Pd(dppf)Cl_2 (90.9 mg, 124 umol) and Na_2CO_3 (263 mg, 2.48 mmol) at 25°C, the reaction mixture was degassed and purged with N_2 for 3 times, and then stirred at 95°C for 12 h under N_2 atmosphere. LCMS analysis showed compound **NA-8** was consumed completely and one main peak of the desired product was detected. The reaction mixture was diluted with H_2O (7.00 mL) and extracted with ethyl acetate (5.00 mL x 3). The combined organic layers were washed with brine (5.00 mL x 2), dried over Na_2SO_4, filtered and concentrated under vacuum. The residue was purified by column chromatography (SiO_2, petroleum ether/ethyl acetate=10/1 to 0/1). Compound **33** (250 mg, 60.2% yield) was obtained as a light yellow solid. **^{1}H NMR:** $CDCl_3$-400MHz, δ: 7.18 (s, 2H), 7.06 (m, 5H), 6.55 (d, *J* = 7.2 Hz, 1H), 6.34 (s, 1H), 5.40 (s, 2H), 5.17(s, 2H), 2.09 (s, 3H). **LCMS:** (M+H$^+$): 335.1, calculated 334.14.

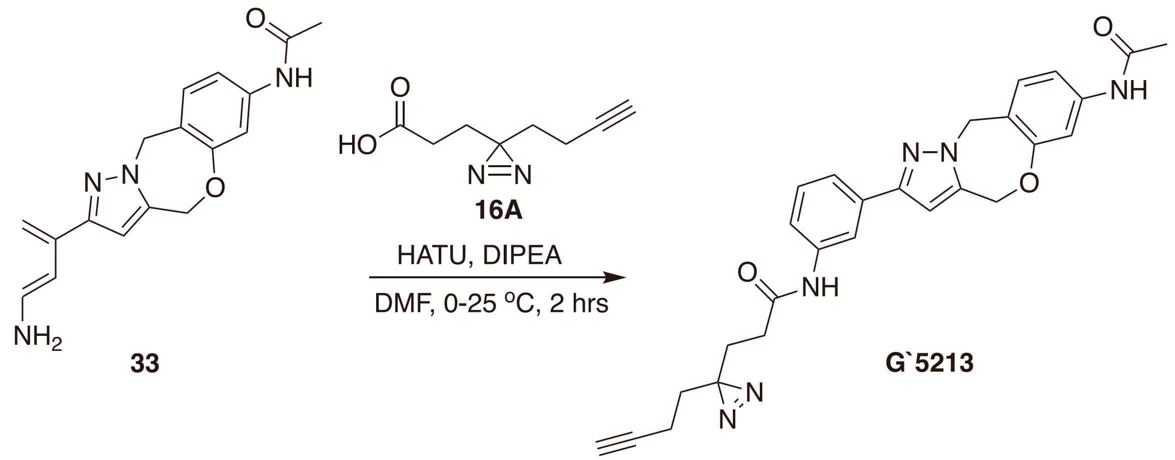

***N*-(3-(7-acetamido-4*H*,10*H*-benzo[*f*]pyrazolo[5,1-*c*][1,4]oxazepin-2-yl)phenyl)-3-(3-(but-3-yn-1-yl)-3*H*-diazirin-3-yl)propenamide (G`5213):** To a solution of compound **16A** (70.0 mg, 421 µmol) in DMF (2.00 mL) was added HATU (240 mg, 632 µmol), DIPEA (109 mg, 842 µmol, 147 µL) and compound **33** (148 mg, 442 µmol) at 0°C. The mixture was stirred at 25°C for 2 h. LCMS and HPLC analysis showed compound **33** was consumed completely and one main peak of the desired product was detected. The crude product was used directly for the purification without work-up. The residue was purified by HPLC using a 10-micron Waters Xbridge Prep OBD C18 column with ID of 150 mm X 40 mm. The mobile phase was composed of water (with 10 mM NH_4HCO_3) and a gradient of ACN from 20% - 50% over 10 min. Compound **G`5213** (51.7 mg, 26.6% yield, 99.1% purity) was obtained as a white solid.

^{1}H NMR: $CDCl_3$-400MHz, δ: 7.82 (s, 1H), 7.52 (m, 2H), 7.35 (d, *J* = 7.6 Hz, 2H), 7.19 (d, *J* = 8.4 Hz, 1H), 7.13 (s, 2H), 6.46 (s, 1H), 5.48 (s, 2H), 5.25 (s, 2H), 2.13 (m, 3H), 2.00 (m, 7H), 1.69 (t, *J* = 7.2 Hz, 2H). **LCMS:** (M+H$^+$): 483.3, calculated 482.21.

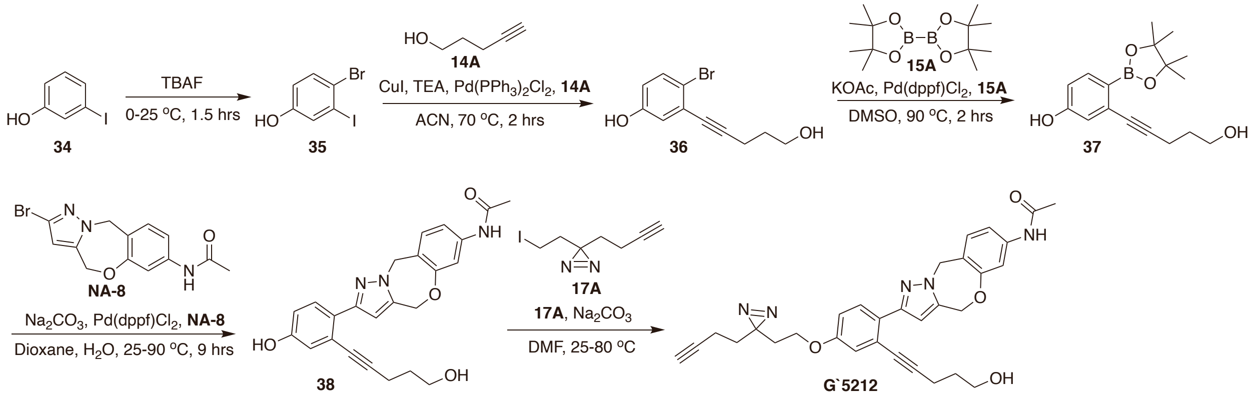

4-bromo-3-iodophenol (35): To a solution of compound **34** (20.0 g, 90.9 mmol) was added Br_2 (18.2 g, 114 mmol, 5.86 mL) in AcOH (120 mL) at 15°C. The mixture was stirred at 25°C for 2 h. HPLC analysis indicated compound **34** was consumed completely. The reaction mixture was stirred at 25°C and a saturated solution of $Na_2S_2O_3$ (100 mL) was added, then it was neutralized with $NaHCO_3$ (40.0 mL) and the layers were separated. The aqueous layer was extracted with DCM (100 mL x 2). The combined organic layers were washed with water, dried over Na_2SO_4 and concentrated under vacuum. The residue was purified by column chromatography (SiO_2, petroleum ether/ethyl acetate = 10/1 to 1/1). Compound **35** (23.0 g, 84.6% yield) was obtained as a white solid.

^{1}H NMR: $CDCl_3$-400MHz, δ: 7.42 (d, *J* = 8.8 Hz,1H), 7.37 (t, *J* = 2.8 Hz, 1H), 6.73-6.70 (m, 1H).

4-bromo-3-(5-hydroxypent-1-yn-1-yl)phenol (36): To a solution of compound **35** (23.0 g, 77.0 mmol) in ACN (115 mL) was added compound **14A** (9.06 g, 108 mmol) and TEA (15.6 g, 154 mmol, 21.4 mL). The mixture was degassed with N_2, then $Pd(PPh_3)_2Cl_2$ (1.08 g, 1.54 mmol) and CuI (586 mg, 3.08 mmol) were added. The reaction was heated at 70°C for 2 h. TLC (ethyl acetate, product: R_f = 0.39) indicated compound **35** was consumed completely and one new spot formed. The reaction mixture was diluted with H_2O (70.0 mL) and extracted with ethyl acetate (25.0 mL x 3). The combined organic layers were washed with brine (20.0 mL x 2), dried over Na_2SO_4**,** filtered and concentrated under vacuum. The residue was purified by column chromatography (SiO_2, petroleum ether/ethyl acetate = 5/1 to 0/1). Compound **36** (16.0 g, 81.5% yield) was obtained as a white solid.
^{1}H NMR: $CDCl_3$-400MHz, δ: 7.35 (d, *J* = 8.8 Hz, 1H), 6.9 (d, *J* = 2.8 Hz, 1H), 6.67-6.64 (m, 1H), 3.89 (t, *J* = 6.4 Hz, 2H), 2.59 (t, *J* = 6.8 Hz, 2H), 1.93-1.86 (m, 2H).

3-(5-hydroxypent-1-yn-1-yl)-4-(4,4,5,5-tetramethyl-1,3,2-dioxaborolan-2-yl)phenol (37): To a solution of compound **36** (16.0 g, 62.7 mmol) in DMSO (160 mL) was added compound **15A** (31.9 g, 125 mmol), $Pd(dppf)Cl_2•CH_2Cl_2$ (5.12 g, 6.27 mmol) and KOAc (18.5 g, 188 mmol) at 25°C. The reaction mixture was degassed and purged with N_2 for 3 times, and then the mixture was stirred at 90°C for 2 h under N_2 atmosphere. LCMS analysis showed compound **36** was consumed completely and one peak of the desired product was detected. The reaction mixture was diluted with H_2O (70.0 mL) and extracted with ethyl acetate (25.0 mL x 3). The combined organic layers were washed with brine (20.0 mL x 2), dried over Na_2SO_4**,** filtered and concentrated under vacuum. The residue was purified by column chromatography (SiO_2, petroleum ether/ethyl acetate = 10/1 to 0/1). Compound **37** (10.4 g, 49.8% yield, 90.8% purity) was obtained as a brown oil.
^{1}H NMR: DMSO- d_6 400MHz, δ: 7.45 (d, *J* = 8.0 Hz, 1H), 6.74-6.68 (m, 2H), 3.58-3.54 (m, 2H), 2.43 (t, *J* = 6.8 Hz, 2H), 1.73-1.65 (m, 2H), 1.08 (s, 12H). **LCMS:** ($M+H^+$): 303.1, Calculated 302.17.

***N*-(2-(4-hydroxy-2-(5-hydroxypent-1-yn-1-yl)phenyl)-4*H*,10*H*-benzo[*f*]pyrazolo[5,1-*c*][1,4]oxazepin-7-yl)acetamide (38):** To a solution of compound **37** (703 mg, 2.33 mmol) in

dioxane (25.0 mL) and H_2O (5.00 mL) was added compound **NA-8** (500 mg, 1.55 mmol), Na_2CO_3 (329 mg, 3.10 mmol) and Pd(dppf)Cl_2 (114 mg, 155 μmol) at 25°C. The reaction mixture was degassed and purged with N_2 for 3 times, and then the mixture was stirred at 90°C for 12 h under N_2 atmosphere. LCMS analysis showed compound **37** was consumed completely and one main peak of the desired product was detected. The reaction mixture was diluted with H_2O (7.00 mL) and extracted with EtOAc (5.00 mL x 3). The combined organic layers were washed with brine (5.00 mL x 2). The combined organic layers were dried over Na_2SO_4, filtered and concentrated under vacuum. The residue was purified by column chromatography (SiO_2, petroleum ether/ethyl acetate = 5/1 to 0/1). Compound **38** (160 mg, 24.6% yield) was obtained as a light yellow solid.

***N*-(2-(4-(2-(3-(but-3-yn-1-yl)-3*H*-diazirin-3-yl)ethoxy)-2-(5-hydroxypent-1-yn-1-yl)phenyl)-4*H*,10*H*-benzo[*f*]pyrazolo[5,1-*c*][1,4]oxazepin-7-yl)acetamide (G`5212):** To a solution of compound **38** (150 mg, 359 μmol) in DMF (5.00 mL) was added Na_2CO_3 (76.2 mg, 719 μmol) and compound **17A** (152 mg, 611 μmol) at 25°C. The mixture was stirred at 80°C for 12 h. LCMS and HPLC analysis showed the starting material was consumed completely and one main peak of the desired product was detected. The reaction mixture was concentrated under vacuum and the crude product was used directly for purification without work-up. The residue was purified by HPLC using a 5-micron Welch Xtimate C18 column with ID of 150 mm X 25 mm. The mobile phase was composed of water (with 0.04% HCl) and a gradient of ACN from 40% - 70% over 10 min. Collected fractions were concentrated by freeze-drying. Compound G`5212 (30.0 mg, 14.9% yield, 96.5% purity) was obtained as a brown solid.

^{1}H NMR: $CDCl_3$-400MHz, δ: 7.89-7.84 (m, 2H), 7.66 (s, 1H), 7.24 (s, 1H), 7.09 (t, *J* = 2.8 Hz, 1H), 7.01 (s, 1H), 6.95 (d, *J* = 7.2 Hz, 1H), 6.89 (s, 1H), 5.83 (s, 2H), 5.31 (s, 2H), 3.86 (t, *J* = 6.0 Hz, 2H), 3.77 (t, *J* = 4.8 Hz, 2H), 2.58 (t, *J* = 6.4 Hz, 2H), 2.19 (s, 3H), 2.10-2.06 (m, 3H), 2.01 (t, *J* = 2.4 Hz, 1H), 1.91 (t, *J* = 6.0 Hz, 2H), 1.84 (t, *J* = 5.4 Hz, 2H), 1.75 (t, *J* = 7.6 Hz, 2H). **LCMS:** ($M+H^+$): 538.2, calculated 537.24.

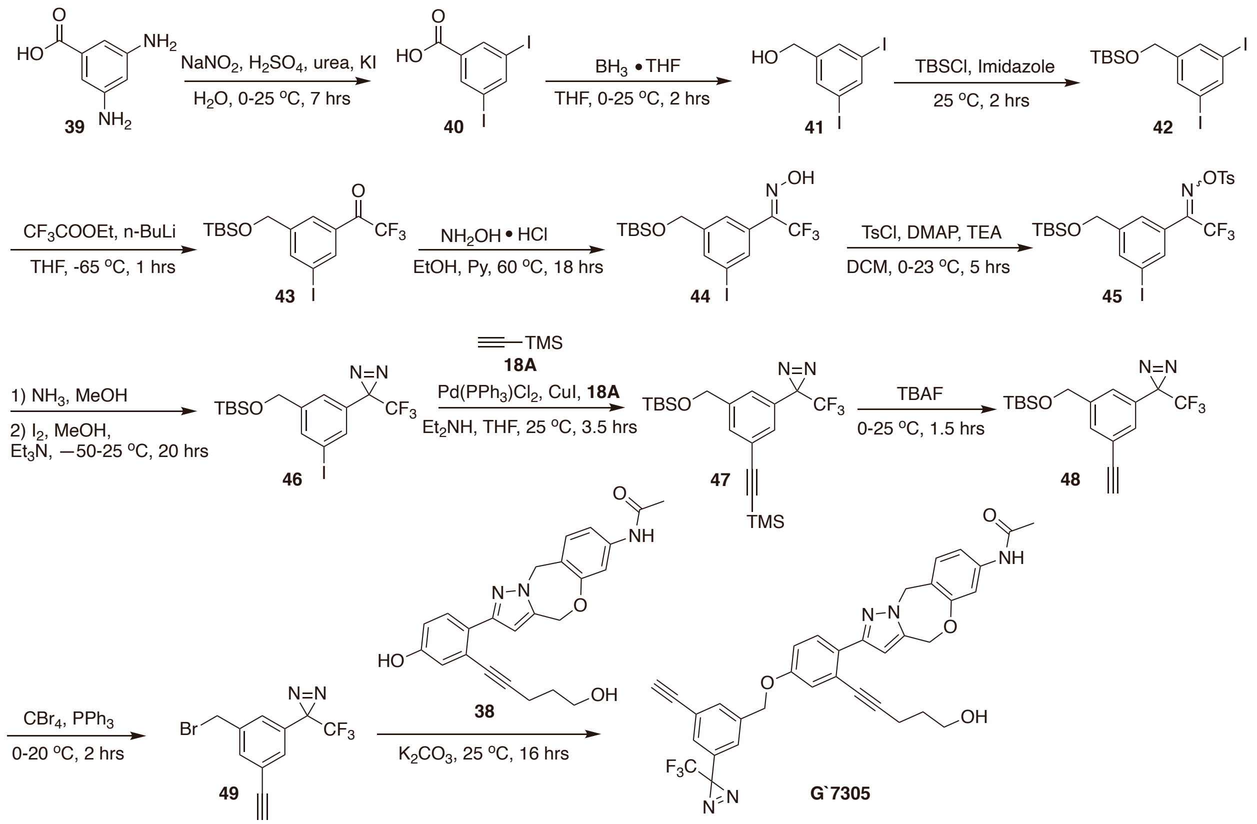

3,5-diiodobenzoic acid (40): To a solution of compound **39** (50.0 g, 329 mmol) in 25% H_2SO_4 (1.16 L) was added drop wise $NaNO_2$ (2.5 M, 276 mL) at 0°C. After addition, the mixture was stirred at this temperature for 2 h. Urea (49.3 g, 821 mmol, 44.1 mL) was added, and then the solution was added slowly to an ice-cooled solution of KI (273 g, 1.64 mol) in H_2O (273 mL). The resulting mixture was stirred at 25°C for 5 h. TLC (petroleum ether/ethyl acetate = 1/1, product R_f = 0.3) indicated compound **39** (R_f = 0.0) was consumed completely and one new spot formed. The reaction mixture was filtered and the filter cake was washed with H_2O (50.0 mL) and dried in vacuum to give a solid residue. This solid was dissolved in THF (1.00 L), and the solution was washed with aqueous $Na_2S_2O_3$ (800 mL) until the I_2 had disappeared. Compound **40** (88.0 g, 71.6% yield) was obtained as a brown solid.

^{1}H NMR: $CDCl_3$ 400MHz, δ: 8.34 (s, 1H), 8.19 (s, 2H).

(3,5-diiodophenyl)methanol (41): To a solution of compound **40** (51.0 g, 136 mmol) in THF (150 mL) was added BH_3•THF (1 M, 1.13 L) at 0°C. The mixture was stirred at 20°C for 2 h. TLC (petroleum ether/ethyl acetate = 3/1, product R_f = 0.8) indicated compound **40** (R_f = 0.0) was

consumed completely. The reaction was quenched with H_2O (1.00 L) at 0°C, extracted with ethyl acetate (500 mL x 3). The organic layers were combined, dried over Na_2SO_4, filtered and concentrated under vacuum. The residue was purified by column chromatography (SiO_2, petroleum ether/ethyl acetate = 50/1 to 3/1). The desired fraction was concentrated to remove low boiling point solvent at 35 °C. Compound **41** (24.3 g, 49.5% yield) was obtained as a light yellow solid.

^{1}H NMR: $CDCl_3$ 400MHz, δ: 7.98 (s, 1H), 7.68 (d, J = 1.2 Hz, 2H), 4.62 (s, 2H).

***Tert*-butyl((3,5-diiodobenzyl)oxy)dimethylsilane (42):** To a solution of compound **41** (14.0 g, 38.9 mmol) in DMF (98.0 mL) was added TBSCl (8.79 g, 58.3 mmol, 7.15 mL) and imidazole (3.97 g, 58.3 mmol). The mixture was stirred at 25°C for 2 h. TLC (petroleum ether/ethyl acetate = 5/1, product R_f = 0.98) indicated compound **41** (R_f = 0.3) was consumed completely and two new spots formed. The mixture was quenched with water (100 mL) and extracted with ethyl acetate (80.0 mL x 3). The organic layers were combined, dried over Na_2SO_4, filtered and concentrated under vacuum. The residue was purified by column chromatography (SiO_2, petroleum ether). The desired fraction was concentrated to remove low boiling point solvent at 35°. Compound **42** (16.5 g, 89.4% yield) was obtained as a light-yellow oil.

^{1}H NMR: $CDCl_3$ 400MHz, δ: 7.93 (s, 1H), 7.62 (s, 2H), 4.63 (d, J = 0.8 Hz, 2H), 0.95 (s, 9H), 0.11 (s, 6H).

1-(3-(((*tert*-butyldimethylsilyl)oxy)methyl)-5-iodophenyl)-2,2,2-trifluoroethan-1-one (43): To a solution of compound **42** (19.5 g, 41.1 mmol) in THF (200 mL) was added dropwise n-BuLi (2.5 M, 17.3 mL) at -65°C over 15 min. After addition, the mixture was stirred at this temperature for 15 min. Then CF_3COOEt (11.7 g, 82.3 mmol, 11.3 mL) in THF (40.0 mL) was added dropwise at -65 °C. The resulting mixture was stirred at -65°C for 30 min. TLC (petroleum ether, product R_f = 0.0) indicated compound **42** (R_f = 0.8) was consumed completely and one main new spot formed. The reaction mixture was quenched by addition saturated NH_4Cl (200 mL) at 0 °C, and extracted with EtOAc mL (300 mL x 3). The combined organic layers were washed with brine (200 mL), dried over Na_2SO_4, filtered and concentrated under vacuum. The residue was purified by column chromatography (SiO_2, petroleum ether/ethyl acetate = 50/1 to 3/1). The desired fraction was

concentrated to remove low boiling point solvent at 35 °C. Compound **43** (10.0 g, 54.7% yield) was obtained as a light-yellow oil.

^{1}H NMR: $CDCl_3$ 400MHz, δ: 8.26 (s, 1H), 7.80 (s, 2H), 4.77 (s, 2H), 0.95 (s, 9H), 0.14 (s, 6H).

(*Z*)-1-(3-(((*tert*-butyldimethylsilyl)oxy)methyl)-5-iodophenyl)-2,2,2-trifluoroethan-1-one oxime (44): To a solution of compound **43** (6.55 g, 14.7 mmol) in EtOH (30.0 mL) and Pyridine (30.0 mL) was added NH_2OH•HCl (1.54 g, 22.1 mmol). The mixture was stirred at 60°C for 18 h. TLC (petroleum ether/ethyl acetate = 5/1, product R_f = 0.7) indicated compound **43** (R_f = 0.5) was consumed completely and one main new spot formed. The mixture was dissolved in ethyl acetate (80.0 mL) and washed with H_2O (60.0 mL x 3), brine (50.0 mL), dried over Na_2SO_4, filtered and concentrated under vacuum. Compound **44** (6.77 g, 100% yield) was obtained as a light-yellow oil.

1-(3-(((*tert*-butyldimethylsilyl)oxy)methyl)-5-iodophenyl)-2,2,2-trifluoroethan-1-one *O*-tosyl oxime (45): Compound **44** (9.97 g, 21.1 mmol) was dissolved in DCM (70.0 mL) and cooled to 0°C, then TEA (6.59 g, 65.1 mmol, 9.06 mL), DMAP (265 mg, 2.17 mmol) and TosCl (6.21 g, 32.6 mmol) were added, the mixture was stirred at 23°C for 5 h. TLC (petroleum ether/ethyl acetate = 10/1, product R_f = 0.7) indicated compound **44** (R_f = 0.2) was consumed completely and one main new spot formed. The mixture was dissolved in ethyl acetate (80.0 mL) and washed with H_2O (60.0 mL x 3), brine (50.0 mL), dried over Na_2SO_4, filtered and concentrated under vacuum. The residue was purified by column chromatography (SiO_2, petroleum ether/ethyl acetate = 50/1 to 5/1). The desired fraction was concentrated to remove low boiling point solvent at 40 °C. Compound **45** (5.80 g, 43.5% yield) was obtained as a light-yellow oil.

^{1}H NMR: $CDCl_3$ 400MHz, δ: 8.26 (s, 1H), 7.80-7.91 (m, 1H), 7.90-7.88 (m, 3H), 7.82 (s, 1H), 7.59 (s, 1H), 7.41 (s, 1H), 7.40-7.38 (m, 4H), 7.31 (s, 1H), 4.77 (s, 1H), 2.50-2.47 (m, 5H), 1.54 (s, 3H), 0.97-0.94 (m, 9H), 0.13-0.08 (m, 6H).

3-(3-(((*tert*-butyldimethylsilyl)oxy)methyl)-5-iodophenyl)-3-(trifluoromethyl)-3*H*-diazirine (46): To a solution of compound **45** (5.80 g, 9.45 mmol) in DCM (29.0 mL) was added NH_3 in MeOH (7 M, 21.6 mL) at -50°C. The mixture was stirred at 25°C for 20 h. TLC (petroleum ether/ethyl acetate = 10/1, product R_f = 0.6) indicated compound **45** (R_f = 0.7) was consumed

completely and one main new spot formed. And then Et_3N (2.87 g, 28.4 mmol, 3.95 mL) was added at -40°C. A saturated solution of iodine in DCM (80.0 mL) was added drop wise until the brown color of iodine persisted for at least 1 min. TLC (petroleum ether, product $R_f = 0.5$) indicated compound **45** ($R_f = 0.0$) was consumed completely and one main new spot formed. The mixture was quenched with water (30.0 mL), extracted with ethyl acetate (20.0 mL x 3), the organic layers were combined, washed with brine (40.0 mL), dried over Na_2SO_4, filtered and concentrated under vacuum. The residue was purified by column chromatography (SiO_2, petroleum ether). The desired fraction was concentrated to remove low boiling point solvent at 35°C. Compound 4**6** (2.34 g, 54.2% yield) was obtained as a light yellow oil.
^{1}H NMR: $CDCl_3$ 400MHz, δ: 7.71 (s, 1H), 7.32 (s, 1H), 7.21 (s, 1H), 4.69 (s, 2H), 0.95 (s, 9H), 0.11 (s, 6H).

3-(3-(((*tert*-butyldimethylsilyl)oxy)methyl)-5-((trimethylsilyl)ethynyl)phenyl)-3-(trifluoromethyl)-3*H*-diazirine (47): A mixture of compound 4**6** (2.34 g, 5.13 mmol), compound **18A** (579 mg, 5.90 mmol, 816 µL), CuI (97.7 mg, 513 µmol), $Pd(PPh_3)Cl_2$ (180 mg, 256 µmol) and Et_2NH (375 mg, 5.13 mmol, 528 µL) in THF (24.0 mL) was degassed and purged with N_2 for 3 times. Then the mixture was stirred at 25°C for 3.5 h under N_2 atmosphere. TLC (petroleum ether, product $R_f = 0.4$) indicated compound 4**6** ($R_f = 0.5$) was consumed completely and one new main spot formed. The reaction mixture was filtered and the filter cake was washed with EtOAc (5.00 mL x 3). The combined filtrate was concentrated under vacuum. The mixture was quenched with water (20.0 mL), extracted with ethyl acetate (20.0 mL x 3), the organic layers were combined, washed with brine (30.0 mL), dried over Na_2SO_4, filtered and concentrated under vacuum. The residue was purified by column chromatography (SiO_2, petroleum ether). The desired fraction was concentrated to remove low boiling point solvent at 35 °C. Compound **47** (1.90 g, 86.8% yield) was obtained as a light-yellow oil.
^{1}H NMR: $CDCl_3$ 400 MHz, δ: 7.41 (s, 1H), 7.21 (s, 1H), 7.10 (s, 1H), 4.70 (s, 2H), 0.95 (s, 9H), 0.25 (s, 9H), 0.10 (s, 6H).

3-(3-(((*tert*-butyldimethylsilyl)oxy)methyl)-5-ethynylphenyl)-3-(trifluoromethyl)-3*H*-diazirine (48): To a solution of compound **47** (1.90 g, 4.45 mmol) in THF (20.0 mL) was added TBAF (1 M, 11.1 mL) at 0°C. The mixture was stirred at 25°C for 1.5 h. TLC (petroleum

ether/ethyl acetate = 5/1, product R_f = 0.4) indicated compound **47** (R_f = 0.98) was consumed completely and one new spot formed. The crude mixture was diluted with EtOAc (20.0 mL) and washed with saturation NH_4Cl (20.0 mL), dried over Na_2SO_4, filtered and concentrated under vacuum. The residue was purified by column chromatography (SiO_2, petroleum ether/ethyl acetate = 5/1). The desired fraction was concentrated to remove low boiling point solvent at 35°C. Compound **48** (900 mg, 84.1% yield) was obtained as a yellow oil.
^{1}H NMR: $CDCl_3$ 400 MHz, δ: 7.54 (s, 1H), 7.26 (s, 1H), 7.18 (s, 1H), 4.71 (s, 2H), 3.14 (s, 1H).

3-(3-(bromomethyl)-5-ethynylphenyl)-3-(trifluoromethyl)-3*H*-diazirine (49): To a solution of compound **48** (500 mg, 2.08 mmol) in DCM (10.0 mL) was added CBr_4 (794 mg, 2.39 mmol,) and PPh_3 (628 mg, 2.39 mmol) at 0°C. The mixture was stirred at 20°C for 2 h. TLC (petroleum ether/ethyl acetate = 5/1, product R_f = 0.5) indicated compound **48** (R_f = 0.3) was consumed completely and one new main spot formed. The reaction mixture was concentrated under vacuum. The residue was purified by column chromatography (SiO_2, petroleum ether/ethyl acetate = 10/1 to 3/1). The desired fraction was concentrated to remove low boiling point solvent at 35°C. Compound **49** (400 mg, 63.4% yield) was obtained as a light-yellow oil.
^{1}H NMR: $CDCl_3$ 400 MHz, δ: 7.57 (s, 1H), 7.27 (s, 1H), 7.16 (s, 1H), 4.42 (s, 2H), 3.16 (s, 1H).

***N*-(2-(4-((3-ethynyl-5-(3-(trifluoromethyl)-3*H*-diazirin-3-yl)benzyl)oxy)-2-(5-hydroxypent-1-yn-1-yl)phenyl)-4*H*,10*H*-benzo[*f*]pyrazolo[5,1-*c*][1,4]oxazepin-7-yl)acetamide (G`7305):** A mixture of compound **38** (100 mg, 240 µmol), compound **49** (109 mg, 359 µmol) and K_2CO_3 (99.3 mg, 718 µmol) in DMF (6.00 mL) was degassed and purged with N_2 for 3 times. The mixture was stirred at 25°C for 16 h under N_2 atmosphere. LC-MS and HPLC analysis showed compound **38** was consumed completely and one main peak of the desired compound was detected. The reaction mixture was filtered. The filtrate was purified by HPLC using a 5-micron Waters Xbridge BEH C18 column with ID of 100 mm X 25 mm. The mobile phase was composed of water (with 10mM NH_4HCO_3) and a gradient of ACN from 68% - 85% over 10 min.

The collected fractions were concentrated to remove low boiling point solvent at 35°C, then freeze-dried. Compound **G`7305** (43.0 mg, 28.0% yield, 99.8% purity) was obtained as an off-white solid.

^{1}H NMR: DMSO 400MHz, δ: 9.13 (s, 1H), 6.91-6.87 (m, 2H), 6.57 (s, 1H), 6.47-6.42 (m, 3H), 6.31-6.29 (m, 1H), 6.21-6.18 (m, 2H), 6.12 (s, 1H), 4.92 (s, 1H), 4.73 (s, 2H), 4.51 (s, 2H), 4.35 (s, 2H), 3.72-3.69 (m, 1H), 3.58 (s, 1H), 2.70-2.65 (m, 2H), 1.18 (s, 3H), 0.87-0.84 (m, 2H). **LCMS:** (M+H): 640.3, calculated 639.21.
