# Supplementary material for: Selective activation of PFKL suppresses the phagocytic oxidative burst
Source: Cell. Author manuscript; Available in PMC 2022 Jan 31. (PMC8802628; doi:10.1016/j.cell.2021.07.004)
Supplement: 4 [file NIHMS1770610-supplement-4.pdf]

Supplemental figures

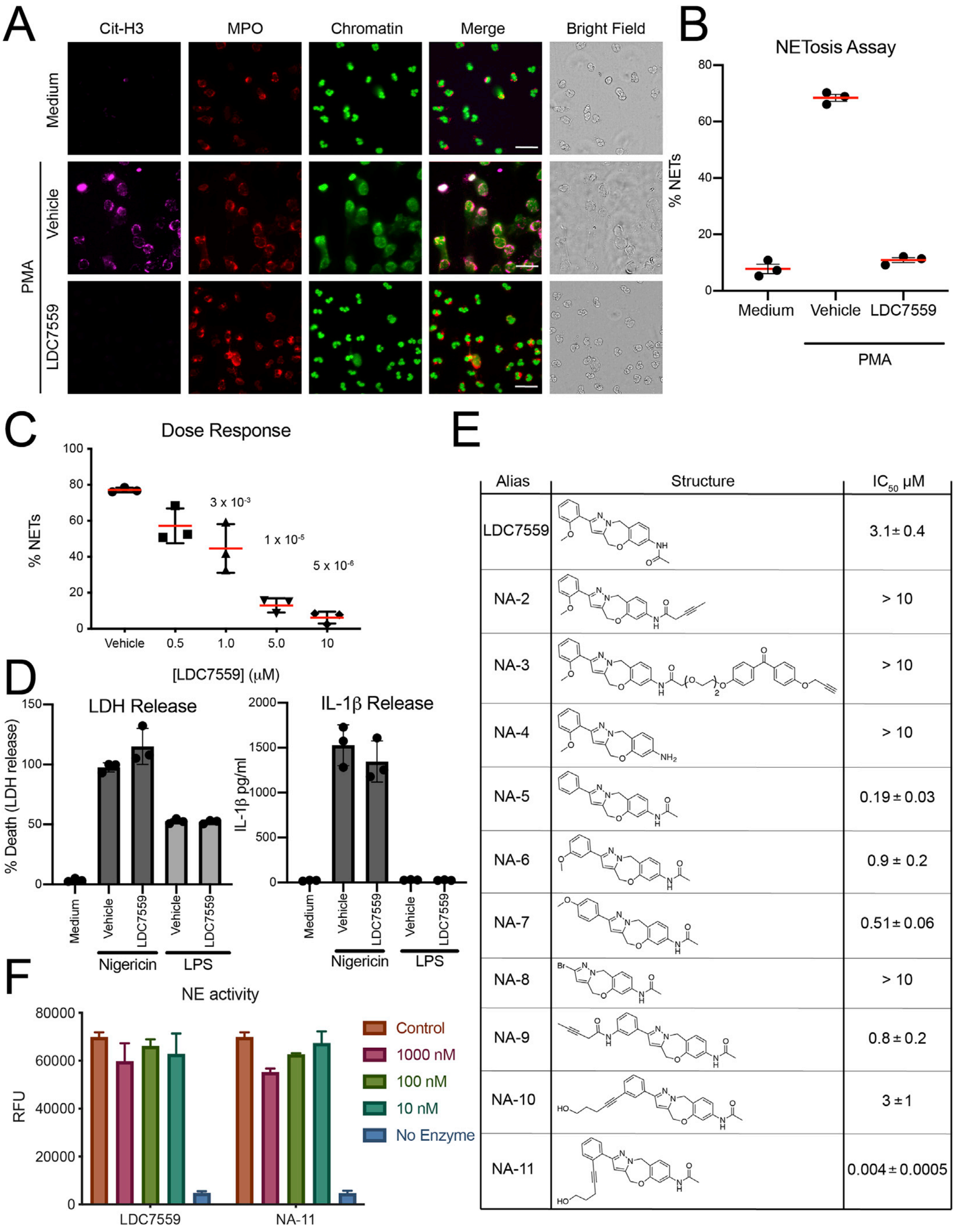

Figure S1. LDC7559 or NA-11 inhibits NOX2-dependent NETosis in human neutrophils, related to Figure 1

(A) Immunolabeling of citrullinated histone H3 (Cit-H3) and myeloperoxidase (MPO) in human PMNs. Chromatin is stained with SYTOX Green. Scale bar, 10 μm. Results representative of 3 independent experiments. (B) Quantification of the NETosis in (A). Data are the mean ± s.e.m. of PMNs from 3 donors. (C) Percentage of PMNs undergoing PMA-induced NETosis. Data are the mean ± s.e.m. of PMNs from 3 donors. P values (two-way ANOVA, means compared to vehicle) are shown if $p < 0.05$. (D) LDH (left) or IL-1β (right) released from THP-1 cells. Bars indicate the mean ± s.d. of 3 independent experiments. (E) LDC7559 SAR analogs. Inhibition of NETosis was quantified by automated DNA area analysis and data points were fitted to a dose-response curve (Prism). IC_{50} values are the mean ± s.d. of 3 independent experiments. (F) Activity of recombinant NE. Bars indicate the mean ± s.d. of 3 independent experiments.

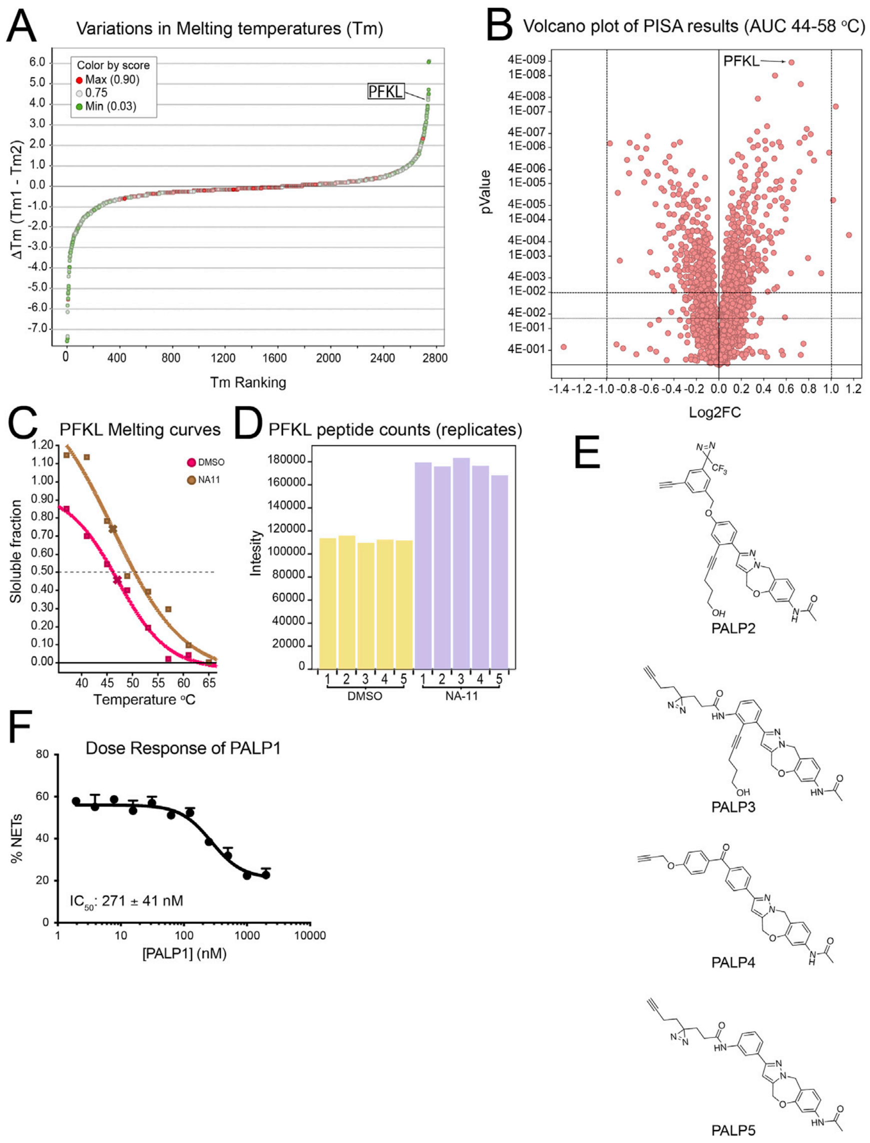

Figure S2. TPP analysis of global interactors of NA-11, related to Figure 2

(A) Waterfall plot of variation in melting temperature (Tm). Difference in Tm of NA-11 treated (Tm1) versus untreated (Tm2) lysates are plotted on the y axis. Data points are colored by a quality score that represents the confidence in the measured differences and is described in the STAR Methods section protein and data analysis. (B) Volcano plots of fold changes in area under the graph (AUG) from 44-58°C. (C) Melting curves for PFKL. (D) Peptide counts of PFKL from 5 replicates. (E) Structures of photoreactive probes. (F) Percentage of PMNs undergoing PMA-induced NETosis. An IC_{50} of 271 ± 41 nM for PALP1 was calculated from curve fitting. Data points represent the mean ± s.d. of cells from 3 donors.

CellPress
OPEN ACCESS

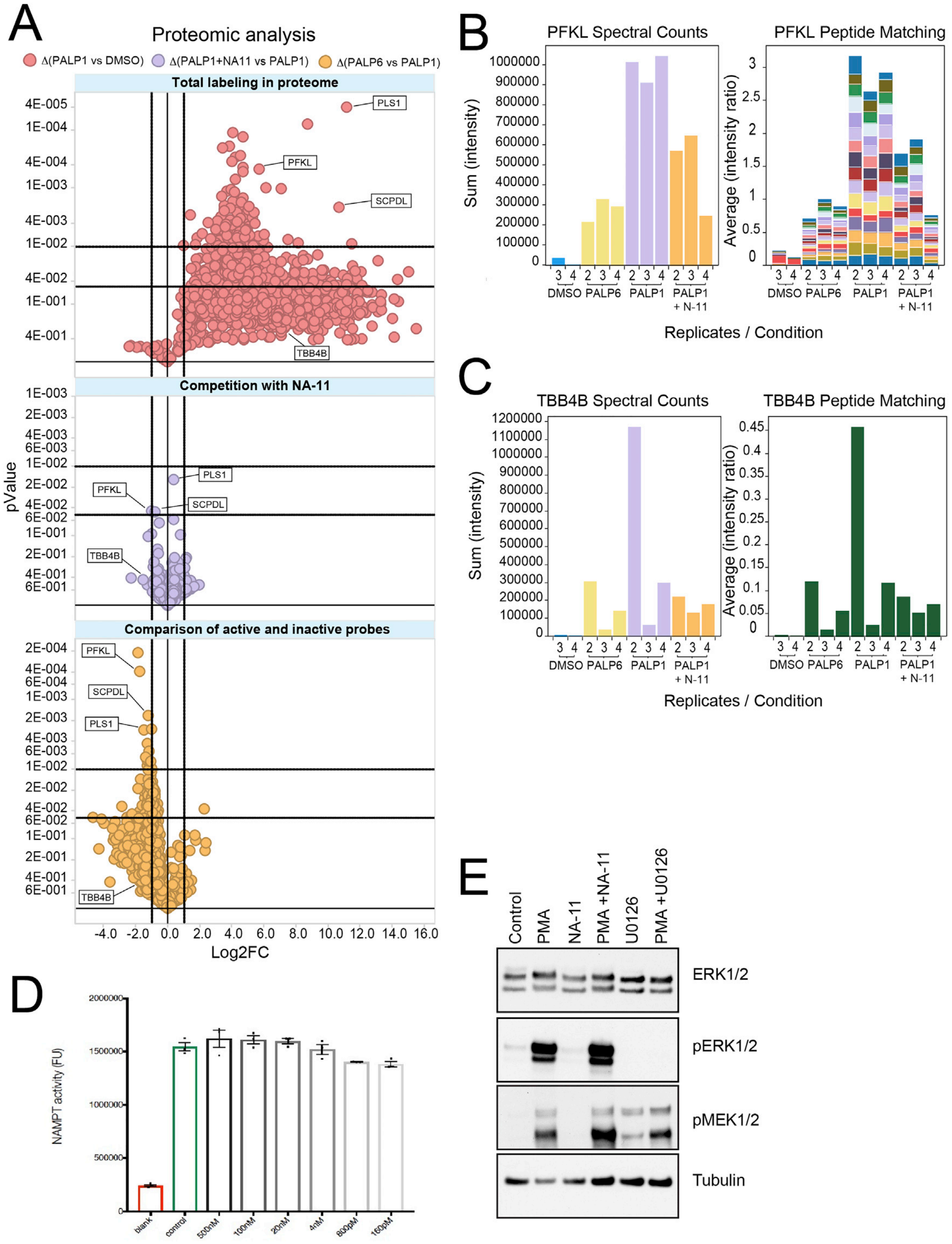

Figure S3. Chemoproteomic analysis of covalent crosslinking using photolabeling probe PALP1, related to Figure 2

(A) Volcano plots of the fold change in peptides detected after affinity pull-downs using PALP1 and PALP6. P values were calculated by Student's t test across three replicates. (B and C) Spectral counts of all PFKL (B) or TBB4B (C) peptides across replicates (left) or with individual peptides distinguished by color (right). (D) Activity of recombinant NAMPT. Bars represent the mean ± s.d. of 3 independent experiments. (E) western blots of PMNs pretreated with 0.5 μM NA-11 or 1 μM MEK1/2 inhibitor U0126 for 30 min, and then activated with 50 nM PMA for 30 min. Results are representative of 3 independent experiments.

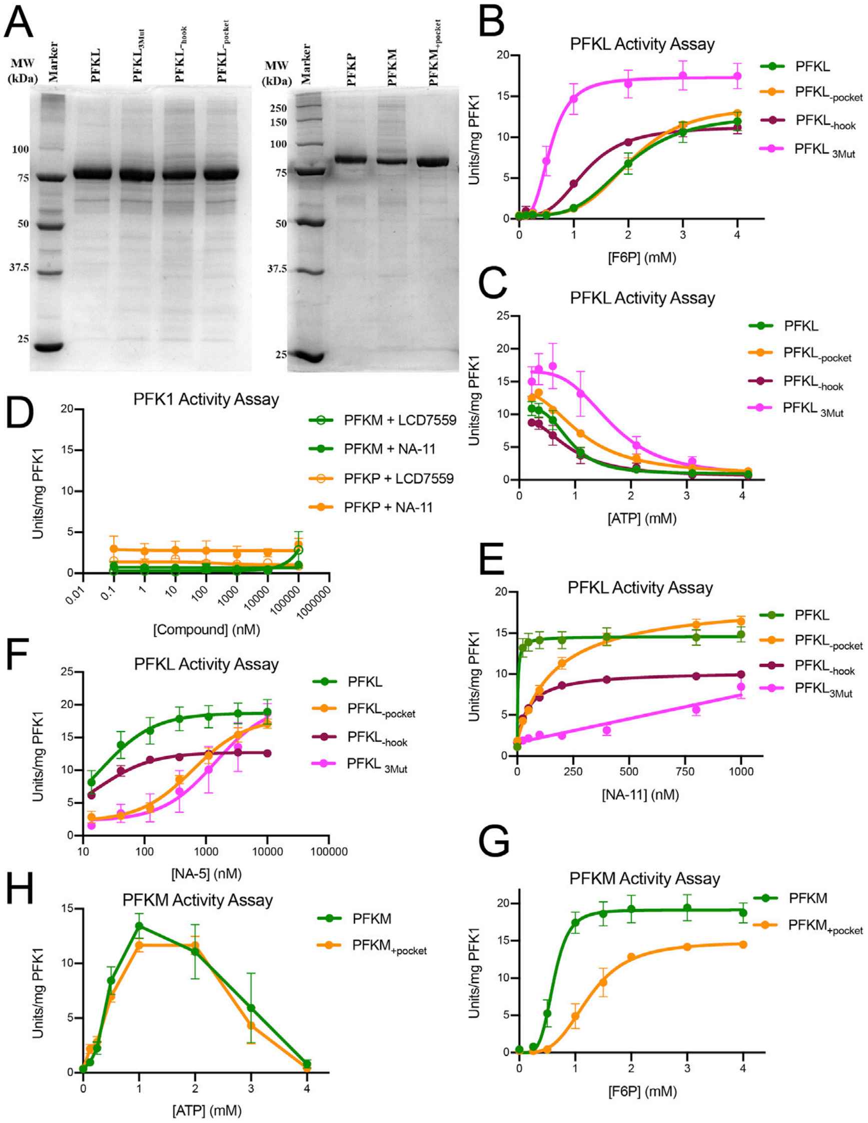

Figure S4. Activity of purified recombinant PFK1, related to Figure 3

(A) Coomassie blue staining of purified wild-type and mutant PFKL, PFKM, and PFKP. (B, C, E and F) Activity of wild-type and mutant PFKL in the presence of F6P (B), ATP (C), NA-11 (E), and NA-5 (F). (D, G and H) Activity of wild-type and mutant PFKM in the presence of NA-11 and LDC7559 (D), F6P (G) and ATP (H). Data points in (B-H) represent the mean ± s.e.m. Assay conditions are listed in Tables 1 and S1.

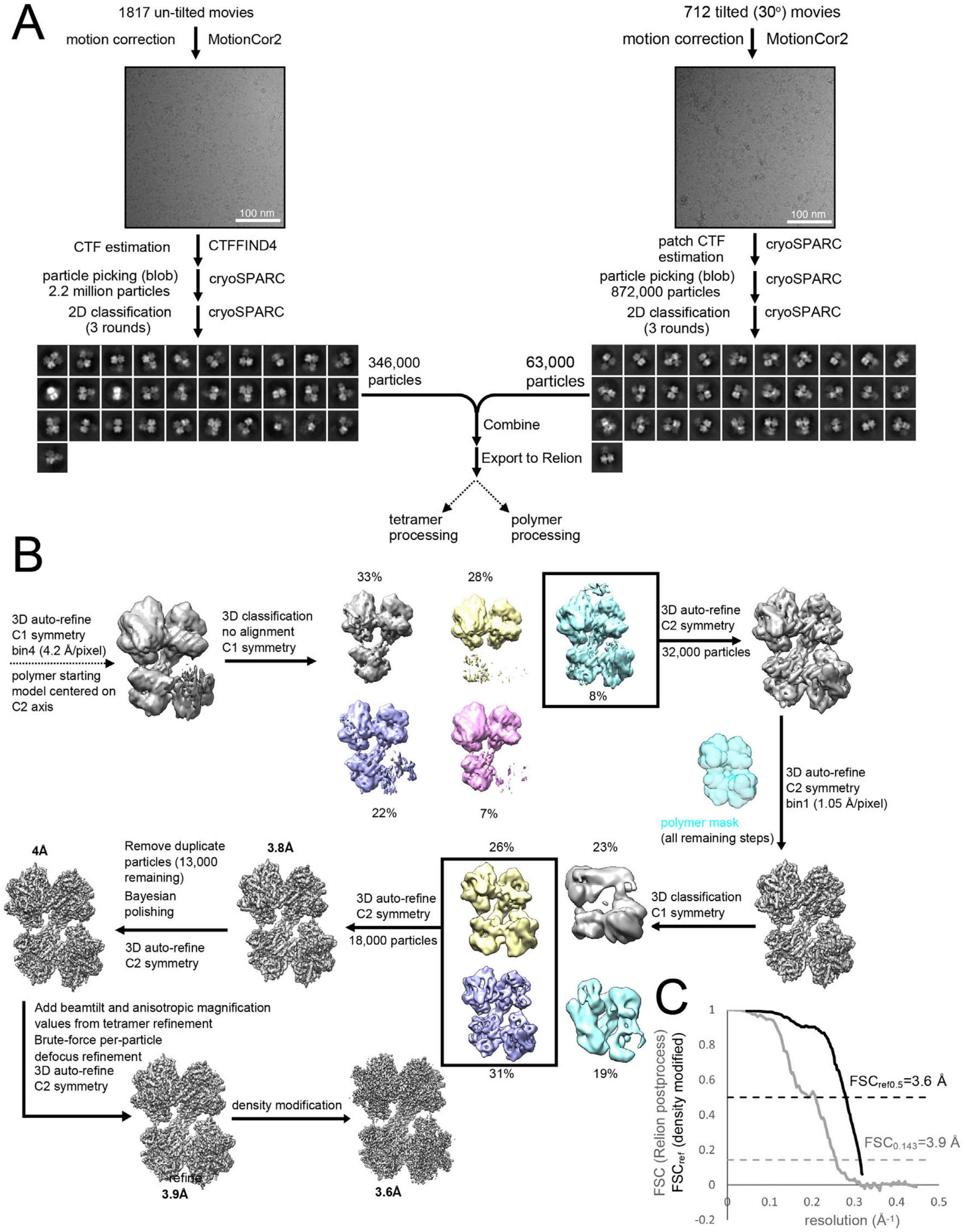

Figure S5. Cryo-EM processing flowchart, related to Figure 3

(A) Cryo-EM processing flowchart for motion correction, CTF estimation, particle picking, and 2D classification of un-tilted and tilted images. (B) Cryo-EM processing flowchart for 3D refinement of NA-11-bound PFKL polymers. Continues from the end of the flowchart in (A). (C) Fourier Shell Correlation (FSC) curves for the NA-11-bound PFKL polymer. The half-map FSC curve from relion postprocessing (gray) and FSC_{ref} curve after density modification (black) and corresponding resolution estimates are shown.

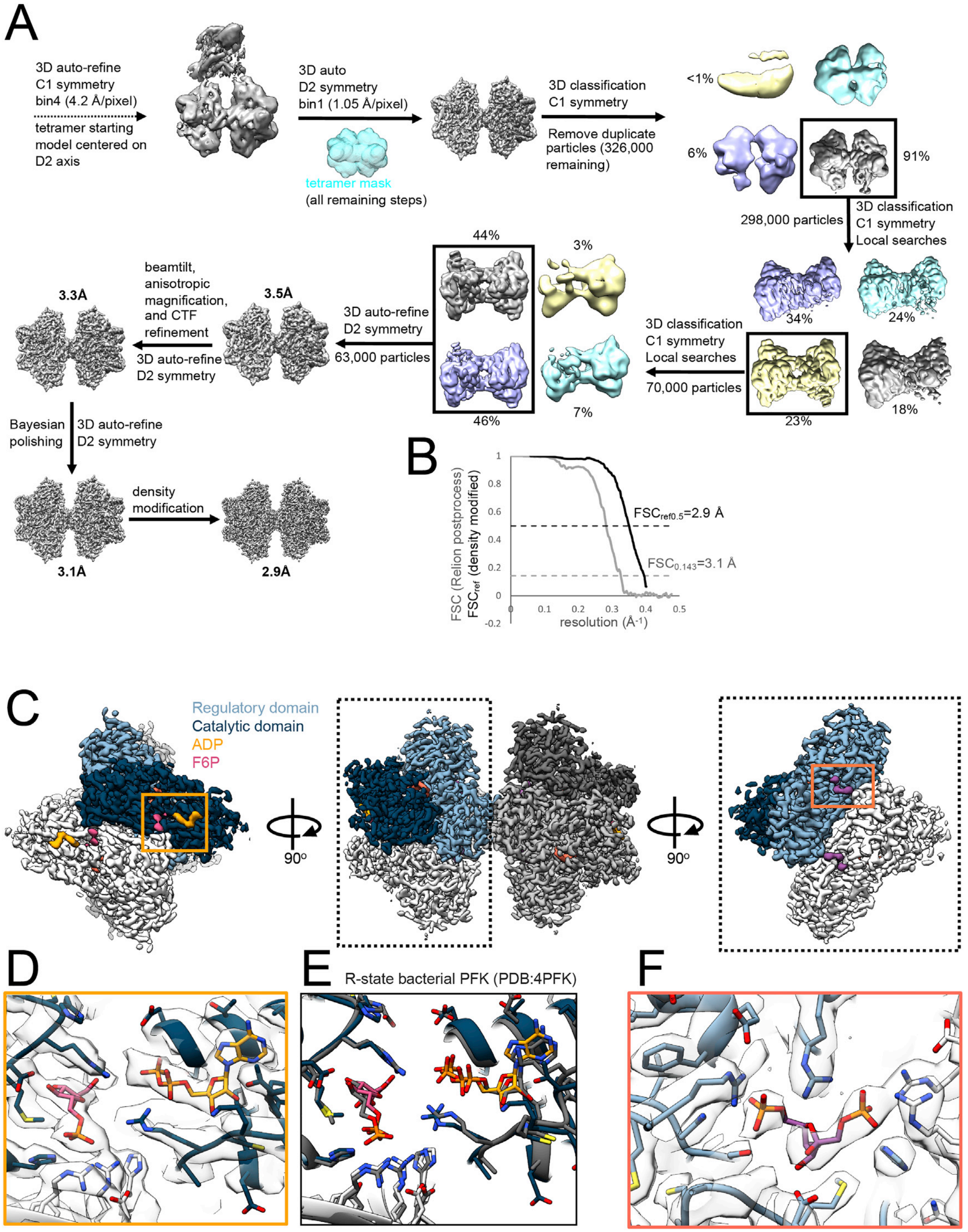

Figure S6. Cryo-EM processing of PFKL tetramers, related to Figure 3

(A) Cryo-EM processing flowchart for 3D refinement of NA-11-bound PFKL tetramers. Continues from the end of the flowchart in Figure S5A. (B) FSC curves for the NA-11-bound PFKL tetramer. The half-map FSC curve from relion postprocessing (gray) and FSC_{ref} curve after density modification (black) and corresponding resolution estimates are shown. (C) Three views of the NA-11-bound PFKL tetramer show the structure of the catalytic and allosteric sugar effector sites of PFKL. (D). A magnified view of the yellow box in (C), showing ADP (yellow) and F6P (pink) bound to the catalytic site. (E) Overlay of the catalytic sites of NA-11-bound PFKL and R-state bacterial PFK (PDB code 4PFK). (F) Zoomed-in view of the orange box in (C), showing FBP (purple) bound to the sugar effector site.

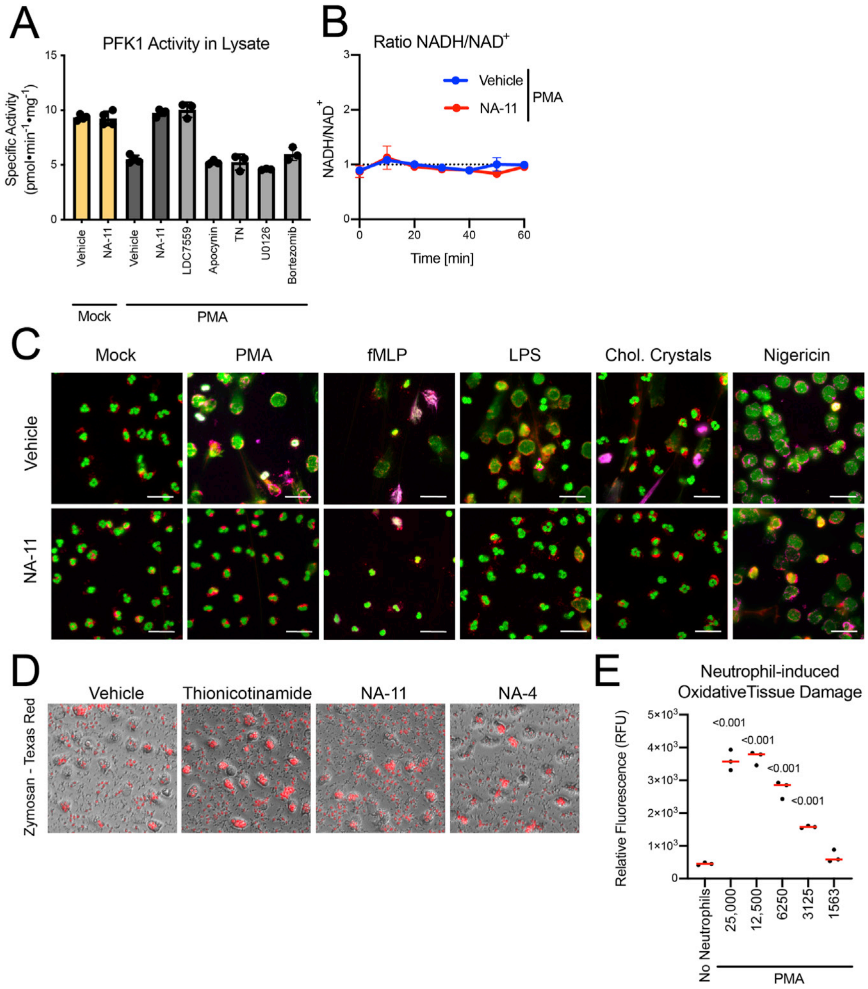

Figure S7. NA-11 activates PFKL to prevent the oxidative burst and impair neutrophil defenses, related to Figures 4 and 6

(A) PFK1 activity in PMN lysates. Cells were pretreated with 0.5 μM NA-11, 5 μM LDC7559, 100 μM apocynin, 200 μM thionicotinamide (TN), 1 μM MEK inhibitor U0126, or DMSO vehicle for 30 min, or 1 μM proteasome inhibitor bortezomib for 1 h, and then activated with 50 nM PMA for 30 min where indicated. Bars represent the mean ± s.d. of cells from 3 donors. (B) NADH:NAD$^+$ ratio in PMA-activated PMNs. Cells were pretreated with 0.5 μM NA-11 or DMSO vehicle for 30 min, and then activated with 50 nM PMA for 30 min. Data are the mean ± s.d. of cells from 3 donors. (C) Immunolabeling of citrullinated histone H3 (magenta) and myeloperoxidase (red) in PMNs pretreated with 0.5 μM NA-11 or DMSO vehicle for 30 min and then stimulated for 4 h with 50 nM PMA, 10 μg/mL extracellular LPS, 0.5 mg/mL cholesterol crystals, or 25 μM nigericin. Chromatin is stained green. Scale bar, 10 μm. Results are representative of 3 independent experiments. (D) Fluorescence micrographs of PMNs pretreated with 0.5 μM NA-11, 200 μM thionicotinamide (TN), 0.5 μM NA-4, or DMSO vehicle for 30 min, and then incubated with serum-opsonized Texas Red-conjugated zymosan particles (10 μg/mL) for 2 h. (E) Graph indicates migration of FITC-labeled dextran across a monolayer of human bronchial epithelial cells when co-cultured for 6 h with PMA-activated PMNs. Numbers on the x axis indicate the number of PMNs added. Data are the mean ± s.e.m. of cells from 3 donors. P values (two-way ANOVA, means compared to no neutrophils) are shown when $p < 0.05$.
